# Disruption of DYRK1A-induced hyperphosphorylation of amyloid-beta and tau protein in Alzheimer’s disease: An integrative molecular modeling approach

**DOI:** 10.3389/fmolb.2022.1078987

**Published:** 2023-01-19

**Authors:** Rohit Shukla, Anuj Kumar, David J. Kelvin, Tiratha Raj Singh

**Affiliations:** ^1^ Department of Biotechnology and Bioinformatics, Jaypee University of Information Technology (JUIT), Waknaghat, Himachal Pradesh, India; ^2^ Centre for Excellence in Healthcare Technologies and Informatics (CEHTI), Jaypee University of Information Technology (JUIT), Waknaghat, Himachal Pradesh, India; ^3^ Laboratory of Immunity, Shantou University Medical College, Shantou, China; ^4^ Department of Microbiology and Immunology, Canadian Centre for Vaccinology CCfV, Faculty of Medicine, Dalhousie University, Halifax, NS, Canada

**Keywords:** Alzheimer’s disease, neurofibrillary tangles, DYRK1A, virtual screening, molecular docking, molecular dynamics simulation, principal component analysis

## Abstract

Alzheimer’s disease (AD) is a neurological disorder caused by the abnormal accumulation of hyperphosphorylated proteins. Dual-specificity tyrosine phosphorylation-regulated kinase 1A (DYRK1A) is a dual phosphorylation enzyme which phosphorylates the amyloid-β (Aβ) and neurofibrillary tangles (NFTs). A high throughput virtual screening approach was applied to screen a library of 98,071 compounds against DYRK1A using different programs including AutoDock Vina, Smina, and idock. Based on the binding affinities, we selected 330 compounds for absorption, distribution, metabolism, excretion, and toxicity (ADMET) analysis. Various pharmacokinetics parameters were predicted using the admetSAR server, and based on the pharmacokinetics results, 14 compounds were selected for cross-docking analysis using AutoDock. Cross-docking analysis revealed four compounds, namely, ZINC3843365 (−11.07 kcal/mol^−1^), ZINC2123081 (−10.93 kcal/mol^−1^), ZINC5220992 (−10.63 kcal/mol^−1^), and ZINC68569602 (−10.35 kcal/mol^−1^), which had the highest negative affinity scores compared to the 10 other molecules analyzed. Density functional theory (DFT) analysis was conducted for all the four top-ranked compounds. The molecular interaction stability of these four compounds with DYRK1A has been evaluated using molecular dynamics (MD) simulations on 100 nanoseconds followed by principal component analysis (PCA) and binding free energy calculations. The Gibbs free energy landscape analysis suggested the metastable state and folding pattern of selected docking complexes. Based on the present study outcome, we propose four antagonists, viz., ZINC3843365, ZINC2123081, ZINC5220992, and ZINC68569602 as potential inhibitors against DYRK1A and to reduce the amyloid-β and neurofibrillary tangle burden. These screened molecules can be further investigated using a number of *in vitro* and *in vivo* experiments.

## 1 Introduction

Alzheimer’s disease (AD) is a common neurodegenerative disease and comprises 60%–70% of all dementia cases. Dementia affects more than 50 million people worldwide. There is an urgent need to develop new therapeutics for AD because the currently available drugs cannot stop the progression of the disease. If this disease progression is not altered by the invention of new drugs to halt or slow down the progression of AD, then it is estimated that the number of cases will double by 2030 (Zeisel et al., 2020). AD is characterized by the abnormal accumulation of proteins in the brain. Amyloid-beta (A*β*) plaques and neurofibrillary tangles (NFTs) are the key biomarkers for disease identification (Kumar et al., 2016). A*β* plaques are made by the abnormal cleavage of the amyloid precursor protein (APP), while the NFTs are made by the accumulation of the hyperphosphorylated tau protein (Iqbal et al., 2016). The microtubule-associated protein tau (MAPT) provides the stability of the microtubules by stabilizing between the α-microtubules and β-microtubules. In the disease condition, the hyperphosphorylated tau protein is detached from the microtubules and forms oligomers. Tauopathies is an umbrella term that describes the tau pathology in several neurodegenerative diseases, which includes AD and Parkinson’s disease (PD) (Iqbal et al., 2016). The tauopathies are not only involved in AD, they are also involved in Pick’s disease, progressive supranuclear palsy (PSP), and frontotemporal lobar degeneration with tau inclusions (FTLD‐tau) (Melchior et al., 2019). In AD, the amyloid beta-induced tau aggregation is also observed; however, targeting only the Aβ formation failed to stop the disease progression in AD patients (Giacobini and Gold, 2013; Holtzman et al., 2016). Targeting tau will be a key approach for altering AD progression. The spatiotemporal pattern of tau pathology in AD is highly correlated with brain atrophy and observed cognitive decline (Giannopoulos et al., 2018). Tau acts as a neurotoxic protein due to high phosphorylation, resulting in oligomerization sequestration and ultimately forming NFTs by the aggregation (Spillantini and Goedert, 2013). Various kinases can hyperphosphorylate the tau protein at multiple epitopes; therefore, this excessively phosphorylated tau (pTau) protein becomes toxic and is found in higher concentrations (∼4–5 fold) in AD patients’ brains. In a normal brain, the phosphate concentration is 2–3 mol, while in the AD brain, this concentration is higher and was recorded to be as high as 7–8 mol (Iqbal et al., 2016). These insoluble and hyperphosphorylated forms of tau build the aggregated filamentous oligomers, and these are gradually deposited in the form of intraneuronal pretangles, and these NFTs are the major hallmark for all types of tauopathies (Morris et al., 2011; Bodea et al., 2016). Various strategies have been applied to alter the aggregation and spreading of tauopathies, and several compounds are also being tested in clinical trials (Verma et al., 2018). To date, the exact cause of tau toxicity has not been identified (Khanna et al., 2016). Hence, targeting the upstream regulation of tau protein is an appealing strategy. Tau protein has 85 phosphorylation sites (80 Ser/Thr and 5 Tyr), in which more than 40 epitopes are recognized to specifically phosphorylate in the AD brain by several kinases (Martin et al., 2013; Simic et al., 2016; Polanco et al., 2018). We have targeted glycogen synthase kinase 3 beta (Shukla et al., 2019; Shukla and Singh, 2021) and cyclin-dependent kinase 5 (Shukla and Singh, 2020a; Shukla and Singh, 2020b) and proposed several potential lead compounds using computational methods. Recently, several strategies to neutralize various kinases have been applied to find novel inhibitors against AD. Some strategies have identified potential therapeutics that are in clinical trials, while other potential therapeutics have failed at different stages (Le Corre et al., 2006; Vogel et al., 2009; Onishi et al., 2011; Lovestone et al., 2015).

The dual-specificity tyrosine phosphorylation-regulated kinase 1A (DYRK1A) belongs to the family of DYRK. This family consists of five different kinases (Duchon and Herault, 2016; Branca et al., 2017). The DYRK1A gene is located on chromosome 21; in Down’s syndrome, the protein expression is increased 1.5 fold (Tejedor and Hammerle, 2011). DYRK1A protein expression is tightly regulated in the brain, and its overexpression and underexpression directly correlates with intellectual disability (Luco et al., 2016). Several serine and threonine residues of the tau protein can be directly phosphorylated by DYRK1A (Ryoo et al., 2007; Azorsa et al., 2010). The formation of NFTs in Down’s syndrome and other diseases is due to the overexpression of DYRK1A protein (Liu et al., 2008). With the role of DYRK1A in tau pathology progression, it is also involved in APP/Aβ formation. The inhibition of DYRK1A in primary rat cortical neurons reduced the phosphorylation of tau proteins at several sites in a dose-dependent manner. It also reduced Aβ production in the HEK293 Aβ overexpressing cells (Coutadeur et al., 2015). In an earlier study, Wegiel et al. (2011) revealed that DYRK1A is responsible for the hyperphosphorylation of APP, which increases the APP affinity toward the BACE1 and gamma secretase, and due to this, the plaque formation and deposition level is also increased. Kimura et al. (2007) also suggest that DYRK1A forms a vicious cycle and facilitates Aβ accumulation. The postmortem of the AD brain also showed overexpression of DYRK1A, and these findings are consistent with the previously described results (Ferrer et al., 2005). All these studies suggest that the inhibition of the activity of DYRK1A could be a potential approach to reduce Aβ and NFT levels.

**FIGURE 1:**
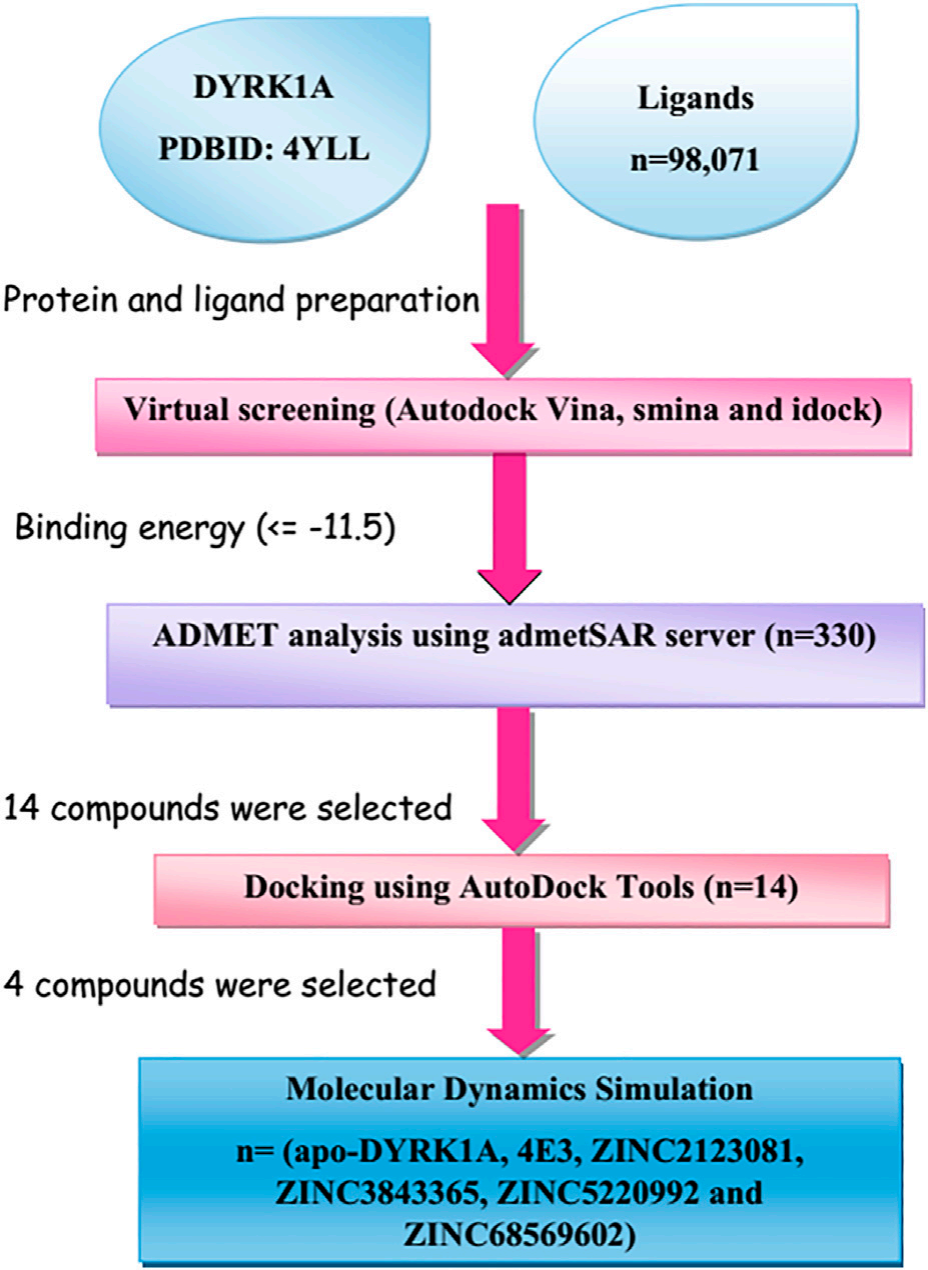
Representation of the brief methodology used in the present study to screen the library of 98,071 ligands against DYRK1A using the high-throughput virtual screening approach, followed by MD simulations of the four best lead compounds on 100 ns.

New advances in computer-aided drug design (CADD)-based methods have accelerated biologists’ efforts to identify lead drug compounds against a plethora of contagious diseases (Baig et al., 2018). These cutting-edge methods are capable of screening out large compound libraries by minimizing the cost and time in a significant manner. In this study, we screened the library of natural compounds against DYRK1A with the fruitful utilization of CADD methods to screen the potential inhibitors. After screening, we selected 14 compounds and performed cross-docking for validating the screening studies. Finally, we carried out the molecular dynamics (MD) simulation of 100 ns and predicted that ZINC3843365, ZINC2123081, ZINC5220992, and ZINC68569602 can act as novel inhibitors against DYRK1A. The complete methodology is shown in Figure 1.

## 2 Materials and methods

### 2.1 Ligand selection

The subset of the natural dataset was considered for this study and retrieved from the ZINC12 database (http://zinc12.docking.org/). The ZINC database (Sterling and Irwin, 2015) comprises millions of compounds from diverse families and diverse vendors. These compounds are ready to use and can be downloaded in various chemical file formats. We downloaded a subset *n* = 98,071 for the virtual screening in the mol2 file format, which is derived from a complete natural-compound catalog. These 98,071 compounds are filtered based on Lipinski’s rule of five (ROF) criteria from the complete natural compounds’ dataset (*n* = 178,231) (Benet et al., 2016).

ROF: Molecular weight ≤500 D, CLogP ≤5.00, the number of hydrogen bond donors ≤5 and the number of hydrogen bond acceptors ≤10, and the number of rotatable bonds ≤10.

### 2.2 Protein selection

The protein structure of DYRK1A was extracted from the RCSB-Protein Data Bank (https://www.rcsb.org/) in the PDB format. This structure was considered on the basis of resolution and the co-crystallized inhibitor. Based on these parameters, we selected the DYRK1A crystal structure (PDB ID: 4YLL, 1.4 Å, X-ray) (Falke et al., 2015). The co-crystallized inhibitor 4E3 (10-bromo-2-iodo-11H-indolo (Iqbal et al., 2016) quinoline-6-carboxylic acid) showed an IC_50_ value of 120 nM in the inhibition assay (Falke et al., 2015). 4E3 was used as a positive control to compare the predicted hits.

### 2.3 Structure-based virtual screening

Before the virtual screening, the protein and ligands were prepared using AutoDock tools. The protein was minimized using Chimera 1.13.2 software (Pettersen et al., 2004). The Amber ff99 SB force field (Lindorff-Larsen et al., 2010) was used, and all the hydrogen atoms were added to the structure. The heteroatoms were removed from the structure; then, 100 steepest descent steps followed by 10 conjugate gradient steps were used for the energy minimization. Finally, prepared protein and ligands were converted to *pdbqt* files using PDB to PDBQT python file. Polar and non-polar hydrogen atoms were added, followed by the Kollman charges and atom types also assigned, respectively. In the case of ligand preparation, the Gasteiger charges and all hydrogen atoms were added with the rotatable bonds. Finally, the grid was set based on the co-crystallized ligand 4E3. The catalytic residues such as Phe170, Val173, Lys188, Phe238, Leu294, and Val306 of DYRK1A were selected for the grid preparation. The dimensions 20 × 50 × 38 Å in the x, y, and z directions in the grid were set. This grid was used for all screening software. It is always an acceptable fact that consensus resulting from more than two tools is more accurate than the result predictions from one software. Therefore, in this study, we used three tools for the virtual screening. All the compounds were screened using idock (Li et al., 2012), Smina—a fork of AutoDock Vina (https://sourceforge.net/projects/smina/), and AutoDock Vina (Trott et al., 2010). The screened compounds were shortlisted based on the binding affinity, and then, common compounds were selected for absorption, distribution, metabolism, excretion, and toxicity (ADMET) prediction.

### 2.4 Pharmacokinetics analysis

ADMET is a key parameter to approve a drug for clinical use. New advances in computation-based methods have accelerated the development of user-friendly algorithms and web servers to predict and annotate the drug likeness properties of small chemical molecules. In this study, for predicting the various pharmacokinetics descriptors, we used the automated admetSAR server (Cheng et al., 2012). The admetSAR server is widely used, and it predicts more than 50 different features of ADMET properties (Yang et al., 2019). ADMET uses the machine learning model and known information on various existing FDA-approved drugs. We shortlisted 330 compounds based on the virtual screening results and used them for ADMET analysis.

### 2.5 Molecular docking

The 14 compounds selected from the virtual screening and ADMET analysis were employed for cross-docking with the AutoDock program (Morris et al., 2009). AutoDock is widely used and freely available docking software (Shukla et al., 2018). In AutoDock, we used the dimensions 20 × 50 × 38 Å in the x, y, and z directions, the same grid used for the virtual screening parameters. Next, we generated 100 binding poses for each ligand by using the Lamarckian genetic algorithm. The binding poses were selected based on the binding affinity and their interaction with the key catalytic residues.

### 2.6 Density functional theory calculations

Density functional theory (DFT) analysis was completed to decipher the electron transport potential and electronic properties of the selected lead compounds (Matysiak, 2007; Zhenming et al., 2011). The geometry of all the ligands was optimized and then employed for the DFT calculation through ArgusLab software (V: 4.0.1). We used the PM3 force field for the minimization of the ligand. It minimized and optimized the geometry of the ligands in several cycles and then calculated the various parameters. The highest occupied molecular orbital (HOMO) and lowest unoccupied molecular orbital (LUMO) are commonly known as frontier molecular orbitals and were found to give extremely applicable information about electron density clouds around the molecule (Genc et al., 2015). HOMO and LUMO are considered as a non-bonding type and π molecular orbital, respectively. HOMO and LUMO are favorable for the electrophilic and nucleophilic attacks, respectively. E_HOMO_ and E_LUMO_ are the quantum chemical parameters, in which E_HOMO_ has the capability of donating the electron, while E_LUMO_ can accept the electron from the partner interactor. The higher value of E_HOMO_ signifies that the compound can easily donate the electron without substantial energy requirement and vacate the molecular orbital (Gece and Bilgiç, 2009). The ΔE (energy gap) represents the difference between the HOMO and LUMO energy levels and is calculated by the ΔE = E_LUMO_ − E_HOMO_ (Lu et al., 2010). It is a key parameter that defines the reactivity of the lead compounds toward the DYRK1A binding site. A lesser energy gap represents the reactivity of top-ranked compounds, which leads to the increased electron-donating efficiency and reflects that it donates electrons from the last occupied orbital with very less energy (Tripathy et al., 2019).

### 2.7 Conformational dynamics analysis

The molecular dynamics simulation (MDS) is a well-known technique for predicting the protein–ligand complex stability. Hence, we performed the 100 ns simulation for apo–DYRK1A, DYRK1A–4E3, DYRK1A–ZINC2123081, DYRK1A–ZINC3843365, DYRK1A–ZINC5220992, and DYRK1A–ZINC68569602 using GROMACS 2018.2 (Abraham et al., 2015). The ligand topology was generated using the PRODRG server (van Aalten et al., 1996), while the protein topology was generated by GROMACS using the GROMOS96 53a6 force field (Oostenbrink et al., 2004). All the complexes were solvated using the SPC water model, following the parameters described by Gajula et al. (2016). The systems were then neutralized by 6 Cl^-^ ions. Next, the steric hindrance and atomic clashes of all the systems were removed by energy minimization. NVT and NPT simulation of 1 ns were conducted to fix the volume, temperature, and pressure of the systems. All the systems were employed for the final run of 100 ns. Output trajectory was processed using the trajectory preprocessing tool called *trjconv*. After MD simulations, different statistical parameters including the root mean square deviation (RMSD), root mean square fluctuation (RMSF), radius of gyration (Rg), number of hydrogen bonds, and principal component analysis (PCA) were calculated by *gmx rms*, *gmx rmsf*, *gmx gyration*, *gmx hbond*, *gmx covar*, *and gmx anaeig* modules, respectively. The Gibbs free energy landscape was generated using the GROMACS *gmx sham* tool as described earlier (Rajendran et al., 2018). The trajectories were rendered using the Chimera 1.13.2.

### 2.8 Binding free energy analysis

The binding free energy analysis describes how the ligand is stable in the protein-binding site through various interactions. We used the g_mmpbsa tool (Kumari et al., 2014) for calculating the binding free energy of the protein–ligand complex. It is based on the molecular mechanics Poisson–Boltzmann surface area (MMPBSA) approach. The last 5-ns trajectory was used for the binding energy calculation. $\Delta G_{bind}$ was calculated by the following Eq. 1:

$$\Delta G_{bind}=\Delta G_{mm}+\Delta G_{sol}-T\Delta S.$$ (1)

$\Delta G_{mm}$ (molecular mechanics energy) is calculated by the van der Waals and electrostatic interactions. Polar and non-polar interactions contributed to the calculation of the solvation-free energy $\left(\Delta G_{sol}\right)$ . The solvent-accessible surface area (SASA) was used for the determination of the non-polar solvation-free energy. The entropy contribution $\left(-T\Delta S\right)$ was excluded because of its high computational cost.

## 3 Results and discussion

### 3.1 Docking protocol validation

**FIGURE 2:**
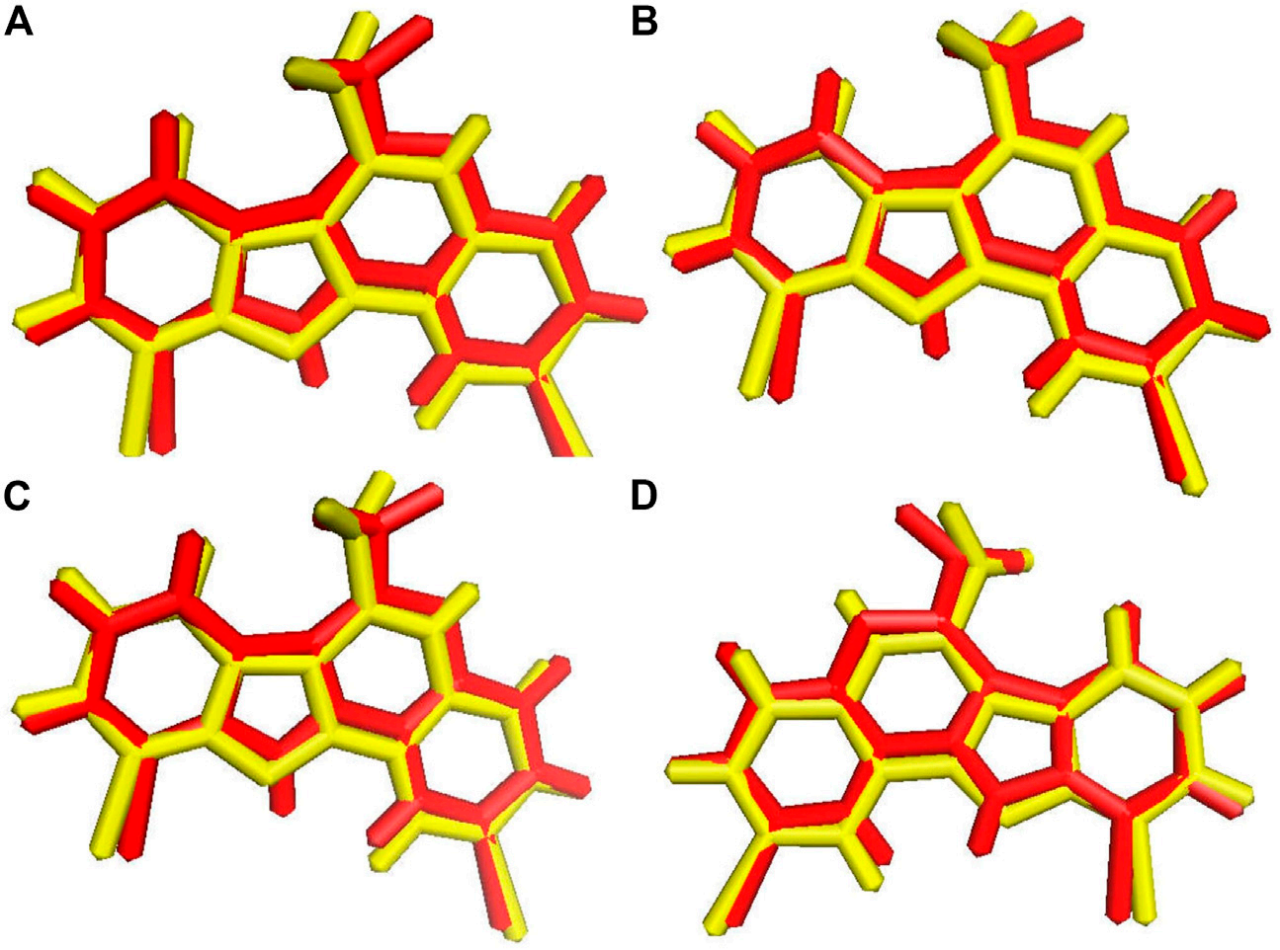
Ligand superimposition. **(A)** AutoDock. **(B)** AutoDock Vina. **(C)** Smina. **(D)** idock. The red and yellow colors represent the co-crystallized and docked ligands, respectively.

Before the virtual screening, we performed docking protocol validation by re-docking analysis. The positive control ligand 4E3, previously co-crystalized with the solved structure of DYRK1A (Falke et al., 2015), was docked against DYRK1A (PDB ID: 4YLL) by all four docking programs. The binding affinity of the positive control 4E3 from AutoDock, AutoDock Vina, Smina, and idock was −9.56, −9.9, −9.9, and −10.11 kcal/mol, respectively. The RMSD between the docked and control ligands was 1.071, 1.070, 1.071, and 1.066 Å from AutoDock, AutoDock Vina, Smina, and idock, respectively (Figure 2). All four software programs produced a binding pose similar to that of the crystal structure. This result represents that these software tools can be used for virtual screening analyses for the library of 98,071 compounds of the ZINC repository against DYRK1A.

### 3.2 Virtual screening

Virtual screening was completed for shortlisting the best compounds from a pool of compounds (*n* = 98,071). All the compounds were prepared and converted into the *pdbqt* file format by using an in-house Python ligand conversion script. Next, the compounds were employed for the virtual screening using all three virtual screening software programs. The top compound ZINC38167083 showed the highest binding free energy from all three software. It showed −14.1, −14.1, and −14.28 kcal/mol from AutoDock Vina, Smina, and idock, respectively. The binding affinity of 98,071 compounds from all three software programs is shown in Supplementary Table S1. We generated a short list of 330 compounds showing a binding energy ≤ −11.5 kcal/mol for pharmacokinetics analysis.

### 3.3 Pharmacokinetics analysis

Pharmacokinetics analysis is a key parameter for drug identification and characterization. Hence, we determined pharmacokinetics analyses using the admetSAR server for all the 330 compounds. We predicted 17 parameters from the admetSAR server, such as the blood–brain barrier, human intestinal absorption, Caco-2 cell permeability, P-gp substrate/inhibitor, cytochrome P450 enzyme inhibition, toxicity, carcinogenicity, and lethal dose (Supplementary Tables S2–S4). We found 14 compounds that showed good activity for all of the 17 parameters except the Caco-2 cell permeability.

### 3.4 Docking analysis

**FIGURE 3:**
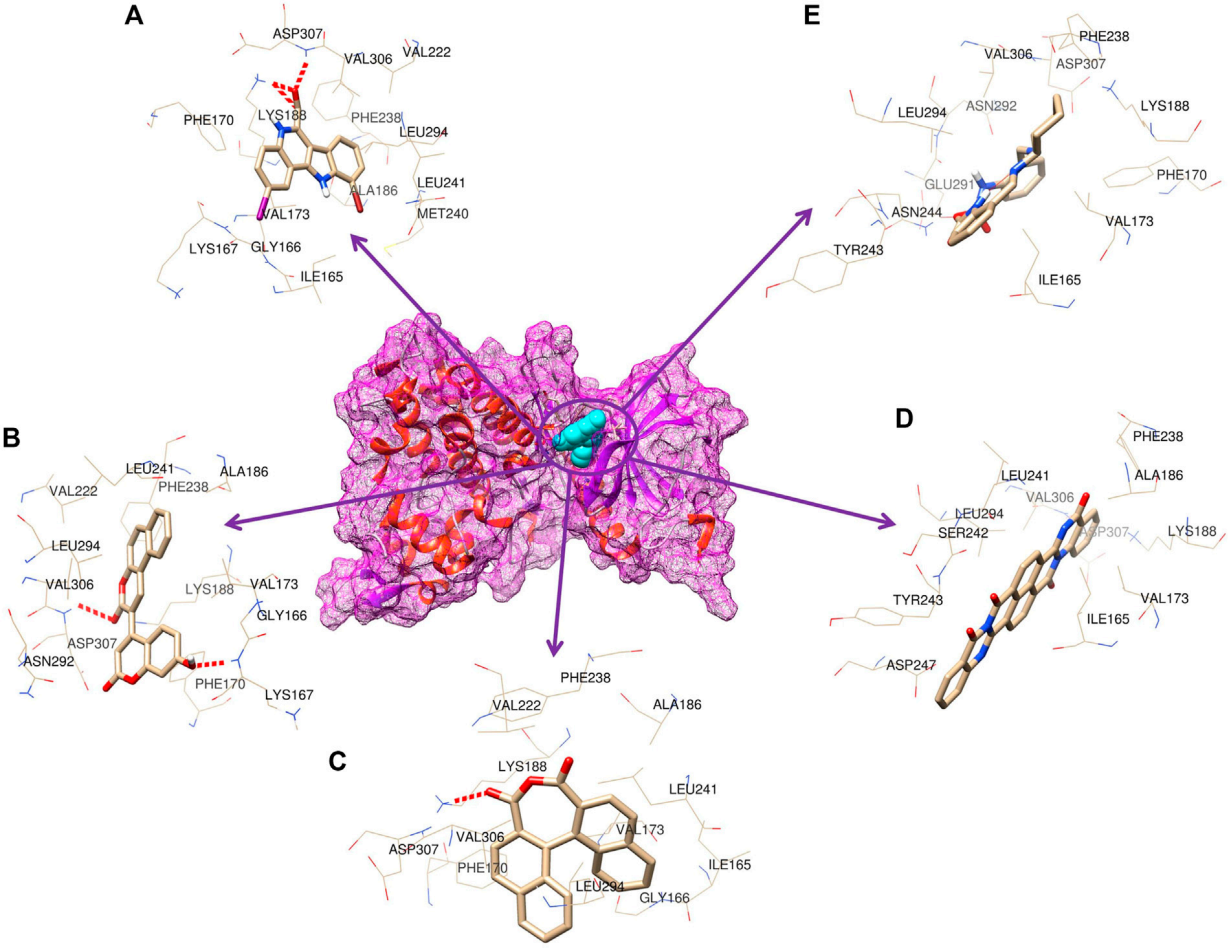
3D representation of the molecular interaction between DYRK1A and ligands. **(A)** 4E3 control, **(B)** ZINC3843365, **(C)** ZINC2123081, **(D)** ZINC5220992, and **(E)** ZINC68569602.

**FIGURE 4:**
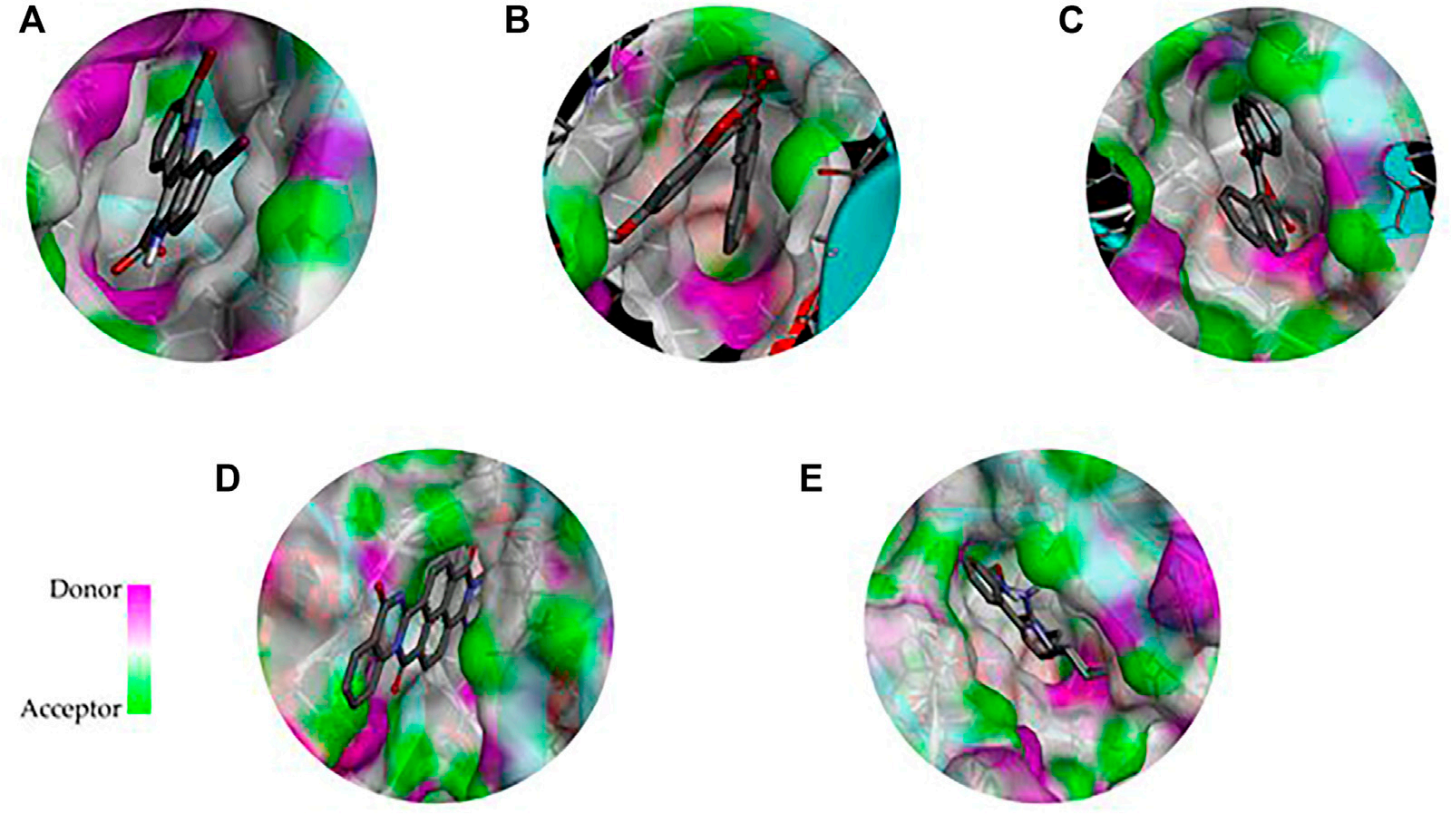
3D representation of the binding cavity of the control and top-ranked molecules to the pocket site of DYRK1A. Pink and green colors reflect the hydrogen bond donor and acceptor, respectively; **(A)** 4E3 control, **(B)** ZINC3843365, **(C)** ZINC2123081, **(D)** ZINC5220992, and **(E)** ZINC68569602.

As many as 14 compounds have been shortlisted for cross docking analysis using the AutoDock program based on the drug likeness evaluation (Supplementary Table S5). Molecular docking revealed that out of 14 selected compounds, four molecules, namely, ZINC3843365, ZINC2123081, ZINC5220992, and ZINC68569602, were found to have the highest negative binding energy of −11.07, −10.93, −10.63, and −10.35 kcal/mol^−1^, respectively, compared to the positive control 4E3, whose binding energy was estimated to be −9.56 kcal/mol^−1^. These four molecules ranked at the top for interacting with DYRK1A based on the binding affinity scores, molecular interaction patterns, and compound specificity criteria as previously described. Popular names, chemical structures (2D), binding energy, and molecular interaction information of top-ranked compounds and controls are presented in Table 1. Chimera Tool was employed to illustrate and annotate the 3D molecular interactions of all four predicted docking complexes (Figure 3). Marking of different molecular interactions, viz., hydrophobic interactions, hydrogen bonds, and salt bridges, help us to understand the binding patterns of top-ranked molecules against DYRK1A. We analyzed the selected four ligands using 3D and 2D interaction diagrams. The hydrogen bonds donor and acceptor view of all the ligands in the DYRK1A binding pocket is shown in Figure 4. The detailed interaction diagrams were generated for all the ligands, including the control compound, and are shown in Supplementary Figure S1. The docking results presented in this study may support previous reports on the inhibition mechanism of small chemical molecules against DYRK1A. Lin et al. (2022) demonstrated that two molecules, NSC361880 and NSC361882, have the potential to inhibit the DYRK1A-mediated tau phosphorylation and contribute to stabilizing the tubulin polymerization in a structure-based virtual screening followed by a set of wet-lab experiments. In a recent study, Shahroz et al. (2022) screened the molMall database (https://www.molmall.net/) against DYRK1A and reported the top six molecules based on the binding affinity, namely, −9,539, −11,352, −15,938, −19,037, −21,830, and −21,878. In a recent follow-up study, Chikodili et al., (Chikodili et al., 2022) explored the African Natural Compounds Database and PubChem-derived natural compounds as potential inhibitors against DYRK1A. The virtual screening results exhibited twelve phytochemicals, namely, 3-[6-(3-methyl-but-2-enyl)-1H indolyl]-6-(3-methyl-but-2-enyl)-1H-indole, lanceolatin B, lysicamine, pratorinine, pratorimine, lanceolatin A, lanuginosine, hippacine, (-)-semiglabrin, aegyptinone B, 3′-prenylnaringenin, and 8-C-p-hydroxybenzylluteolin, as new drug candidates against DYRK1A. The molecular interaction patterns of bioactive molecules against DYRK1A reported in previous studies are consistent with the screening results of the present study (Falke et al., 2015).

**TABLE 1:** Details of the four selected compounds with the control compound 4E3. ZINC ID, 2D structure, and binding affinity obtained after molecular docking are shown.

ZINC ID	Structure	AutoDock (Kcal.mol^-1^)	AutoDock Vina (Kcal.mol^-1^)	Smina (Kcal.mol^-1^)	idock (Kcal.mol^-1^)
4E3	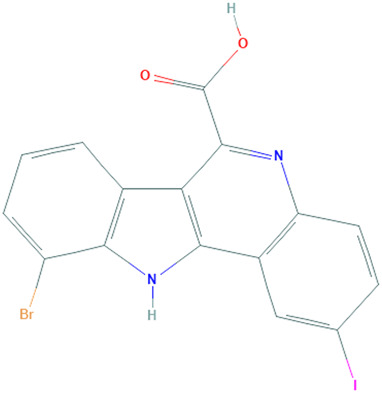	−9.56	−9.9	−9.9	−10.11
ZINC2123081	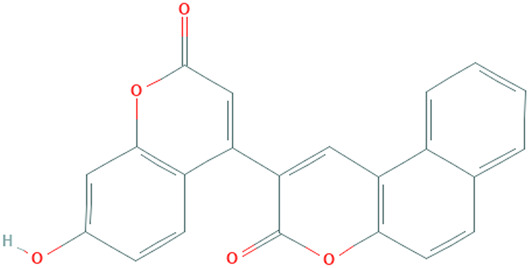	−10.93	−12.1	−12.5	−12.32
ZINC3843365	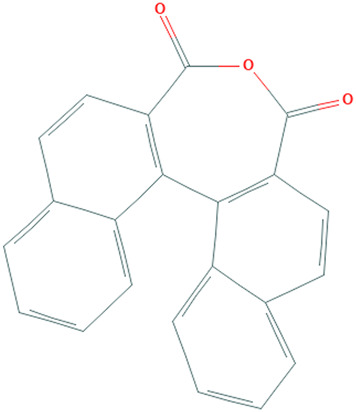	−11.07	−12.5	−12.9	−13.09
ZINC5220992	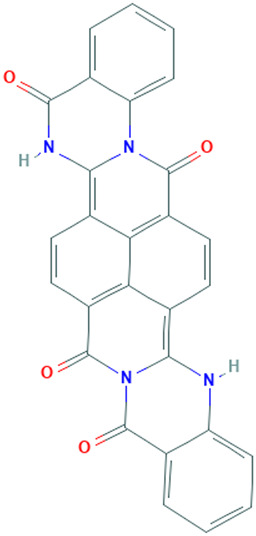	−10.63	−13.3	−13.3	−13.50
ZINC68569602	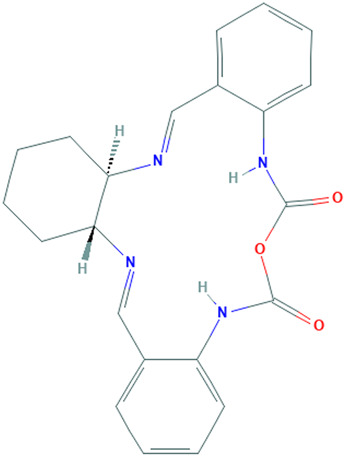	−10.35	−12.3	−12.3	−12.49

### 3.5 Density functional theory analysis

**FIGURE 5:**
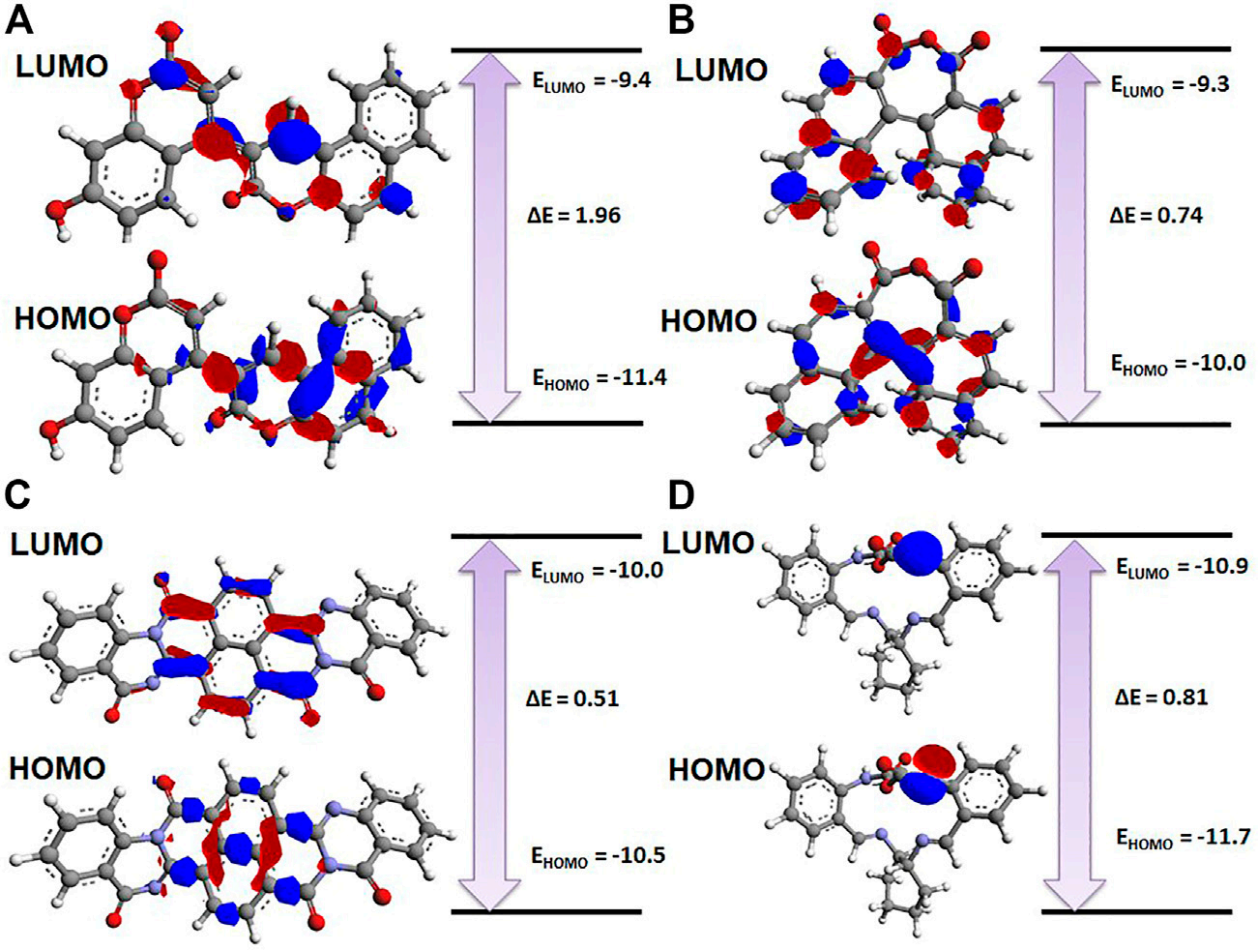
Charge distribution of HOMO and LUMO with their energy bands. **(A)** ZINC2123081, **(B)** ZINC3843365, **(C)** ZINC5220992, and **(D)** ZINC68569602. The red and blue colors represent the negative and positive charge, respectively.

The electronic properties of the selected four hits were analyzed using DFT analysis. The chemical stability of the selected hits was studied by calculating the energy gap between the E_HOMO_ and E_LUMO_ (Johnson et al., 2010). HOMO and LUMO orbitals play a key role in charge transfer between orbitals during a chemical reaction (Ahmed et al., 2021). In previous computer-aided drug-designing studies, the authors have described that higher energy gap compounds cannot quickly donate their electrons, while lower energy gap compounds can quickly donate their electrons and participate in the interaction (Sakkiah and Lee, 2012; Gogoi et al., 2016; Tripathy et al., 2019). E_HOMO_ and E_LUMO_ values and their energy gap were calculated and are shown in Table 2. The energy gap ranged between .512 and 1.959 eV for the selected four compounds. We observed the highest energy gap for ZINC2123081, which indicates that this compound cannot quickly donate its electron. ZINC2123081 requires a greater amount of energy to donate the electron to the LUMO orbital of the binding site amino acid. ZINC5220992 showed the lowest energy gap of .512 eV, which represents that it is a highly reactive and unstable compound. ZINC2123081 can easily donate the electron and participate in the different types of bonding interactions that form through electron donation. ZINC3843365 and ZINC68569602 also showed a lower energy band compared to ZINC2123081. The HOMO and LUMO distribution of the four identified hit molecules (ZINC2123081, ZINC3843365, ZINC5220992, and ZINC68569602) are depicted in Figure 5. Blue and red represent the HOMO and LUMO distribution, respectively, which signify the possible active sites present in the hit molecules (Figure 5).

**TABLE 2:** Orbital energy values of lead compounds with their energy band.

**Name**	**HOMO Energy (kcal/mol)**	**LUMO Energy (kcal/mol)**	**LUMO-HOMO (∆E)**
ZINC2123081	−11.405	−9.446	1.959
ZINC3843365	−10.024	−9.283	0.741
ZINC5220992	−10.513	−10.001	0.512
ZINC68569602	−11.682	−10.872	0.810

### 3.6 Molecular dynamics simulation

The MD simulation was performed to predict the conformational changes after ligand binding. It also explores the stability of the predicted protein–ligand complex. We conducted 100 ns MDS analysis. We initially calculated the RMSD, which showed that after 20 ns, all the systems reached an equilibration state; therefore, we considered the last 80 ns trajectory for further analysis, including RMSF, Rg, SASA, PCA, and binding free energy calculations.

#### 3.6.1 Conformational stability

**FIGURE 6:**
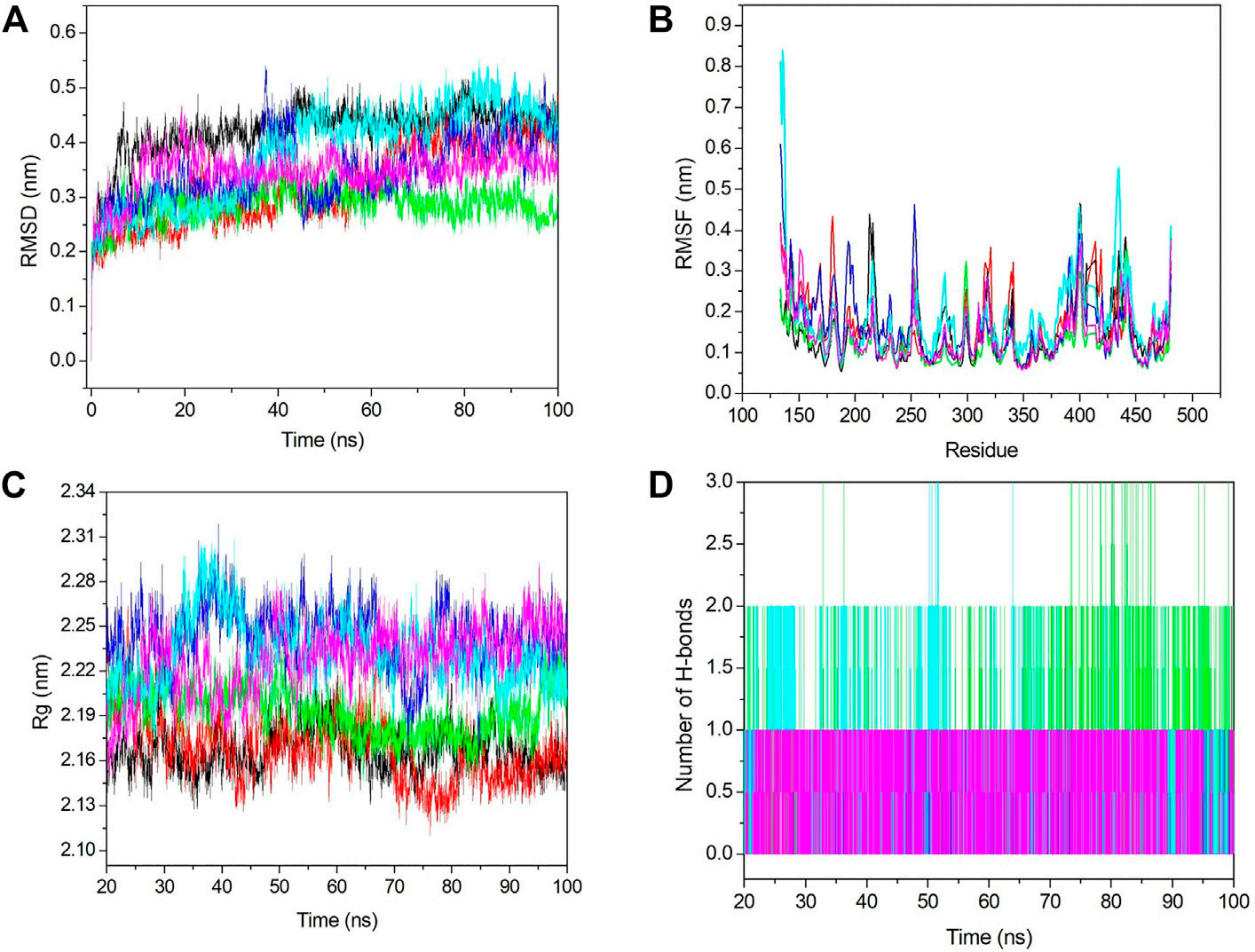
Stability analyses of all the systems. **(A)** RMSD value for 100 ns at 300 K. **(B)** RMSF value from the last 80 ns trajectory. **(C)** Radius of gyration *vs*. the time for the last 80 ns. **(D)** Number of hydrogen bonds with respect to the time for all the selected hits including the control ligand. The black, red, green, blue, cyan, and pink colors represent apo–DYRK1A, DYRK1A–4E3, DYRK1A–ZINC2123081, DYRK1A–ZINC3843365, DYRK1A–ZINC5220992, and DYRK1A–ZINC68569602, respectively.

Conformational stability was analyzed using RMSD analysis. RMSD indicates the deviation between the first and other frames generated during MD simulations. We selected the backbone for the RMSD calculation, and it is shown in Figure 6A. From Figure 6A, we see that initially, RMSD for all the systems was increased, and after 20 ns, it became stabilized. The average RMSD value for apo–DYRK1A, DYRK1A–4E3, DYRK1A–ZINC2123081, DYRK1A–ZINC3843365, DYRK1A–ZINC5220992, and DYRK1A–ZINC68569602 was .42, .32, .28, .34, .38, and .34 nm, respectively. The average value and Figure 6A represent that DYRK1A-ZINC2123081 showed the least RMSD value as compared to apo-DYRK1A and other ligand complexes, including the control compound 4E3. We have also seen that apo-DYRK1A showed .42 nm, which is the highest RMSD value, while after ligand binding, the RMSD value decreased and represents the stability in the protein–ligand complex. The DYRK1A–ZINC5220992 complex showed .38 nm, which was the highest value as compared to the other three predicted hits. The apo–DYRK1A complex showed the highest average value, while the trajectory increased to 8 ns, but after that, it showed a constant peak until the end of the simulation (Figure 6A **)**. The DYRK1A–4E3 complex also showed an increase and decrease in the peak until 60 ns, but after that, it remained stable. The DYRK1A–ZINC3843365 complex also showed a very abrupt type of pattern from 35 to 44 ns. The DYRK1A–ZINC5220992 complex showed a constantly increasing RMSD until 40 ns, where the constant peak remained. The RMSD results indicated that all the systems were stable and could be used for further analysis.

#### 3.6.2 Fluctuation analysis

Fluctuation analysis was performed using RMSF analysis of each complex. RMSF represents the residual fluctuation in a protein. RMSF analysis can track which residue or motif is more flexible or rigid during the simulation. The loosely organized structural components such as turns, loops, and coils generally show higher RMSF values, while the well-folded structures such as alpha-helix and beta-sheets show a lower RMSF. We calculated the RMSF value for all the systems and plotted it in Figure 6B. Figure 6B shows strong fluctuations after ligand binding. The average RMSF values for apo–DYRK1A, DYRK1A–4E3, DYRK1A–ZINC2123081, DYRK1A–ZINC3843365, DYRK1A–ZINC5220992, and DYRK1A–ZINC68569602 were .14, .16, .12, .16, .18, and .14 nm, respectively. The highest RMSF value was observed for DYRK1A–ZINC5220992, which characterizes higher fluctuation and less stability of the complex. The DYRK1A–ZINC5220992 showed a very high fluctuation (.8 nm) in the N-terminal region, but it showed less RMSF values than all the other complexes. The lowest RMSF value (.12 nm) was shown by DYRK1A–ZINC2123081, which represents a stable complex. The DYRK1A–4E3 complex also showed a higher RMSF value (0.16 nm), which is higher than those of apo–DYRK1A, DYRK1A–ZINC2123081, and DYRK1A–ZINC68569602. These results indicate that these two complexes (DYRK1A–ZINC2123081 and DYRK1A–ZINC68569602) are more stable than the DYRK1A–4E3 complex. The DYRK1A–ZINC3843365 complex showed a RMSF value similar to that of the DYRK1A–4E3 complex. We have seen the higher RMSF value from residues 404–418 for DYRK1A–4E3 and DYRK1A–ZINC5220992. The overall RMSF results indicate that DYRK1A–ZINC2123081 and DYRK1A–ZINC68569602 are more stable complexes than the other predicted hits.

#### 3.6.3 Compactness analysis

Compactness analysis was conducted using the Rg calculation. We calculated the Rg value for the last 80 ns trajectory (Figure 6C). The average Rg values for apo–DYRK1A, DYRK1A–4E3, DYRK1A–ZINC2123081, DYRK1A–ZINC3843365, DYRK1A–ZINC5220992, and DYRK1A–ZINC68569602 were 2.16, 2.16, 2.19, 2.43, 2.23, and 2.22 nm, respectively. The average value for DYRK1A–ZINC3843365 showed the highest Rg value when compared to all other systems. All four predicted hits showed a higher Rg value than DYRK1A–4E3 and apo–DYRK1A complexes. This result indicates that DYRK1A–4E3 is more stable than the selected hit complexes. The DYRK1A–ZINC2123081 complex showed the least Rg value as compared to the other selected hits. The DYRK1A–ZINC3843365 and DYRK1A–ZINC5220992 complexes showed a great deal of fluctuation in the peak, which indicates that these complexes are not very compact and ligand binding is inducing conformation changes in the complex. The remaining ligands showed a stable peak. Hence, these results suggest DYRK1A–ZINC2123081 and DYRK1A–ZINC68569602 ranked highest in gyration analysis.

#### 3.6.4 Interaction analysis

Hydrogen bonds are very important and transient interactions for protein–ligand analysis. Here, we have calculated the number of hydrogen bonds for the control ligand 4E3 and predicted hits and plotted them in Figure 6D. DYRK1A–ZINC2123081 showed more hydrogen bonds than 4E3 and other predicted hit complexes. From 73 to 87 ns, DYRK1A–ZINC2123081 also showed 2–3 hydrogen bonds. The average number of hydrogen bonds for all the protein–ligand complexes was 1–2. DYRK1A–4E3 showed only 0–1 hydrogen bonds throughout the simulation, and at the maximum time, it showed no hydrogen bonds. The DYRK1A–ZINC5220992 complex showed 1–2 hydrogen bonds. The DYRK1A–ZINC3843365 complex was stabilized by one hydrogen bond throughout the simulation. DYRK1A–ZINC68569602 also showed one hydrogen bond throughout the simulation. The overall result of the hydrogen bond analysis indicates that all the predicted hits are more stable than the DYRK1A–4E3 complex in terms of hydrogen bonding.

#### 3.6.5 Solvent accessible surface area

**FIGURE 7:**
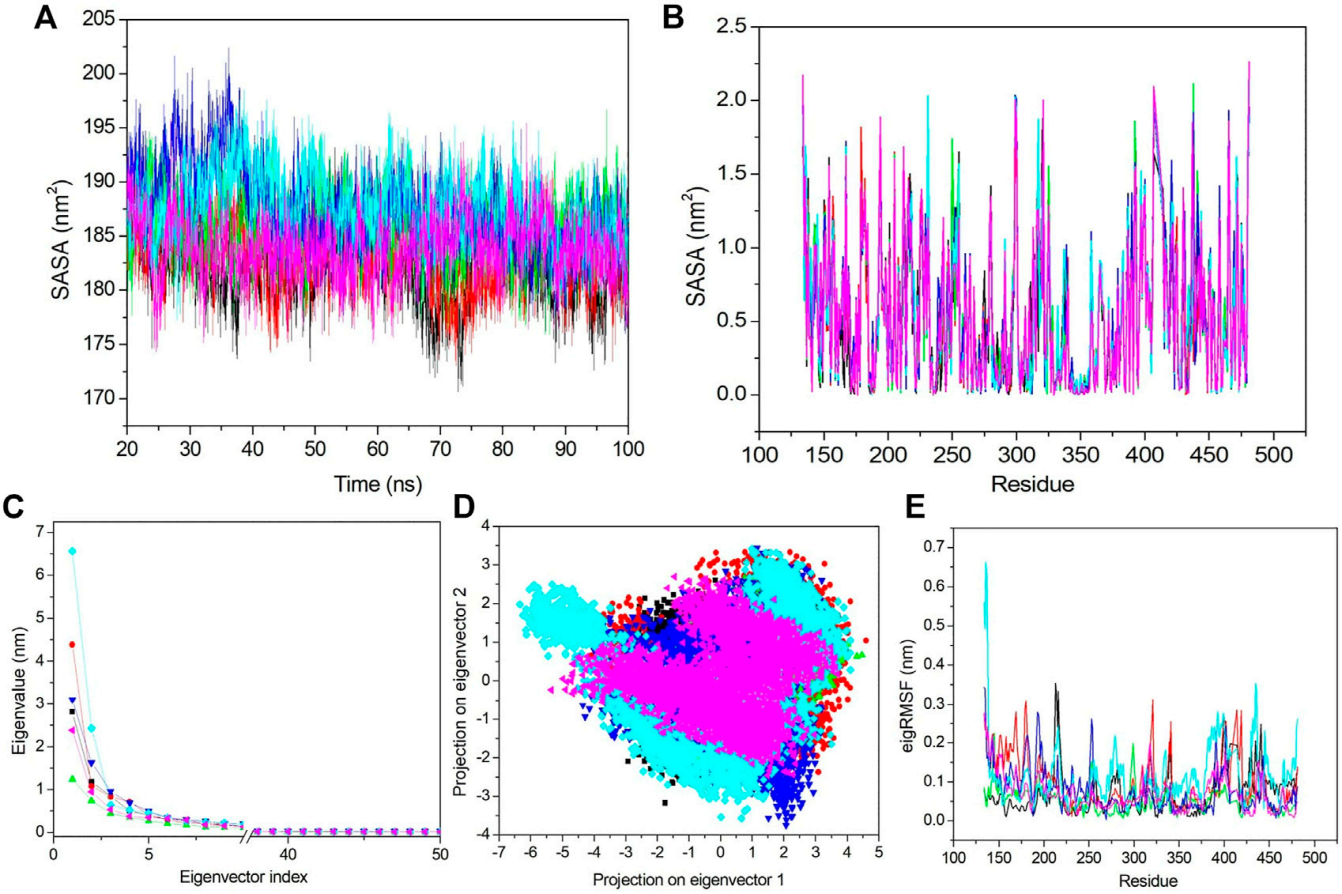
SASA and PCA. **(A)** SASA value *vs*. the time for all the systems. **(B)** SASA value based on residues. **(C)** First 50 PCs *vs*. the eigenvalue for all the systems. **(D)** 2D projection of the first two principal components on the phase space. **(E)** Obtained eigRMSF from the first eigenvector *vs*. the residues. All the calculations have been carried out from the last 80 ns trajectory at 300 K. The black, red, green, blue, cyan, and pink colors represent apo–DYRK1A, DYRK1A–4E3, DYRK1A–ZINC2123081, DYRK1A–ZINC3843365, DYRK1A–ZINC5220992, and DYRK1A–ZINC68569602, respectively.

The solvent-accessible surface area describes the area which can occupy the solvent, and an increased SASA value denotes a less stable complex and *vice versa*. Hence, we calculated the SASA value for all complexes (Figure 7A). The average SASA values for apo–DYRK1A, DYRK1A–4E3, DYRK1A–ZINC2123081, DYRK1A–ZINC3843365, DYRK1A–ZINC5220992, and DYRK1A–ZINC68569602 were 182.45, 182.97, 185.50, 187.34, 187.49, and 183.99 nm^2^. Apo–DYRK1A and DYRK1A–4E3 showed the lowest SASA values compared to those of the other predicted hits. The average SASA value indicates that DYRK1A–ZINC5220992 showed a higher SASA value than SASA values for the other selected hits. The DYRK1A–ZINC3843365 complex showed a slightly lower SASA value than the DYRK1A–ZINC5220992 complex but a higher value than those of DYRK1A–ZINC2123081 and DYRK1A–ZINC68569602 complexes. Figure 7A shows that we also observed a stable type pattern after 20 ns. We have not found any predicted compounds showing a low SASA value compared to that of the control ligand, while the comparison between the predicted hits showed that DYRK1A–ZINC2123081 and DYRK1A–ZINC68569602 are the most stable complexes as compared to the other two predicted hits.

Next, we analyzed the SASA value fluctuation based on residue; hence, the residual SASA changes were calculated and plotted (Figure 7B). The average residue SASA values for apo–DYRK1A, DYRK1A–4E3, DYRK1A–ZINC2123081, DYRK1A–ZINC3843365, DYRK1A–ZINC5220992, and DYRK1A–ZINC68569602 were .53, .53, .54, .54, .54, and .53 nm^2^, respectively. We have seen similar average values for apo–DYRK1A, DYRK1A–4E3, and DYRK1A–ZINC68569602, while DYRK1A–ZINC2123081, DYRK1A–ZINC3843365, and DYRK1A–ZINC5220992 complexes showed higher values than that of the control compound. The overall pattern of SASA values show that all complexes are stable and do not induce substantial residue fluctuation after binding.

#### 3.6.6 Essential dynamics

The essential dynamics (ED) or PCA is used for the calculation of correlated motions after ligand binding. We selected only the first 50 principal components (PCs) and plotted them against the eigenvalue for the clear demonstration of the PCA results (Figure 7C). DYRK1A–ZINC5220992 showed a higher value than the values of other selected hits and control ligand 4E3. Apo–DYRK1A, DYRK1A–ZINC2123081, DYRK1A–ZINC3843365, and DYRK1A–ZINC68569602 showed lower values than that of the DYRK1A–4E3 complex. These results indicate that these three hits are more stable than the control complex, DYRK1A–4E3. We next selected the first 10 eigenvectors and calculated their percent correlation motions. The top 10 eigenvectors contributed with 80.9, 83.66, 71.42, 80.15, 85.5, and 77.8% values for apo–DYRK1A, DYRK1A–4E3, DYRK1A–ZINC2123081, DYRK1A–ZINC3843365, DYRK1A–ZINC5220992, and DYRK1A–ZINC68569602, respectively. This result also indicates higher motions for DYRK1A–ZINC5220992 as compared to 4E3 and other ligands. DYRK1A–ZINC2123081 had the lowest motion, indicating a very stable complex compared to the other predicted hits. DYRK1A–ZINC68569602 also showed a lower value compared to those of DYRK1A–ZINC3843365 and DYRK1A–ZINC5220992. In the comparison of these two predicted hits, DYRK1A–ZINC5220992 showed a higher value than DYRK1A–ZINC3843365. The overall result of PCA suggests that DYRK1A–ZINC2123081 and DYRK1A–ZINC68569602 are the most stable complexes.

From the aforementioned PCA results, we see that the first few eigenvectors are important for characterizing the system dynamics. Hence, we have selected the first two eigenvectors and plotted them against each other in the phase space (Figure 7D). Here, the well-defined and less space-occupying cluster represents the stable complex, while the more space-occupying cluster defines the unstable complex. We observed that apo–DYRK1A showed a more stable cluster compared to the other complexes. DYRK1A–4E3 did not demonstrate a very compact and dense cluster, while DYRK1A–ZINC2123081 showed a very compact and less space-occupying cluster (Figure 7D). While DYRK1A–ZINC3843365 also does not demonstrate a very stable and dense cluster, it also does not showing a much-dispersed type of cluster (Figure 7D). DYRK1A–ZINC5220992 showed a dispersed type of cluster, while DYRK1A–ZINC68569602 showed a stable cluster. 2D PCA suggests that DYRK1A–ZINC2123081 and DYRK1A–ZINC68569602 are more stable than the DYRK1A–4E3 complex (Figure 7D).

We next selected only the first eigenvector to analyze the ligand-binding effect based on the residue. The value was calculated and plotted against the eigenvalue *vs*. the residue (Figure 7E). The average values for apo–DYRK1A, DYRK1A–4E3, DYRK1A–ZINC2123081, DYRK1A–ZINC3843365, DYRK1A–ZINC5220992, and DYRK1A–ZINC68569602 was .07, .09, .05, .07, .11, and .06 nm, respectively. Here, we also observed that DYRK1A–ZINC2123081 showed the least value compared to the control compound and other predicted hits. DYRK1A–ZINC68569602 also showed a lower value as compared to that of DYRK1A–ZINC3843365. DYRK1A–ZINC68569602 showed the least eigRMSF value. DYRK1A–ZINC5220992 showed the highest eigRMSF as compared to all other complexes, including apo–DYRK1A. These results support the view that DYRK1A–ZINC2123081, DYRK1A–ZINC3843365, and DYRK1A–ZINC68569602 are the most stable complexes in terms of eigRMSF analysis.

#### 3.6.7 Gibbs free energy landscape analysis

**FIGURE 8:**
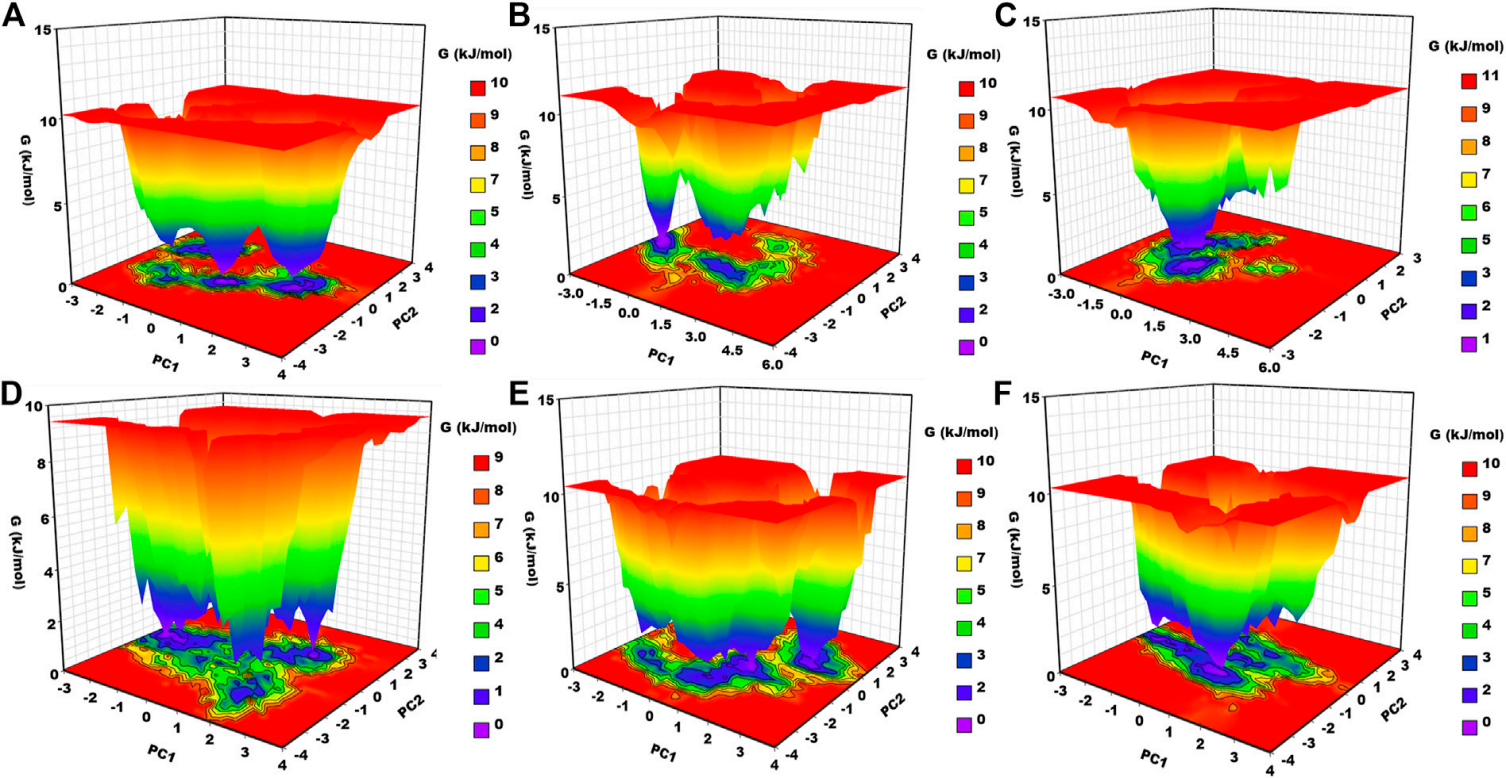
Gibbs free energy landscape. **(A)** apo–DYRK1A, **(B)** DYRK1A–4E3, **(C)** DYRK1A–ZINC2123081, **(D)** DYRK1A–ZINC3843365, **(E)** DYRK1A–ZINC5220992, and **(F)** DYRK1A–ZINC68569602.

Gibbs free energy landscape analysis was conducted using the first two principal components (Figure 8). In Figure 8, the deep blue color represents the lowest energy protein conformation, while the red color represents the highest energy state protein conformation. The deep well represents a thermodynamically favorable state for the proteins. Here, we have calculated the FEL of apo–DYRK1A and its associated protein–ligand complexes. Apo–DYRK1A showed three energy minima, in which one has a bluer color and represents the stable state. It signifies that apo–DYRK1A showed three conformational states. For the DYRK1A–control complex, we have seen two energy funnels, which are separated by an energy barrier, representing that the control complex showed two conformational states. DYRK1A–ZINC2123081 showed clear energy minima with a deep valley, indicating that this complex is much more stable than the control complex. DYRK1A–ZINC3843365 showed many energy funnels, which are very close to each other, signifying that DYRK1A–ZINC3843365 can change its conformational states very quickly and can obtain a new folding state. DYRK1A–ZINC5220992 has a large blue-colored area, which represents the stable cluster. It also showed many energy funnels. We have observed a similar type of pattern for DYRK1A–ZINC68569602. The overall result represents that DYRK1A–ZINC2123081, DYRK1A–ZINC5220992, and DYRK1A–ZINC68569602 are the most stable complexes, and they are the more thermodynamically favorable complexes.

#### 3.6.8 Binding free energy analysis

The binding free energy of all selected hits and the DYRK1A–4E3 complex was calculated by using the MMPBSA tool. The last 5 ns trajectory snapshots were used, and various energetic terms such as the van der Waals energy, electrostatic energy, polar solvation energy, SASA energy, and binding free energy were calculated and shown in Table 3. The binding free energy for DYRK1A–4E3, DYRK1A–ZINC2123081, DYRK1A–ZINC3843365, DYRK1A–ZINC5220992, and DYRK1A–ZINC68569602 was −147.23, −123.19, −120.81, −171.43, and −130.57 kJ mol^−1^, correspondingly. This observation showed that DYRK1A–ZINC5220992 has a higher binding affinity than the control compound 4E3 and other predicted hits, while the control compound 4E3 showed more binding affinity than the other three predicted hits.

**TABLE 3:** Table represents van der Waals, electrostatic, polar solvation, SASA, and binding energies in kJ.mol^−1^ for the control compound (4E3) and predicted hits.

S. No.	Complex	Van der Waals energy	Electrostatic energy	Polar solvation energy	SASA energy	Binding energy
1	DYRK1A-4E3	−210.35 ± 9.24	2.54 ± 5.44	78.73 ± 15.04	−18.15 ± .85	−147.23 ± 15.58
2	DYRK1A-ZINC2123081	−198.86 ± 10.08	−16.69 ± 8.14	109.85 ± 18.95	−17.49 ± .80	−123.19 ± 13.57
3	DYRK1A-ZINC3843365	−160.08 ± 12.84	−12.49 ± 9.07	66.59 ± 15.90	−14.83 ± 1.01	−120.81 ± 13.35
4	DYRK1A-ZINC5220992	−262.79 ± 12.96	−18.54 ± 9.75	130.57 ± 14.73	−20.68 ± .85	−171.43 ± 14.53
5	DYRK1A-ZINC68569602	−175.50 ± 15.92	−4.10 ± 5.06	65.69 ± 14.78	−16.66 ± 1.30	−130.57 ± 14.41

**FIGURE 9:**
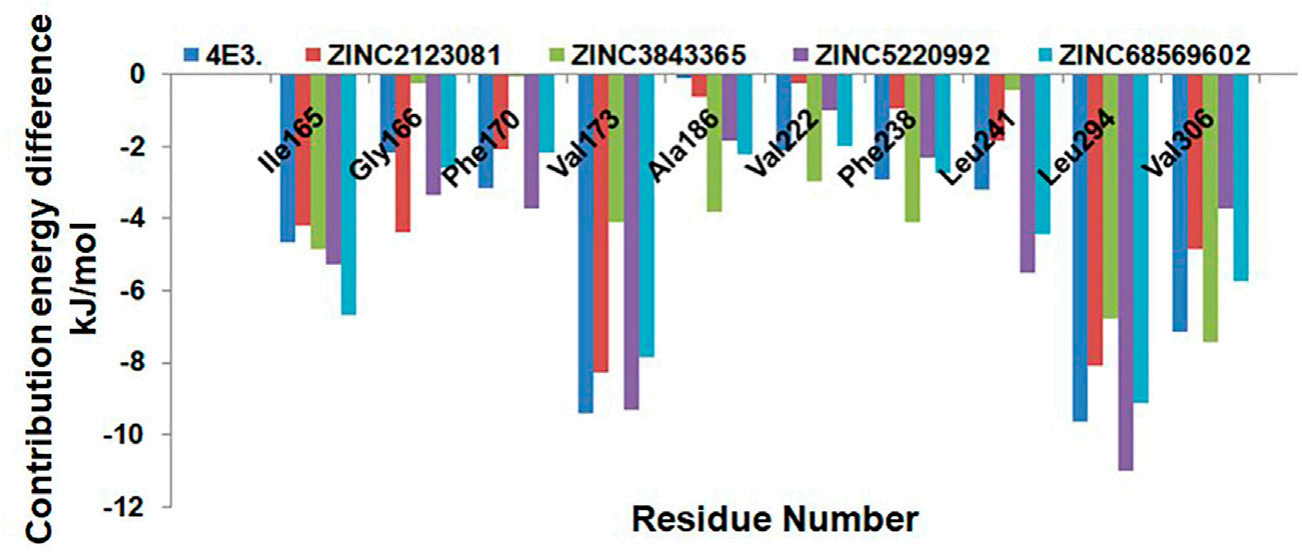
Residue-wise decomposition of the binding free energy. Only the catalytic residues which are actively participating in the ligand stabilization are plotted here.

We next explored the residue-wise energy contribution for all the selected protein–ligand complexes and control compound 4E3. We selected a few important residues to clarify the results and plotted them in Figure 9. We observed that Ile165, Val173, Leu294, and Val306 are the key residues that play important roles in the protein–ligand stabilization. The other residues, Gly166, Phe170, Ala186, Val222, Phe238, and Leu241, also show interactions against DYRK1A. The overall result of binding affinity represents that all the predicted hits show good binding affinity and demonstrate the stability of the complex.

#### 3.6.9 MD pose analysis

Lastly, we analyzed the MD pose from 0 ns to 100 ns and generated a 2D interaction diagram for detailed analysis (Supplementary Figure S2). We have seen many new interactions in 100 ns and the ones not available in the 0 ns frame. We observed two hydrogen bonds with Lys55 and Asp174 in the 0 ns snapshot for the DYRK1A–4E3 complex while these are not visible in the 100 ns snapshot because the ligand orientation was completely changed. In the case of 4YLL–ZINC2123081, we observed more Pi–Pi interactions in the 100-ns snapshot as compared to the 0-ns snapshot. In the 0-ns snapshot, only Lys55 showed one Pi bond with one hydrogen bond interaction, while in the 100-ns snapshot Lys55 showed a Pi–Pi interaction. Additionally, Tyr113 showed one more interaction. Several hydrophobic interactions are also increased. 4YLL–ZINC3843365 showed the Pi–Pi interaction with Lys55 in the 100-ns snapshot, while it was not observed at 0 ns. At 0 ns, 4YLL–ZINC5220992 showed a two-hydrogen bond interaction with Leu108, while the Pi–Pi interaction was increased at 100 ns. Gly33 and Phe105 showed the Pi–Pi interaction. Lastly, we analyzed 4YLL–ZINC68569602, and it did not show major changes in the interaction. We also did not see a major loss in the interaction. The overall analysis showed that all the complexes retain 0-ns interactions, and they form more interactions with several other residues. These observations indicate that all ligands are stable in the binding cavity of DYRK1A during a 100-ns simulation.

## 4 Conclusion

AD has been recognized as one of the most prevalent chronic neurodegenerative disease, affecting approximately 44 million people worldwide in a significant manner. The overexpression of DYRK1A plays a principal role in cognitive deficits in people suffering from AD. The screening of leading compounds against significant targets has become a promising strategy in the discovery of potential drugs using an integrated molecular docking and molecular modeling approach. In this study, we performed high-throughput virtual screening, pharmacokinetics analysis, PCA, and MD simulations to identify the best lead molecules among the ZINC library of 98,071 compounds. A virtual screening-based molecular docking pipeline was employed to shortlist the best lead compounds against DYRK1A based on the binding affinity and drug-likeness properties. A total of 14 compounds were shortlisted based on the binding scores followed by the drug likeness property evaluation. Based on the cross-docking analysis, four compounds, viz., ZINC3843365, ZINC2123081, ZINC5220992, and ZINC68569602, were ranked as the top interacting molecules significantly interacting with the pocket site of DYRK1A. Additionally, MD simulation analysis was conducted to investigate the interaction pattern stability of these compounds with DYRK1A at the atomic level on 100 ns. Binding free energy analysis of docked complexes supported the MD simulation trajectories and confirmed the stability of top-ranked molecules. The outcome of the present study suggests that the four compounds, namely, ZINC3843365, ZINC2123081, ZINC5220992, and ZINC68569602, may evolve as promising therapeutics against AD and can be further investigated in a wet-lab with the help of cell culture and small animal experiments.

## Data Availability

The original contributions presented in the study are included in the article/Supplementary Material; further inquiries can be directed to the corresponding authors.
