# Supplementary material for: Disruption of DYRK1A-induced hyperphosphorylation of amyloid-beta and tau protein in Alzheimer’s disease: An integrative molecular modeling approach
Source: Front Mol Biosci. 2023 Jan 19;9:1078987. doi: 10.3389/fmolb.2022.1078987 (PMC9892649; doi:10.3389/fmolb.2022.1078987)
Supplement: Supplementary file 1 [file Table1.docx]

**Disruption of DYRK1A induced hyperphosphorylation of amyloid-beta and tau protein in Alzheimer’s disease using small molecules**

*Identification of small molecules against* dual-specificity *tyrosine phosphorylation-regulated kinase-1A*

Rohit Shukla^1,2^, Anuj Kumar^3,4^, David J. Kelvin^3,4^*, Tiratha Raj Singh^1,2^*

^1^Department of Biotechnology and Bioinformatics, Jaypee University of Information Technology (JUIT), Waknaghat, Solan, H.P., 173234, India

^2^Centre for Excellence in Healthcare Technologies and Informatics (CEHTI), Jaypee University of Information Technology (JUIT), Waknaghat, Solan, H.P., 173234, India

^3^Laboratory of Immunity, Shantou University Medical College, Shantou, China

^4^Department of Microbiology and Immunology, Canadian Centre for Vaccinology CCfV, Faculty of Medicine, Dalhousie University, Halifax, Canada

**^*^Correspondence:** David J. Kelvin (David.Kelvin@dal.ca), and Tiratha Raj Singh (tiratharaj.singh@juit.ac.in)

**Keywords:** Alzheimer’s disease, Neurofibrillary tangles, DYRK1A, Virtual Screening, Molecular Docking, Molecular Dynamics Simulation, Principal Component analysis, MM-PBSA.

**Supplementary Table S2.** *in-silico* absorption and distribution profile obtained from admetSAR server for selected 330 compounds from virtual screening. Selected compounds (14) for re-docking were highlighted in bold.

| **Sr. No.** | **ZINC ID** | **BBB probability** | **HIA-probability** | **Caco-2 permeability probability** | **Caco-2 permeability** | | **P-gp substrate** | **P-gp inhibitor** |
| --- | --- | --- | --- | --- | --- | --- | --- | --- |
|  | ZINC00120631 | +/0.9984 | +/1.0000 | +/0.6703 | 1.7872 | | Non-Substrate | Inhibitor |
|  | ZINC00238468 | +/0.8911 | +/0.9962 | +/0.5829 | 1.2643 | | Non-Substrate | Non-Inhibitor |
|  | ZINC00338371 | +/0.9938 | +/0.9938 | +/0.6983 | 1.3739 | | Non-Substrate | Non-Inhibitor |
|  | ZINC00408721 | +/0.9233 | +/0.9700 | -/0.6355 | 0.2858 | | Substrate | Non-Inhibitor |
|  | ZINC00490486 | +/0.9149 | +/1.0000 | +/0.6666 | 1.3599 | | Non-Substrate | Non-Inhibitor |
|  | ZINC00519086 | +/0.8911 | +/0.9962 | +/0.5829 | 1.2643 | | Non-Substrate | Non-Inhibitor |
|  | ZINC01482030 | +/0.9946 | +/0.9249 | +/0.6913 | 1.0890 | | Substrate | Non-Inhibitor |
|  | ZINC01686586 | +/0.9606 | +/1.0000 | +/0.8620 | 1.9213 | | Non-Substrate | Non-Inhibitor |
|  | ZINC01726969 | +/0.9528 | +/1.0000 | +/0.8720 | 1.9084 | | Non-Substrate | Non-Inhibitor |
|  | ZINC01787663 | +/0.8271 | +/0.9533 | -/0.5473 | 0.3556 | | Substrate | Non-Inhibitor |
|  | ZINC01787672 | +/0.8271 | +/0.9533 | -/0.5473 | 0.3556 | | Substrate | Non-Inhibitor |
|  | ZINC01845382 | +/0.9275 | +/0.9927 | -/0.5915 | 0.5670 | | Substrate | Non-Inhibitor |
|  | ZINC01886047 | +/0.8807 | +/0.9961 | -/0.5827 | 0.7619 | | Substrate | Inhibitor |
|  | ZINC02001186 | +/0.7198 | +/0.9829 | -/0.5061 | 0.9924 | | Substrate | Non-Inhibitor |
|  | ZINC02092089 | +/0.9730 | +/1.0000 | -/0.5585 | 0.8765 | | Substrate | Inhibitor |
|  | ZINC02092130 | +/0.9523 | +/1.0000 | +/0.5211 | 1.0707 | | Substrate | Inhibitor |
|  | ZINC02092414 | +/0.8713 | +/0.9948 | -/0.5777 | 0.6481 | | Substrate | Non-Inhibitor |
|  | ZINC02092422 | +/0.9468 | +/1.0000 | +/0.5155 | 1.0699 | | Substrate | Inhibitor |
|  | ZINC02092475 | +/0.9730 | +/1.0000 | -/0.5585 | 0.8765 | | Substrate | Inhibitor |
|  | ZINC02092931 | +/0.8740 | +/0.9337 | -/0.6334 | 0.5796 | | Substrate | Non-Inhibitor |
|  | ZINC02093637 | +/0.9275 | +/0.9927 | -/0.5915 | 0.5670 | | Substrate | Non-Inhibitor |
|  | ZINC02094632 | +/0.9523 | +/1.0000 | +/0.5211 | 1.0707 | | Substrate | Inhibitor |
|  | ZINC02095117 | +/0.9722 | +/1.0000 | +/0.5088 | 0.9775 | | Substrate | Non-Inhibitor |
|  | ZINC02095140 | +/0.8280 | +/0.9896 | -/0.5625 | 0.3630 | | Substrate | Non-Inhibitor |
|  | ZINC02095426 | +/0.9493 | +/0.9907 | -/0.6473 | 0.5569 | | Substrate | Inhibitor |
|  | ZINC02096038 | +/0.9853 | +/1.0000 | -/0.5195 | 0.9349 | | Substrate | Non-Inhibitor |
|  | ZINC02096725 | +/0.9024 | +/0.9954 | -/0.5430 | 0.7422 | | Substrate | Inhibitor |
|  | ZINC02098157 | +/0.9145 | +/0.9962 | -/0.5590 | 0.7049 | | Substrate | Inhibitor |
|  | ZINC02098972 | +/0.9565 | +/1.0000 | -/0.5342 | 0.8920 | | Substrate | Non-Inhibitor |
|  | ZINC02100762 | +/0.8986 | +/1.0000 | +/0.5000 | 1.2356 | | Substrate | Inhibitor |
|  | ZINC02101594 | +/0.9442 | +/1.0000 | +/0.5000 | 1.0551 | | Substrate | Inhibitor |
|  | ZINC02101693 | -/0.8861 | +/0.7633 | -/0.5745 | 0.7949 | | Substrate | Inhibitor |
|  | ZINC02101695 | -/0.8861 | +/0.7633 | -/0.5745 | 0.7949 | | Substrate | Inhibitor |
|  | ZINC02102083 | +/0.8856 | +/0.9958 | -/0.5579 | 1.2787 | | Substrate | Non-Inhibitor |
|  | ZINC02102560 | +/0.9523 | +/1.0000 | +/0.5211 | 1.0707 | | Substrate | Inhibitor |
|  | ZINC02103115 | +/0.9681 | +/1.0000 | +/0.5386 | 0.9178 | | Non-Substrate | Inhibitor |
|  | ZINC02103122 | +/0.9635 | +/1.0000 | +/0.5000 | 1.0066 | | Substrate | Inhibitor |
|  | ZINC02103383 | +/0.9524 | +/1.0000 | +/0.5000 | 0.9965 | | Substrate | Inhibitor |
|  | ZINC02103425 | +/0.9722 | +/1.0000 | +/0.5088 | 0.9775 | | Substrate | Non-Inhibitor |
|  | ZINC02103558 | +/0.9145 | +/0.9962 | -/0.5590 | 0.7049 | | Substrate | Inhibitor |
|  | ZINC02103644 | +/0.8876 | +/0.9960 | -/0.5486 | 0.7564 | | Substrate | Inhibitor |
|  | ZINC02104482 | +/0.8413 | +/0.9923 | -/0.5638 | 0.8228 | | Substrate | Non-Inhibitor |
|  | ZINC02105209 | +/0.8988 | +/0.9959 | -/0.5573 | 0.8057 | | Substrate | Inhibitor |
|  | ZINC02106269 | +/0.9565 | +/1.0000 | -/0.5342 | 0.8920 | | Substrate | Non-Inhibitor |
|  | ZINC02106282 | +/0.9722 | +/1.0000 | +/0.5088 | 0.9775 | | Substrate | Non-Inhibitor |
|  | ZINC02107064 | +/0.8876 | +/0.9960 | -/0.5486 | 0.7564 | | Substrate | Inhibitor |
|  | ZINC02107810 | +/0.8413 | +/0.9923 | -/0.5638 | 0.8228 | | Substrate | Non-Inhibitor |
|  | ZINC02108288 | +/0.9730 | +/1.0000 | -/0.5585 | 0.8765 | | Substrate | Inhibitor |
|  | ZINC02108366 | +/0.9689 | +/1.0000 | -/0.5057 | 0.9447 | | Substrate | Non-Inhibitor |
|  | ZINC02108756 | +/0.9110 | +/0.9949 | +/0.7389 | 0.6812 | | Non-Substrate | Inhibitor |
|  | ZINC02109073 | +/0.9442 | +/1.0000 | +/0.5000 | 1.0551 | | Substrate | Inhibitor |
|  | ZINC02109448 |  |  |  |  | |  |  |
|  | ZINC02109745 | +/0.8413 | +/0.9923 | -/0.5638 | 0.8228 | | Substrate | Non-Inhibitor |
|  | ZINC02111048 | +/0.8932 | +/0.9932 | +/0.7765 | 0.8144 | | Non-Substrate | Inhibitor |
|  | ZINC02111151 | +/0.9442 | +/1.0000 | +/0.5000 | 1.0551 | | Substrate | Inhibitor |
|  | ZINC02111981 | +/0.9818 | +/1.0000 | -/0.5746 | 0.8135 | | Substrate | Non-Inhibitor |
|  | ZINC02112230 | +/0.8918 | +/0.9894 | -/0.5631 | 0.7120 | | Substrate | Inhibitor |
|  | ZINC02112613 | +/0.9534 | +/1.0000 | -/0.5609 | 0.9250 | | Substrate | Inhibitor |
|  | ZINC02113936 | +/0.9663 | +/0.9861 | -/0.5657 | 0.5747 | | Substrate | Non-Inhibitor |
|  | ZINC02115702 | +/0.9714 | +/0.9831 | -/0.5590 | 0.6981 | | Substrate | Non-Inhibitor |
|  | ZINC02117133 | +/0.8713 | +/0.9948 | -/0.5777 | 0.6481 | | Substrate | Non-Inhibitor |
|  | ZINC02117888 | +/0.9652 | +/0.9813 | -/0.5694 | 0.7881 | | Substrate | Non-Inhibitor |
|  | ZINC02118353 | +/0.9563 | +/1.0000 | -/0.5123 | 1.1405 | | Substrate | Inhibitor |
|  | ZINC02118360 | +/0.9824 | +/0.9714 | +/0.6167 | 1.0674 | | Non-Substrate | Non-Inhibitor |
|  | ZINC02118901 | +/0.9927 | +/0.9837 | +/0.6103 | 1.1290 | | Non-Substrate | Non-Inhibitor |
|  | ZINC02120774 | +/0.9846 | +/0.7948 | +/0.5695 | 0.7067 | | Substrate | Inhibitor |
|  | ZINC02122409 | +/0.9824 | +/0.9714 | +/0.6167 | 1.0674 | | Non-Substrate | Non-Inhibitor |
|  | **ZINC02123081** | +/0.8671 | +/0.9776 | +/0.5396 | 0.7177 | | Non-Substrate | Non-Inhibitor |
|  | ZINC02123191 | +/0.9887 | +/0.9935 | +/0.6107 | 1.2622 | | Non-Substrate | Non-Inhibitor |
|  | ZINC02123282 | +/0.9209 | +/0.9957 | +/0.5074 | 1.3701 | | Substrate | Non-Inhibitor |
|  | ZINC02123424 | +/0.9892 | +/0.9958 | +/0.6610 | 1.5587 | | Non-Substrate | Non-Inhibitor |
|  | ZINC02123859 | +/0.9898 | +/0.9962 | +/0.6701 | 1.4651 | | Non-Substrate | Non-Inhibitor |
|  | ZINC02125038 | +/0.9868 | +/0.9735 | +/0.5991 | 1.0872 | | Substrate | Non-Inhibitor |
|  | ZINC02125714 | +/0.9662 | +/1.0000 | +/0.6434 | 1.5687 | | Non-Substrate | Non-Inhibitor |
|  | ZINC02148197 | +/0.7350 | +/0.9903 | -/0.6014 | 0.6330 | | Substrate | Non-Inhibitor |
|  | ZINC02148481 | +/0.8807 | +/0.9890 | -/0.5109 | 1.1844 | | Substrate | Non-Inhibitor |
|  | ZINC02148655 | +/0.9524 | +/1.0000 | +/0.5000 | 0.9965 | | Substrate | Inhibitor |
|  | ZINC02148909 | +/0.9825 | +/1.0000 | -/0.5783 | 0.7928 | | Substrate | Non-Inhibitor |
|  | ZINC02148919 | +/0.8856 | +/0.9958 | -/0.5579 | 1.2787 | | Substrate | Non-Inhibitor |
|  | ZINC02148935 | +/0.9250 | +/0.9911 | -/0.5843 | 0.6037 | | Substrate | Non-Inhibitor |
|  | ZINC02149492 | +/0.8807 | +/0.9961 | -/0.5827 | 0.7619 | | Substrate | Inhibitor |
|  | ZINC02150498 | +/0.7619 | +/0.9829 | -/0.5747 | 0.4325 | | Substrate | Non-Inhibitor |
|  | ZINC02151835 | +/0.7201 | +/0.9827 | -/0.6486 | 0.7162 | | Substrate | Inhibitor |
|  | ZINC02151836 | +/0.7201 | +/0.9827 | -/0.6486 | 0.7162 | | Substrate | Inhibitor |
|  | ZINC02154469 | +/0.9442 | +/1.0000 | +/0.5000 | 1.0551 | | Substrate | Inhibitor |
|  | ZINC02154866 | +/0.9689 | +/1.0000 | -/0.5057 | 0.9447 | | Substrate | Non-Inhibitor |
|  | ZINC02155992 | +/0.9468 | +/1.0000 | +/0.5155 | 1.0699 | | Substrate | Inhibitor |
|  | ZINC02156445 | +/0.9306 | +/1.0000 | +/0.5735 | 1.3182 | | Substrate | Non-Inhibitor |
|  | ZINC02156984 | +/0.9818 | +/1.0000 | -/0.5746 | 0.8135 | | Substrate | Non-Inhibitor |
|  | ZINC02156988 | +/0.9818 | +/1.0000 | -/0.5746 | 0.8135 | | Substrate | Non-Inhibitor |
|  | ZINC02157224 | +/0.8303 | +/0.9907 | +/0.8306 | 0.8549 | | Non-Substrate | Inhibitor |
|  | ZINC02158043 | +/0.9468 | +/1.0000 | +/0.5155 | 1.0699 | | Substrate | Inhibitor |
|  | ZINC02158790 | -/0.7824 | +/0.6130 | -/0.5469 | 0.1461 | | Substrate | Non-Inhibitor |
|  | ZINC02159274 | +/0.9635 | +/1.0000 | +/0.5000 | 1.0066 | | Substrate | Inhibitor |
|  | ZINC02159526 | +/0.9858 | +/0.9972 | +/0.6685 | 1.5813 | | Non-Substrate | Non-Inhibitor |
|  | ZINC02159783 | +/0.9753 | +/0.9952 | +/0.6699 | 1.6306 | | Non-Substrate | Non-Inhibitor |
|  | ZINC02160471 | +/0.9892 | +/0.9958 | +/0.6610 | 1.5585 | | Non-Substrate | Non-Inhibitor |
|  | ZINC02503427 | +/0.9899 | +/0.9805 | +/0.6785 | 1.3719 | | Non-Substrate | Inhibitor |
|  | ZINC03842059 | +/0.9425 | +/0.8350 | -/0.6015 | 0.5698 | | Substrate | Inhibitor |
|  | ZINC03843035 | +/0.9902 | +/1.0000 | +/0.6630 | 1.5994 | | Non-Substrate | Inhibitor |
|  | **ZINC03843365** | +/0.9600 | +/0.9888 | +/0.7857 | 1.3284 | | Non-Substrate | Non-Inhibitor |
|  | ZINC03844760 | +/0.9908 | +/0.9879 | +/0.7955 | 1.7780 | | Non-Substrate | Non-Inhibitor |
|  | ZINC03845096 | +/0.9704 | +/0.9960 | +/0.6027 | 1.3640 | | Non-Substrate | Non-Inhibitor |
|  | **ZINC03845323** | +/0.9464 | +/0.8083 | -/0.5283 | 0.9140 | | Non-Substrate | Non-Inhibitor |
|  | ZINC03845515 | +/0.9839 | +/1.0000 | +/0.5437 | 1.2741 | | Non-Substrate | Non-Inhibitor |
|  | ZINC03845516 | +/0.9839 | +/1.0000 | +/0.5437 | 1.2741 | | Non-Substrate | Non-Inhibitor |
|  | ZINC03846581 | +/0.8898 | -/0.5562 | +/0.5975 | 1.0156 | | Non-Substrate | Non-Inhibitor |
|  | ZINC03846582 | +/0.8898 | -/0.5562 | +/0.5975 | 1.0156 | | Non-Substrate | Non-Inhibitor |
|  | ZINC03846898 | +/0.8055 | +/0.9742 | +/0.5932 | 1.0040 | | Non-Substrate | Inhibitor |
|  | ZINC03847083 | +/0.9631 | +/0.9954 | +/0.6398 | 1.2856 | | Substrate | Non-Inhibitor |
|  | ZINC03847184 | -/0.5487 | -/0.6868 | +/0.5191 | 0.6104 | | Substrate | Non-Inhibitor |
|  | ZINC03848443 | +/0.9382 | +/1.0000 | +/0.5171 | 1.4410 | | Non-Substrate | Inhibitor |
|  | ZINC03848917 | +/0.9275 | +/0.9941 | -/0.5406 | 1.1445 | | Non-Substrate | Inhibitor |
|  | **ZINC03849421** | +/0.9740 | +/0.9419 | -/0.5476 | 0.8469 | | Non-Substrate | Non-Inhibitor |
|  | ZINC03851871 | +/0.9484 | +/0.9363 | +/0.5478 | 0.8708 | | Substrate | Non-Inhibitor |
|  | ZINC03872986 | -/0.8608 | +/0.5666 | -/0.6334 | 0.6267 | | Substrate | Non-Inhibitor |
|  | ZINC03927200 | +/0.9383 | +/1.0000 | +/0.6376 | 1.3362 | | Substrate | Non-Inhibitor |
|  | ZINC03960761 | +/0.9048 | +/0.8352 | +/0.5456 | 1.0010 | | Non-Substrate | Non-Inhibitor |
|  | ZINC03979002 | +/0.7096 | +/0.9681 | -/0.6222 | 0.6980 | | Substrate | Non-Inhibitor |
|  | ZINC04017533 | +/0.5590 | +/0.9861 | +/0.7775 | 1.1183 | | Substrate | Non-Inhibitor |
|  | ZINC04026127 | +/0.9554 | +/1.0000 | +/0.6666 | 1.2198 | | Substrate | Non-Inhibitor |
|  | ZINC04026666 | +/0.9739 | +/1.0000 | +/0.6297 | 1.2711 | | Substrate | Non-Inhibitor |
|  | ZINC04027261 | +/0.9176 | +/0.9886 | +/0.5000 | 0.9038 | | Substrate | Inhibitor |
|  | ZINC04046706 | +/0.9595 | +/0.9957 | -/0.5269 | 0.8378 | | Substrate | Non-Inhibitor |
|  | ZINC04062050 | +/0.9667 | +/0.9921 | +/0.5000 | 0.8298 | | Substrate | Non-Inhibitor |
|  | ZINC04073740 | +/0.8517 | +/0.7661 | -/0.5513 | 0.6645 | | Substrate | Non-Inhibitor |
|  | ZINC04084783 | +/0.9551 | +/0.9514 | +/0.5636 | 0.8828 | | Substrate | Non-Inhibitor |
|  | ZINC04084832 | +/0.9943 | +/0.9965 | +/0.5482 | 1.2510 | | Non-Substrate | Non-Inhibitor |
|  | ZINC04084888 | +/0.9834 | +/0.9924 | +/0.5399 | 1.4635 | | Non-Substrate | Non-Inhibitor |
|  | ZINC04084890 | +/0.9834 | +/0.9924 | +/0.5399 | 1.4635 | | Non-Substrate | Non-Inhibitor |
|  | ZINC04085127 | +/0.9905 | +/0.9954 | +/0.6427 | 1.5777 | | Non-Substrate | Non-Inhibitor |
|  | ZINC04085162 | +/0.9935 | +/1.0000 | +/0.6338 | 1.6138 | | Non-Substrate | Inhibitor |
|  | ZINC04085562 | +/0.9926 | +/1.0000 | +/0.6675 | 1.6747 | | Non-Substrate | Non-Inhibitor |
|  | ZINC04086945 | +/0.9947 | +/0.9958 | +/0.6916 | 1.4392 | | Non-Substrate | Non-Inhibitor |
|  | **ZINC04090179** | +/0.8050 | +/0.9753 | +/0.6113 | 1.3036 | | Non-Substrate | Non-Inhibitor |
|  | ZINC04090180 | +/0.8050 | +/0.9753 | +/0.6113 | 1.3036 | | Non-Substrate | Non-Inhibitor |
|  | **ZINC04090428** | +/0.6896 | +/0.9687 | +/0.6029 | 1.3470 | | Non-Substrate | Non-Inhibitor |
|  | ZINC04235972 | +/0.9277 | +/0.9840 | -/0.6559 | 0.5792 | | Substrate | Non-Inhibitor |
|  | ZINC04236421 | +/0.9403 | +/0.9919 | -/0.6635 | 0.8059 | | Substrate | Non-Inhibitor |
|  | ZINC04237100 | +/0.9932 | +/1.0000 | +/0.5362 | 1.4384 | | Non-Substrate | Non-Inhibitor |
|  | ZINC04258893 | +/0.9758 | +/0.9941 | +/0.5524 | 1.1640 | | Substrate | Non-Inhibitor |
|  | ZINC04258903 | +/0.9716 | +/0.9929 | +/0.5429 | 1.1105 | | Substrate | Non-Inhibitor |
|  | ZINC04259264 | +/0.8336 | +/0.8776 | -/0.7258 | 0.5406 | | Substrate | Inhibitor |
|  | ZINC04655273 | +/0.9450 | +/0.9964 | +/0.6403 | 1.0586 | | Substrate | Non-Inhibitor |
|  | **ZINC05220992** | +/0.9867 | +/0.9810 | +/0.5258 | 0.9743 | | Non-Substrate | Non-Inhibitor |
|  | ZINC05397050 | +/0.9951 | +/0.9969 | +/0.5148 | 1.0788 | | Non-Substrate | Inhibitor |
|  | ZINC05415240 | +/0.6513 | +/0.9910 | -/0.6953 | 0.2421 | | Substrate | Non-Inhibitor |
|  | ZINC06092274 | +/0.7150 | +/0.9589 | +/0.5632 | 0.6270 | | Substrate | Non-Inhibitor |
|  | ZINC06137732 | +/0.9625 | +/0.9948 | -/0.5734 | 0.9881 | | Substrate | Non-Inhibitor |
|  | ZINC06624582 | +/0.9703 | +/0.9831 | -/0.5545 | 0.6876 | | Substrate | Non-Inhibitor |
|  | ZINC06624588 | +/0.9915 | +/0.9949 | -/0.5542 | 0.8650 | | Substrate | Non-Inhibitor |
|  | ZINC06624612 | +/0.9941 | +/0.9939 | +/0.5000 | 0.9263 | | Non-Substrate | Non-Inhibitor |
|  | ZINC08299978 | +/0.6535 | +/0.9856 | -/0.5957 | 0.9434 | | Substrate | Non-Inhibitor |
|  | ZINC08382456 | +/0.9933 | +/0.9347 | +/0.5595 | 0.7359 | | Non-Substrate | Inhibitor |
|  | ZINC08445572 | +/0.9900 | +/0.9970 | +/0.5516 | 1.3295 | | Non-Substrate | Inhibitor |
|  | ZINC08649676 | +/0.8030 | +/1.0000 | +/0.5599 | 1.2075 | | Non-Substrate | Non-Inhibitor |
|  | ZINC08739084 | +/0.9363 | +/1.0000 | -/0.5822 | 0.9359 | | Substrate | Inhibitor |
|  | ZINC08764694 | -/0.5419 | +/0.8412 | -/0.6955 | -0.2254 | | Non-Substrate | Inhibitor |
|  | ZINC08765261 | +/0.9847 | +/0.9366 | +/0.6966 | 0.9772 | | Substrate | Inhibitor |
|  | ZINC08790130 | +/0.8366 | +/1.0000 | -/0.6284 | 0.8454 | | Substrate | Inhibitor |
|  | ZINC08791465 | +/0.7294 | +/0.9642 | -/0.6410 | 0.2206 | | Substrate | Non-Inhibitor |
|  | ZINC08791487 | +/0.8647 | +/0.9681 | -/0.6455 | 0.3483 | | Substrate | Non-Inhibitor |
|  | ZINC08791488 | +/0.8647 | +/0.9681 | -/0.6455 | 0.3483 | | Substrate | Non-Inhibitor |
|  | ZINC08791610 | +/0.5911 | +/0.9845 | -/0.6463 | 0.2701 | | Substrate | Non-Inhibitor |
|  | ZINC08792167 | +/0.6560 | +/0.9240 | -/0.5518 | 1.0291 | | Substrate | Non-Inhibitor |
|  | ZINC08792170 | +/0.6608 | +/0.9545 | -/0.6042 | 0.5029 | | Non-Substrate | Inhibitor |
|  | ZINC08792350 | +/0.9931 | +/0.9958 | -/0.6079 | 0.8759 | | Substrate | Inhibitor |
|  | ZINC08792429 | +/0.9352 | +/0.9959 | -/0.5495 | 0.7357 | | Substrate | Inhibitor |
|  | ZINC08792433 | +/0.9319 | +/0.9151 | -/0.5690 | 0.7150 | | Substrate | Inhibitor |
|  | ZINC08877830 | +/0.9744 | +/0.9974 | -/0.5192 | 0.9571 | | Substrate | Inhibitor |
|  | ZINC08877855 | +/0.9744 | +/0.9974 | -/0.5192 | 0.9571 | | Substrate | Inhibitor |
|  | ZINC08877857 | +/0.9497 | +/0.9280 | -/0.5769 | 0.8159 | | Non-Substrate | Non-Inhibitor |
|  | ZINC08878911 | +/0.9547 | +/0.6854 | +/0.5867 | 1.0129 | | Substrate | Non-Inhibitor |
|  | ZINC08878978 | +/0.9361 | +/0.9396 | -/0.5882 | 0.8637 | | Substrate | Non-Inhibitor |
|  | ZINC08918127 | -/0.6020 | +/0.6766 | -/0.7501 | 0.1976 | | Substrate | Non-Inhibitor |
|  | ZINC08918259 | +/0.5111 | -/0.5615 | -/0.7961 | 0.2066 | | Substrate | Non-Inhibitor |
|  | **ZINC08918302** | +/0.9808 | +/0.9768 | +/0.5443 | 0.8796 | | Non-Substrate | Non-Inhibitor |
|  | ZINC08918345 | +/0.8163 | +/0.8042 | +/0.5059 | 0.7149 | | Substrate | Non-Inhibitor |
|  | ZINC08991637 | +/0.9908 | +/0.9943 | +/0.7245 | 1.4649 | | Substrate | Inhibitor |
|  | ZINC08993096 | +/0.9929 | +/1.0000 | +/0.5779 | 1.4884 | | Non-Substrate | Inhibitor |
|  | ZINC09034003 | +/0.9220 | +/0.9921 | -/0.5883 | 0.6621 | | Substrate | Inhibitor |
|  | ZINC09034064 | +/0.6230 | +/0.8325 | -/0.6042 | 0.6453 | | Substrate | Inhibitor |
|  | ZINC09054134 | +/0.9917 | +/0.9822 | +/0.5806 | 1.1399 | | Non-Substrate | Non-Inhibitor |
|  | ZINC09089656 | +/0.9854 | +/0.9907 | -/0.6028 | 1.1658 | | Substrate | Non-Inhibitor |
|  | **ZINC09303152** | +/0.9819 | +/1.0000 | +/0.5916 | 1.4109 | | Non-Substrate | Non-Inhibitor |
|  | ZINC09341662 | +/0.9807 | +/0.9957 | +/0.6725 | 1.6344 | | Substrate | Inhibitor |
|  | ZINC09420846 | +/0.9856 | +/1.0000 | +/0.5588 | 1.4963 | | Non-Substrate | Inhibitor |
|  | ZINC09423653 | +/0.9895 | +/1.0000 | +/0.5705 | 1.4917 | | Non-Substrate | Inhibitor |
|  | ZINC09423883 | +/0.9943 | +/1.0000 | +/0.5928 | 1.5676 | | Non-Substrate | Inhibitor |
|  | ZINC09423968 | +/0.9895 | +/1.0000 | +/0.5705 | 1.4917 | | Non-Substrate | Inhibitor |
|  | ZINC09576136 | +/0.8860 | +/0.9912 | -/0.5577 | 0.7011 | | Non-Substrate | Inhibitor |
|  | ZINC11865187 | -/0.8726 | +/0.8101 | -/0.5969 | 0.3471 | | Non-Substrate | Non-Inhibitor |
|  | ZINC11865349 | +/0.6092 | +/0.7670 | -/0.6067 | 0.5643 | | Substrate | Non-Inhibitor |
|  | ZINC11865376 | +/0.9703 | +/0.9949 | -/0.5236 | 1.4688 | | Non-Substrate | Non-Inhibitor |
|  | ZINC11866483 | +/0.8642 | +/0.9672 | +/0.6813 | 0.8754 | | Substrate | Inhibitor |
|  | ZINC11866484 | +/0.8642 | +/0.9672 | +/0.6813 | 0.8754 | | Substrate | Inhibitor |
|  | ZINC11866485 | +/0.8845 | +/0.9795 | +/0.6892 | 0.9443 | | Substrate | Inhibitor |
|  | ZINC11866495 | +/0.8845 | +/0.9795 | +/0.6892 | 0.9443 | | Substrate | Inhibitor |
|  | ZINC11867066 | +/0.9121 | +/0.8937 | -/0.6495 | 0.3398 | | Substrate | Inhibitor |
|  | ZINC11867457 | +/0.8144 | +/0.9950 | +/0.6275 | 1.1700 | | Non-Substrate | Inhibitor |
|  | ZINC11867458 | +/0.8144 | +/0.9950 | +/0.6275 | 1.1700 | | Non-Substrate | Inhibitor |
|  | ZINC11867461 | +/0.9050 | +/0.9759 | +/0.6052 | 0.8809 | | Substrate | Inhibitor |
|  | ZINC11867462 | +/0.9050 | +/0.9759 | +/0.6052 | 0.8809 | | Substrate | Inhibitor |
|  | ZINC11867465 | +/0.8860 | +/0.9941 | -/0.5129 | 1.0170 | | Non-Substrate | Inhibitor |
|  | ZINC11867466 | +/0.8860 | +/0.9941 | -/0.5129 | 1.0170 | | Non-Substrate | Inhibitor |
|  | ZINC12296404 | +/0.7406 | +/0.9357 | +/0.6643 | 0.9918 | | Substrate | Inhibitor |
|  | ZINC12296477 | +/0.8865 | +/0.9950 | +/0.5000 | 0.9367 | | Non-Substrate | Inhibitor |
|  | ZINC12296478 | +/0.8865 | +/0.9950 | +/0.5000 | 0.9367 | | Non-Substrate | Inhibitor |
|  | ZINC12296500 | +/0.8750 | +/0.9760 | +/0.6499 | 0.8706 | | Substrate | Inhibitor |
|  | ZINC12296501 | +/0.8750 | +/0.9760 | +/0.6499 | 0.8706 | | Substrate | Inhibitor |
|  | ZINC12296551 | +/0.8862 | +/0.9812 | +/0.6774 | 0.8180 | | Substrate | Inhibitor |
|  | ZINC12296564 | +/0.8579 | +/0.9946 | +/0.5000 | 0.9673 | | Non-Substrate | Non-Inhibitor |
|  | ZINC12296565 | +/0.8579 | +/0.9946 | +/0.5000 | 0.9673 | | Non-Substrate | Non-Inhibitor |
|  | **ZINC12296580** | +/0.8028 | +/0.9595 | +/0.6004 | 1.1314 | | Non-Substrate | Non-Inhibitor |
|  | ZINC12296581 | +/0.8028 | +/0.9595 | +/0.6004 | 1.1314 | | Non-Substrate | Non-Inhibitor |
|  | ZINC12296700 | +/0.6691 | +/0.8995 | +/0.6354 | 0.6063 | | Substrate | Inhibitor |
|  | ZINC12296701 | +/0.6691 | +/0.8995 | +/0.6354 | 0.6063 | | Substrate | Inhibitor |
|  | ZINC12296761 | +/0.8440 | +/0. 9720 | +/0.7452 | 0.9311 | | Substrate | Inhibitor |
|  | ZINC12296762 | +/0.8440 | +/0. 9720 | +/0.7452 | 0.9311 | | Substrate | Inhibitor |
|  | ZINC12296864 | +/0.9068 | +/0.9779 | +/0.6007 | 0.7592 | | Substrate | Inhibitor |
|  | ZINC12296876 | +/0.8334 | +/0.9674 | +/0.7119 | 0.8557 | | Substrate | Inhibitor |
|  | ZINC12297013 | +/0.8334 | +/0.9674 | +/0.7119 | 0.8557 | | Substrate | Inhibitor |
|  | ZINC12297014 | +/0.8334 | +/0.9674 | +/0.7119 | 0.8557 | | Substrate | Inhibitor |
|  | ZINC12297019 | +/0.9449 | +/0.9785 | -/0.7457 | 0.5850 | | Substrate | Non-Inhibitor |
|  | **ZINC12297025** | +/0.8900 | +/0.9913 | +/0.7822 | 0.9481 | | Non-Substrate | Non-Inhibitor |
|  | ZINC12297042 | +/0.5058 | +/0.9836 | -/0.6370 | 0.5813 | | Substrate | Non-Inhibitor |
|  | ZINC12297091 | +/0.6842 | +/0.9448 | +/0.7280 | 1.0333 | | Substrate | Inhibitor |
|  | ZINC12297092 | +/0.6842 | +/0.9448 | +/0.7280 | 1.0333 | | Substrate | Inhibitor |
|  | ZINC12442585 | +/0.8916 | +/0.9953 | -/0.5493 | 1.0744 | | Substrate | Non-Inhibitor |
|  | ZINC12442587 | +/0.8916 | +/0.9953 | -/0.5493 | 1.0744 | | Substrate | Non-Inhibitor |
|  | ZINC12442588 | +/0.9450 | +/0.9880 | -/0.5484 | 0.8623 | | Substrate | Non-Inhibitor |
|  | ZINC12442590 | +/0.9450 | +/0.9880 | -/0.5484 | 0.8623 | | Substrate | Non-Inhibitor |
|  | ZINC12442592 | +/0.9301 | +/0.9937 | -/0.5672 | 0.7424 | | Substrate | Non-Inhibitor |
|  | ZINC12442594 | +/0.9301 | +/0.9937 | -/0.5672 | 0.7424 | | Substrate | Non-Inhibitor |
|  | ZINC12442597 | +/0.9422 | +/0.9953 | -/0.5559 | 0.9104 | | Substrate | Non-Inhibitor |
|  | ZINC12442599 | +/0.9422 | +/0.9953 | -/0.5559 | 0.9104 | | Substrate | Non-Inhibitor |
|  | ZINC12442608 | +/0.9378 | +/0.9839 | -/0.5452 | 0.9215 | | Substrate | Non-Inhibitor |
|  | ZINC12442610 | +/0.9378 | +/0.9839 | -/0.5452 | 0.9215 | | Substrate | Non-Inhibitor |
|  | ZINC12442649 | +/0.9909 | +/0.9936 | +/0.5519 | 1.0678 | | Substrate | Inhibitor |
|  | ZINC12442671 | +/0.9851 | +/1.0000 | +/0.5930 | 1.4597 | | Substrate | Non-Inhibitor |
|  | ZINC12602816 | +/0.8459 | +/0.9624 | -/0.5972 | 0.3424 | | Substrate | Inhibitor |
|  | ZINC12604806 | -/0.5114 | +/0.9878 | -/0.5463 | 0.9306 | | Substrate | Inhibitor |
|  | ZINC12660190 | +/0.5352 | +/0.7425 | -/0.6849 | 0.2320 | | Substrate | Inhibitor |
|  | ZINC12863193 | -/0.6946 | +/0.5599 | -/0.7427 | 0.1787 | | Substrate | Non-Inhibitor |
|  | ZINC12863203 | -/0.6946 | +/0.5599 | -/0.7427 | 0.1787 | | Substrate | Non-Inhibitor |
|  | ZINC12866401 | -/0.7068 | -/0.5123 | -/0.7342 | 0.1866 | | Substrate | Non-Inhibitor |
|  | ZINC12870752 | +/0.5725 | +/0.8548 | -/0.6067 | 0.5761 | | Substrate | Non-Inhibitor |
|  | ZINC12887203 | -/0.8946 | +/0.7519 | -/0.6428 | 0.5463 | | Substrate | Non-Inhibitor |
|  | ZINC12892129 | +/0.7213 | +/0.9217 | -/0.5383 | 0.9778 | | Substrate | Non-Inhibitor |
|  | ZINC12893265 | +/0.8871 | +/0.9593 | -/0.5909 | 0.7158 | | Non-Substrate | Inhibitor |
|  | ZINC12893333 | +/0.9687 | +/0.9835 | +/0.5907 | 0.9824 | | Substrate | Non-Inhibitor |
|  | ZINC12899484 | +/0.9068 | +/0.9779 | +/0.6007 | 0.7592 | | Substrate | Inhibitor |
|  | ZINC12899490 | +/0.9068 | +/0.9779 | +/0.6007 | 0.7592 | | Substrate | Inhibitor |
|  | ZINC13359662 | +/0.9754 | +/0.9621 | -/0.5266 | 0.8643 | | Substrate | Non-Inhibitor |
|  | ZINC13551971 | -/0.7656 | -/0.5310 | -/0.6795 | 0.0541 | | Substrate | Non-Inhibitor |
|  | ZINC13553109 | -/0.7492 | -/0.6130 | -/0.6796 | -0.0242 | | Substrate | Non-Inhibitor |
|  | ZINC13683907 | +/0.9909 | +/0.9936 | +/0.5519 | 1.0678 | | Substrate | Inhibitor |
|  | ZINC13687938 | +/0.9897 | +/0.9970 | +/0.5722 | 1.3452 | | Non-Substrate | Non-Inhibitor |
|  | ZINC13692664 | +/0.9784 | +/0.9895 | +/0.6787 | 1.3715 | | Substrate | Inhibitor |
|  | ZINC13720979 | +/0.9917 | +/0.9959 | +/0.6460 | 1.3169 | | Non-Substrate | Inhibitor |
|  | ZINC13721203 | +/0.9893 | +/0.9955 | +/0.5600 | 1.1910 | | Non-Substrate | Inhibitor |
|  | ZINC13731223 | +/0.9851 | +/1.0000 | +/0.5930 | 1.4597 | | Substrate | Non-Inhibitor |
|  | ZINC13732746 | +/0.9917 | +/0.9959 | +/0.6460 | 1.3169 | | Non-Substrate | Inhibitor |
|  | ZINC13732895 | +/0.9883 | +/0.9866 | +/0.6434 | 1.3644 | | Substrate | Inhibitor |
|  | ZINC13736331 | +/0.9925 | +/0.9958 | +/0.7136 | 1.3871 | | Substrate | Inhibitor |
|  | ZINC15303561 | +/0.8818 | +/0.9767 | -/0.5925 | 0.5403 | | Substrate | Non-Inhibitor |
|  | ZINC15303564 | +/0.8818 | +/0.9767 | -/0.5925 | 0.5403 | | Substrate | Non-Inhibitor |
|  | ZINC15957613 | +/0.5328 | +/0.8149 | -/0.6406 | 0.3670 | | Substrate | Non-Inhibitor |
|  | ZINC15959322 | +/0.8345 | +/0.9800 | -/0.6311 | 0.5522 | | Substrate | Non-Inhibitor |
|  | ZINC15959323 | +/0.8345 | +/0.9800 | -/0.6311 | 0.5522 | | Substrate | Non-Inhibitor |
|  | ZINC15959325 | +/0.8345 | +/0.9800 | -/0.6311 | 0.5522 | | Substrate | Non-Inhibitor |
|  | ZINC15967689 | +/0.7807 | +/0.9864 | -/0.5836 | 0.5678 | | Substrate | Non-Inhibitor |
|  | ZINC15967691 | +/0.7807 | +/0.9864 | -/0.5836 | 0.5678 | | Substrate | Non-Inhibitor |
|  | ZINC15967694 | +/0.7807 | +/0.9864 | -/0.5836 | 0.5678 | | Substrate | Non-Inhibitor |
|  | ZINC15969172 | +/0.8345 | +/0.9800 | -/0.6311 | 0.5522 | | Substrate | Non-Inhibitor |
|  | ZINC15969174 | +/0.8345 | +/0.9800 | -/0.6311 | 0.5522 | | Substrate | Non-Inhibitor |
|  | ZINC15969569 | +/0.7452 | +/0.9567 | -/0.6322 | 0.5219 | | Substrate | Non-Inhibitor |
|  | ZINC15969571 | +/0.7452 | +/0.9567 | -/0.6322 | 0.5219 | | Substrate | Non-Inhibitor |
|  | ZINC15969573 | +/0.7452 | +/0.9567 | -/0.6322 | 0.5219 | | Substrate | Non-Inhibitor |
|  | ZINC16026354 | -/0.6099 | +/0.9658 | -/0.6305 | 0.3870 | | Substrate | Non-Inhibitor |
|  | **ZINC16292828** | +/0.9819 | +/1.0000 | +/0.5916 | 1.4109 | | Non-Substrate | Non-Inhibitor |
|  | ZINC16972169 | +/0.8932 | +/0.9650 | -/0.6525 | 0.6480 | | Substrate | Non-Inhibitor |
|  | ZINC16995797 | +/0.8485 | +/0.9702 | -/0.6760 | 0.5659 | | Substrate | Non-Inhibitor |
|  | ZINC17014720 | +/0.8198 | +/0.9703 | -/0.6577 | 0.6782 | | Substrate | Non-Inhibitor |
|  | ZINC17015252 | +/0.6221 | +/0.9830 | -/0.5978 | 1.0124 | | Substrate | Non-Inhibitor |
|  | ZINC17029354 | +/0.7629 | +/0.9730 | -/0.6377 | 0.7232 | | Substrate | Non-Inhibitor |
|  | ZINC17194529 | +/0.8044 | +/0.9795 | -/0.6430 | 0.7478 | | Substrate | Non-Inhibitor |
|  | ZINC17195517 | +/0.8198 | +/0.9703 | -/0.6577 | | 0.6782 | Substrate | Non-Inhibitor |
|  | ZINC17196472 | +/0.7970 | +/0.9580 | -/0.6607 | | 0.6282 | Substrate | Non-Inhibitor |
|  | ZINC17196754 | +/0.6200 | +/0.9802 | -/0.6446 | | 0.7678 | Substrate | Non-Inhibitor |
|  | ZINC18091170 | +/0.9900 | +/0.9970 | +/0.5516 | | 1.3295 | Non-Substrate | Inhibitor |
|  | ZINC18176990 | +/0.9827 | +/1.0000 | +/0.5493 | | 1.3154 | Non-Substrate | Inhibitor |
|  | ZINC18207570 | +/0.8044 | +/0.9795 | -/0.6430 | | 0.7478 | Substrate | Non-Inhibitor |
|  | ZINC18219349 | +/0.9743 | +/0.9955 | +/0.6186 | | 1.2937 | Non-Substrate | Inhibitor |
|  | ZINC19891473 | -/0.6099 | +/0.9658 | -/0.6305 | | 0.3870 | Substrate | Non-Inhibitor |
|  | ZINC20411267 | +/0.7629 | +/0.9730 | -/0.6377 | | 0.7232 | Substrate | Non-Inhibitor |
|  | ZINC20411826 | +/0.8485 | +/0.9702 | -/0.6760 | | 0.5659 | Substrate | Non-Inhibitor |
|  | ZINC20412340 | +/0.7629 | +/0.9730 | -/0.6377 | | 0.7232 | Substrate | Non-Inhibitor |
|  | ZINC20611122 | +/0.8345 | +/0.9800 | -/0.6311 | | 0.5522 | Substrate | Non-Inhibitor |
|  | ZINC31169877 | -/0.7400 | +/0.9830 | -/0.6231 | | 0.7576 | Substrate | Non-Inhibitor |
|  | ZINC32502304 | +/0.7867 | +/0.7762 | -/0.5440 | | 0.7611 | Substrate | Non-Inhibitor |
|  | ZINC32502306 | +/0.7926 | +/0.9834 | -/0.5648 | | 0.9750 | Substrate | Non-Inhibitor |
|  | ZINC34300440 | +/0.8862 | +/0.9924 | +/0.6403 | | 1.5319 | Substrate | Non-Inhibitor |
|  | ZINC35365995 | -/0.7785 | +/0.9534 | -/0.6846 | | 0.2892 | Substrate | Non-Inhibitor |
|  | ZINC35365997 | -/0.7785 | +/0.9534 | -/0.6846 | | 0.2892 | Substrate | Non-Inhibitor |
|  | ZINC35457807 | +/0.9348 | +/0.9953 | +/0.5314 | | 1.0602 | Substrate | Non-Inhibitor |
|  | ZINC36369885 | +/0.8553 | +/0.9943 | +/0.6010 | | 1.2392 | Substrate | Non-Inhibitor |
|  | ZINC38167070 | +/0.8332 | +/0.9883 | -/0.5405 | | 0.8479 | Non-Substrate | Non-Inhibitor |
|  | ZINC38167083 | +/0.9303 | +/0.9974 | -/0.5520 | | 1.0691 | Non-Substrate | Non-Inhibitor |
|  | ZINC54278174 | +/0.9892 | +/0.9845 | +/0.6307 | | 1.3018 | Non-Substrate | Non-Inhibitor |
|  | ZINC54278181 | +/0.9887 | +/0.9793 | +/0.6393 | | 1.3323 | Non-Substrate | Non-Inhibitor |
|  | ZINC54278191 | +/0.9836 | +/0.9870 | +/0.6049 | | 1.2110 | Non-Substrate | Non-Inhibitor |
|  | ZINC54333105 | +/0.9284 | +/0.9947 | -/0.5665 | | 0.7624 | Substrate | Non-Inhibitor |
|  | ZINC54333106 | +/0.9284 | +/0.9947 | -/0.5665 | | 0.7624 | Substrate | Non-Inhibitor |
|  | ZINC55396209 | +/0.9928 | +/0.9628 | +/0.5636 | | 1.0096 | Substrate | Non-Inhibitor |
|  | ZINC58090636 | +/0.9913 | +/0.9739 | +/0.5620 | | 1.0611 | Non-Substrate | Non-Inhibitor |
|  | ZINC61997665 | +/0.9691 | +/0.9968 | +/0.6893 | | 1.4510 | Substrate | Non-Inhibitor |
|  | ZINC61997666 | +/0.9691 | +/0.9968 | +/0.6893 | | 1.4510 | Substrate | Non-Inhibitor |
|  | ZINC61997705 | +/0.9774 | +/0.9890 | +/0.6172 | | 1.2409 | Non-Substrate | Non-Inhibitor |
|  | ZINC62001268 | +/0.9852 | +/0.9975 | -/0.5695 | | 0.6642 | Substrate | Non-Inhibitor |
|  | ZINC67913130 | +/0.9189 | +/0.9874 | +/0.5379 | | 1.2048 | Substrate | Non-Inhibitor |
|  | ZINC68569286 | +/0.9040 | +/1.0000 | +/0.6979 | | 1.2963 | Non-Substrate | Non-Inhibitor |
|  | **ZINC68569602** | +/0.8731 | +/0.9195 | -/0.5880 | | 0.7497 | Non-Substrate | Non-Inhibitor |
|  | ZINC68572120 | +/0.9384 | +/0.8458 | +/0.5834 | | 1.4663 | Substrate | Inhibitor |
|  | **ZINC68583170** | +/0.9800 | +/0.9005 | +/0.5245 | | 1.0816 | Non-Substrate | Non-Inhibitor |
|  | ZINC68583182 | +/0.9730 | +/0.9922 | +/0.5523 | | 1.0115 | Non-Substrate | Inhibitor |
|  | ZINC68589458 | +/0.9869 | +/1.0000 | +/0.7017 | | 1.6066 | Non-Substrate | Inhibitor |
|  | ZINC68589462 | +/0.9869 | +/1.0000 | +/0.7017 | | 1.6066 | Non-Substrate | Inhibitor |
|  | ZINC68591854 | +/0.9382 | +/0.9140 | +/0.6380 | | 1.3617 | Non-Substrate | Inhibitor |
|  | ZINC68591857 | +/0.9382 | +/0.9140 | +/0.6380 | | 1.3617 | Non-Substrate | Inhibitor |

**Supplementary Table S3.** *in-silico* Cyp450 enzyme metabolism profile was obtained from admetSAR server for selected 330 compounds from virtual screening. Selected compounds (14) for re-docking were highlighted in bold.

| **Sr. No.** | **Zinc ID** | **CYP-2C9 substrate/inhibitor** | **CYP-2D6 substrate/inhibitor** | **CYP-3A4 substrate/inhibitor** | **CYP-1A2 inhibitor** | **CYP-2C19 inhibitor** | **CYP inhibitory promiscuity** | |
| --- | --- | --- | --- | --- | --- | --- | --- | --- |
|  | ZINC00120631 | Non-Substrate/Non-Inhibitor | Non-Substrate/Non-Inhibitor | Substrate/Non-Inhibitor | Non-Inhibitor | Inhibitor | Low | |
|  | ZINC00238468 | Non-Substrate/Inhibitor | Non-Substrate/Non-Inhibitor | Non-Substrate/Non-Inhibitor | Inhibitor | Inhibitor | High | |
|  | ZINC00338371 | Non-Substrate/Non-Inhibitor | Non-Substrate/Non-Inhibitor | Substrate/Inhibitor | Inhibitor | Non-Inhibitor | High | |
|  | ZINC00408721 | Non-Substrate/Non-Inhibitor | Non-Substrate/Inhibitor | Non-Substrate/Non-Inhibitor | Non-Inhibitor | Non-Inhibitor | Low | |
|  | ZINC00490486 | Non-Substrate/Inhibitor | Non-Substrate/Non-Inhibitor | Non-Substrate/Inhibitor | Inhibitor | Inhibitor | High | |
|  | ZINC00519086 | Non-Substrate/Inhibitor | Non-Substrate/Non-Inhibitor | Non-Substrate/Non-Inhibitor | Inhibitor | Inhibitor | High | |
|  | ZINC01482030 | Non-Substrate/Non-Inhibitor | Substrate/Inhibitor | Substrate/Non-Inhibitor | Non-Inhibitor | Non-Inhibitor | Low | |
|  | ZINC01686586 | Non-Substrate/Inhibitor | Non-Substrate/Inhibitor | Non-Substrate/Non-Inhibitor | Inhibitor | Inhibitor | High | |
|  | ZINC01726969 | Non-Substrate/Non-Inhibitor | Non-Substrate/Non-Inhibitor | Non-Substrate/Non-Inhibitor | Inhibitor | Non-Inhibitor | Low | |
|  | ZINC01787663 | Non-Substrate/Non-Inhibitor | Non-Substrate/Non-Inhibitor | Non-Substrate/Non-Inhibitor | Non-Inhibitor | Non-Inhibitor | Low | |
|  | ZINC01787672 | Non-Substrate/Non-Inhibitor | Non-Substrate/Non-Inhibitor | Non-Substrate/Non-Inhibitor | Non-Inhibitor | Non-Inhibitor | Low | |
|  | ZINC01845382 | Non-Substrate/Non-Inhibitor | Non-Substrate/Non-Inhibitor | Substrate/Inhibitor | Non-Inhibitor | Inhibitor | High | |
|  | ZINC01886047 | Non-Substrate/Inhibitor | Non-Substrate/Non-Inhibitor | Substrate/Inhibitor | Non-Inhibitor | Inhibitor | High | |
|  | ZINC02001186 | Non-Substrate/Non-Inhibitor | Non-Substrate/Non-Inhibitor | Non-Substrate/Non-Inhibitor | Inhibitor | Non-Inhibitor | Low | |
|  | ZINC02092089 | Non-Substrate/Non-Inhibitor | Non-Substrate/Non-Inhibitor | Substrate/Inhibitor | Inhibitor | Inhibitor | High | |
|  | ZINC02092130 | Non-Substrate/Inhibitor | Non-Substrate/Non-Inhibitor | Substrate/Inhibitor | Non-Inhibitor | Inhibitor | High | |
|  | ZINC02092414 | Non-Substrate/Inhibitor | Non-Substrate/Non-Inhibitor | Substrate/Non-Inhibitor | Non-Inhibitor | Inhibitor | High | |
|  | ZINC02092422 | Non-Substrate/Inhibitor | Non-Substrate/Non-Inhibitor | Substrate/Inhibitor | Non-Inhibitor | Inhibitor | High | |
|  | ZINC02092475 | Non-Substrate/Non-Inhibitor | Non-Substrate/Non-Inhibitor | Substrate/Inhibitor | Inhibitor | Inhibitor | High | |
|  | ZINC02092931 | Non-Substrate/Non-Inhibitor | Non-Substrate/Non-Inhibitor | Substrate/Inhibitor | Non-Inhibitor | Inhibitor | High | |
|  | ZINC02093637 | Non-Substrate/Non-Inhibitor | Non-Substrate/Non-Inhibitor | Substrate/Inhibitor | Non-Inhibitor | Inhibitor | High | |
|  | ZINC02094632 | Non-Substrate/Inhibitor | Non-Substrate/Non-Inhibitor | Substrate/Inhibitor | Non-Inhibitor | Inhibitor | High | |
|  | ZINC02095117 | Non-Substrate/Inhibitor | Non-Substrate/Non-Inhibitor | Substrate/Non-Inhibitor | Non-Inhibitor | Non-Inhibitor | High | |
|  | ZINC02095140 | Non-Substrate/Inhibitor | Non-Substrate/Non-Inhibitor | Substrate/Non-Inhibitor | Non-Inhibitor | Inhibitor | High | |
|  | ZINC02095426 | Non-Substrate/Non-Inhibitor | Non-Substrate/Non-Inhibitor | Substrate/Non-Inhibitor | Non-Inhibitor | Inhibitor | High | |
|  | ZINC02096038 | Non-Substrate/Non-Inhibitor | Non-Substrate/Non-Inhibitor | Substrate/Non-Inhibitor | Non-Inhibitor | Non-Inhibitor | High | |
|  | ZINC02096725 | Non-Substrate/Inhibitor | Non-Substrate/Non-Inhibitor | Substrate/Inhibitor | Non-Inhibitor | Inhibitor | High | |
|  | ZINC02098157 | Non-Substrate/Inhibitor | Non-Substrate/Non-Inhibitor | Substrate/Inhibitor | Non-Inhibitor | Inhibitor | High | |
|  | ZINC02098972 | Non-Substrate/Non-Inhibitor | Non-Substrate/Non-Inhibitor | Substrate/Inhibitor | Non-Inhibitor | Inhibitor | High | |
|  | ZINC02100762 | Non-Substrate/Non-Inhibitor | Non-Substrate/Non-Inhibitor | Substrate/Inhibitor | Non-Inhibitor | Non-Inhibitor | High | |
|  | ZINC02101594 | Non-Substrate/Inhibitor | Non-Substrate/Non-Inhibitor | Substrate/Inhibitor | Non-Inhibitor | Inhibitor | High | |
|  | ZINC02101693 | Non-Substrate/Inhibitor | Non-Substrate/Inhibitor | Substrate/Non-Inhibitor | Non-Inhibitor | Non-Inhibitor | High | |
|  | ZINC02101695 | Non-Substrate/Inhibitor | Non-Substrate/Inhibitor | Substrate/Non-Inhibitor | Non-Inhibitor | Non-Inhibitor | High | |
|  | ZINC02102083 | Non-Substrate/Non-Inhibitor | Non-Substrate/Non-Inhibitor | Substrate/Non-Inhibitor | Non-Inhibitor | Non-Inhibitor | Low | |
|  | ZINC02102560 | Non-Substrate/Inhibitor | Non-Substrate/Non-Inhibitor | Substrate/Inhibitor | Non-Inhibitor | Inhibitor | High | |
|  | ZINC02103115 | Non-Substrate/Non-Inhibitor | Non-Substrate/Non-Inhibitor | Substrate/Non-Inhibitor | Non-Inhibitor | Inhibitor | High | |
|  | ZINC02103122 | Non-Substrate/Non-Inhibitor | Non-Substrate/Non-Inhibitor | Substrate/Inhibitor | Non-Inhibitor | Non-Inhibitor | High | |
|  | ZINC02103383 | Non-Substrate/Inhibitor | Non-Substrate/Non-Inhibitor | Substrate/Inhibitor | Non-Inhibitor | Inhibitor | High | |
|  | ZINC02103425 | Non-Substrate/Non-Inhibitor | Non-Substrate/Non-Inhibitor | Substrate/Non-Inhibitor | Non-Inhibitor | Non-Inhibitor | High | |
|  | ZINC02103558 | Non-Substrate/Inhibitor | Non-Substrate/Non-Inhibitor | Substrate/Inhibitor | Non-Inhibitor | Inhibitor | High | |
|  | ZINC02103644 | Non-Substrate/Inhibitor | Non-Substrate/Non-Inhibitor | Substrate/Inhibitor | Non-Inhibitor | Inhibitor | High | |
|  | ZINC02104482 | Non-Substrate/Inhibitor | Non-Substrate/Non-Inhibitor | Substrate/Inhibitor | Non-Inhibitor | Inhibitor | High | |
|  | ZINC02105209 | Non-Substrate/Non-Inhibitor | Non-Substrate/Non-Inhibitor | Substrate/Non-Inhibitor | Non-Inhibitor | Non-Inhibitor | High | |
|  | ZINC02106269 | Non-Substrate/Non-Inhibitor | Non-Substrate/Non-Inhibitor | Substrate/Inhibitor | Non-Inhibitor | Inhibitor | High | |
|  | ZINC02106282 | Non-Substrate/Non-Inhibitor | Non-Substrate/Non-Inhibitor | Substrate/Non-Inhibitor | Non-Inhibitor | Non-Inhibitor | High | |
|  | ZINC02107064 | Non-Substrate/Inhibitor | Non-Substrate/Non-Inhibitor | Substrate/Inhibitor | Non-Inhibitor | Inhibitor | High | |
|  | ZINC02107810 | Non-Substrate/Inhibitor | Non-Substrate/Non-Inhibitor | Substrate/Inhibitor | Non-Inhibitor | Inhibitor | High | |
|  | ZINC02108288 | Non-Substrate/Non-Inhibitor | Non-Substrate/Non-Inhibitor | Substrate/Inhibitor | Inhibitor | Inhibitor | High | |
|  | ZINC02108366 | Non-Substrate/Non-Inhibitor | Non-Substrate/Non-Inhibitor | Substrate/Non-Inhibitor | Non-Inhibitor | Inhibitor | High | |
|  | ZINC02108756 | Non-Substrate/Inhibitor | Non-Substrate/Non-Inhibitor | Non-Substrate/Non-Inhibitor | Inhibitor | Non-Inhibitor | High | |
|  | ZINC02109073 | Non-Substrate/Inhibitor | Non-Substrate/Non-Inhibitor | Substrate/Inhibitor | Non-Inhibitor | Inhibitor | High | |
|  | ZINC02109448 |  |  |  |  |  |  | |
|  | ZINC02109745 | Non-Substrate/Inhibitor | Non-Substrate/Non-Inhibitor | Substrate/Inhibitor | Non-Inhibitor | Inhibitor | High | |
|  | ZINC02111048 | Non-Substrate/Inhibitor | Non-Substrate/Non-Inhibitor | Non-Substrate/Non-Inhibitor | Inhibitor | Non-Inhibitor | High | |
|  | ZINC02111151 | Non-Substrate/Inhibitor | Non-Substrate/Non-Inhibitor | Substrate/Inhibitor | Non-Inhibitor | Inhibitor | High | |
|  | ZINC02111981 | Non-Substrate/Non-Inhibitor | Non-Substrate/Non-Inhibitor | Substrate/Non-Inhibitor | Inhibitor | Inhibitor | High | |
|  | ZINC02112230 | Non-Substrate/Non-Inhibitor | Non-Substrate/Non-Inhibitor | Substrate/Inhibitor | Non-Inhibitor | Inhibitor | High | |
|  | ZINC02112613 | Non-Substrate/Non-Inhibitor | Non-Substrate/Non-Inhibitor | Substrate/Inhibitor | Non-Inhibitor | Inhibitor | High | |
|  | ZINC02113936 | Non-Substrate/Non-Inhibitor | Non-Substrate/Non-Inhibitor | Substrate/Non-Inhibitor | Non-Inhibitor | Non-Inhibitor | Low | |
|  | ZINC02115702 | Non-Substrate/Non-Inhibitor | Non-Substrate/Non-Inhibitor | Substrate/Non-Inhibitor | Non-Inhibitor | Non-Inhibitor | Low | |
|  | ZINC02117133 | Non-Substrate/Inhibitor | Non-Substrate/Non-Inhibitor | Substrate/Non-Inhibitor | Non-Inhibitor | Inhibitor | High | |
|  | ZINC02117888 | Non-Substrate/Non-Inhibitor | Non-Substrate/Non-Inhibitor | Substrate/Non-Inhibitor | Non-Inhibitor | Non-Inhibitor | Low | |
|  | ZINC02118353 | Non-Substrate/Non-Inhibitor | Non-Substrate/Non-Inhibitor | Substrate/Non-Inhibitor | Non-Inhibitor | Non-Inhibitor | High | |
|  | ZINC02118360 | Non-Substrate/Non-Inhibitor | Non-Substrate/Non-Inhibitor | Substrate/Inhibitor | Inhibitor | Inhibitor | High | |
|  | ZINC02118901 | Non-Substrate/Non-Inhibitor | Non-Substrate/Non-Inhibitor | Substrate/Inhibitor | Inhibitor | Inhibitor | High | |
|  | ZINC02120774 | Non-Substrate/Inhibitor | Non-Substrate/Non-Inhibitor | Substrate/Inhibitor | Inhibitor | Inhibitor | High | |
|  | ZINC02122409 | Non-Substrate/Non-Inhibitor | Non-Substrate/Non-Inhibitor | Substrate/Inhibitor | Inhibitor | Inhibitor | High | |
|  | **ZINC02123081** | Non-Substrate/Inhibitor | Non-Substrate/Non-Inhibitor | Non-Substrate/Non-Inhibitor | Non-Inhibitor | Non-Inhibitor | Low | |
|  | ZINC02123191 | Non-Substrate/Non-Inhibitor | Non-Substrate/Inhibitor | Substrate/Inhibitor | Inhibitor | Inhibitor | High | |
|  | ZINC02123282 | Non-Substrate/Non-Inhibitor | Non-Substrate/Non-Inhibitor | Substrate/Non-Inhibitor | Non-Inhibitor | Non-Inhibitor | Low | |
|  | ZINC02123424 | Non-Substrate/Non-Inhibitor | Non-Substrate/Non-Inhibitor | Substrate/Inhibitor | Inhibitor | Inhibitor | High | |
|  | ZINC02123859 | Non-Substrate/Non-Inhibitor | Non-Substrate/Non-Inhibitor | Substrate/Inhibitor | Inhibitor | Inhibitor | High | |
|  | ZINC02125038 | Non-Substrate/Non-Inhibitor | Non-Substrate/Non-Inhibitor | Substrate/Inhibitor | Inhibitor | Inhibitor | High | |
|  | ZINC02125714 | Non-Substrate/Non-Inhibitor | Non-Substrate/Non-Inhibitor | Substrate/Inhibitor | Non-Inhibitor | Inhibitor | High | |
|  | ZINC02148197 | Non-Substrate/Inhibitor | Non-Substrate/Non-Inhibitor | Substrate/Inhibitor | Non-Inhibitor | Inhibitor | High | |
|  | ZINC02148481 | Non-Substrate/Non-Inhibitor | Non-Substrate/Non-Inhibitor | Substrate/Non-Inhibitor | Non-Inhibitor | Inhibitor | Low | |
|  | ZINC02148655 | Non-Substrate/Inhibitor | Non-Substrate/Non-Inhibitor | Substrate/Inhibitor | Non-Inhibitor | Inhibitor | High | |
|  | ZINC02148909 | Non-Substrate/Non-Inhibitor | Non-Substrate/Non-Inhibitor | Substrate/Non-Inhibitor | Non-Inhibitor | Non-Inhibitor | High | |
|  | ZINC02148919 | Non-Substrate/Non-Inhibitor | Non-Substrate/Non-Inhibitor | Substrate/Non-Inhibitor | Non-Inhibitor | Non-Inhibitor | Low | |
|  | ZINC02148935 | Non-Substrate/Inhibitor | Non-Substrate/Non-Inhibitor | Substrate/Non-Inhibitor | Non-Inhibitor | Inhibitor | High | |
|  | ZINC02149492 | Non-Substrate/Inhibitor | Non-Substrate/Non-Inhibitor | Substrate/Inhibitor | Non-Inhibitor | Non-Inhibitor | High | |
|  | ZINC02150498 | Non-Substrate/Inhibitor | Non-Substrate/Non-Inhibitor | Substrate/Non-Inhibitor | Non-Inhibitor | Inhibitor | High | |
|  | ZINC02151835 | Non-Substrate/Non-Inhibitor | Non-Substrate/Non-Inhibitor | Substrate/Non-Inhibitor | Non-Inhibitor | Non-Inhibitor | Low | |
|  | ZINC02151836 | Non-Substrate/Non-Inhibitor | Non-Substrate/Non-Inhibitor | Substrate/Non-Inhibitor | Non-Inhibitor | Non-Inhibitor | Low | |
|  | ZINC02154469 | Non-Substrate/Inhibitor | Non-Substrate/Non-Inhibitor | Substrate/Inhibitor | Non-Inhibitor | Inhibitor | High | |
|  | ZINC02154866 | Non-Substrate/Non-Inhibitor | Non-Substrate/Non-Inhibitor | Substrate/Non-Inhibitor | Non-Inhibitor | Inhibitor | High | |
|  | ZINC02155992 | Non-Substrate/Inhibitor | Non-Substrate/Non-Inhibitor | Substrate/Inhibitor | Non-Inhibitor | Inhibitor | High | |
|  | ZINC02156445 | Non-Substrate/Inhibitor | Non-Substrate/Non-Inhibitor | Substrate/Inhibitor | Non-Inhibitor | Inhibitor | High | |
|  | ZINC02156984 | Non-Substrate/Non-Inhibitor | Non-Substrate/Non-Inhibitor | Substrate/Non-Inhibitor | Inhibitor | Inhibitor | High | |
|  | ZINC02156988 | Non-Substrate/Non-Inhibitor | Non-Substrate/Non-Inhibitor | Substrate/Non-Inhibitor | Inhibitor | Inhibitor | High | |
|  | ZINC02157224 | Non-Substrate/Inhibitor | Non-Substrate/Non-Inhibitor | Non-Substrate/Non-Inhibitor | Inhibitor | Non-Inhibitor | High | |
|  | ZINC02158043 | Non-Substrate/Inhibitor | Non-Substrate/Non-Inhibitor | Substrate/Inhibitor | Non-Inhibitor | Inhibitor | High | |
|  | ZINC02158790 | Non-Substrate/Non-Inhibitor | Non-Substrate/Non-Inhibitor | Non-Substrate/Non-Inhibitor | Non-Inhibitor | Non-Inhibitor | Low | |
|  | ZINC02159274 | Non-Substrate/Non-Inhibitor | Non-Substrate/Non-Inhibitor | Substrate/Inhibitor | Non-Inhibitor | Non-Inhibitor | High | |
|  | ZINC02159526 | Non-Substrate/Non-Inhibitor | Non-Substrate/Non-Inhibitor | Substrate/Inhibitor | Inhibitor | Inhibitor | High | |
|  | ZINC02159783 | Non-Substrate/Non-Inhibitor | Non-Substrate/Non-Inhibitor | Substrate/Inhibitor | Non-Inhibitor | Inhibitor | High | |
|  | ZINC02160471 | Non-Substrate/Non-Inhibitor | Non-Substrate/Non-Inhibitor | Substrate/Inhibitor | Inhibitor | Inhibitor | High | |
|  | ZINC02503427 | Non-Substrate/Inhibitor | Non-Substrate/Non-Inhibitor | Substrate/Inhibitor | Inhibitor | Inhibitor | High | |
|  | ZINC03842059 | Non-Substrate/Non-Inhibitor | Non-Substrate/Non-Inhibitor | Substrate/Inhibitor | Non-Inhibitor | Non-Inhibitor | High | |
|  | ZINC03843035 | Non-Substrate/Non-Inhibitor | Non-Substrate/Non-Inhibitor | Substrate/Non-Inhibitor | Inhibitor | Inhibitor | High | |
|  | **ZINC03843365** | Non-Substrate/Non-Inhibitor | Non-Substrate/Non-Inhibitor | Non-Substrate/Non-Inhibitor | Non-Inhibitor | Inhibitor | Low | |
|  | ZINC03844760 | Non-Substrate/Inhibitor | Non-Substrate/Non-Inhibitor | Substrate/Inhibitor | Inhibitor | Inhibitor | High | |
|  | ZINC03845096 | Non-Substrate/Inhibitor | Non-Substrate/Non-Inhibitor | Substrate/Inhibitor | Inhibitor | Non-Inhibitor | High | |
|  | **ZINC03845323** | Non-Substrate/Non-Inhibitor | Non-Substrate/Non-Inhibitor | Non-Substrate/Non-Inhibitor | Non-Inhibitor | Non-Inhibitor | Low | |
|  | ZINC03845515 | Non-Substrate/Inhibitor | Non-Substrate/Non-Inhibitor | Substrate/Non-Inhibitor | Inhibitor | Inhibitor | High | |
|  | ZINC03845516 | Non-Substrate/Inhibitor | Non-Substrate/Non-Inhibitor | Substrate/Non-Inhibitor | Inhibitor | Inhibitor | High | |
|  | ZINC03846581 | Non-Substrate/Non-Inhibitor | Non-Substrate/Non-Inhibitor | Substrate/Non-Inhibitor | Non-Inhibitor | Non-Inhibitor | High | |
|  | ZINC03846582 | Non-Substrate/Non-Inhibitor | Non-Substrate/Non-Inhibitor | Substrate/Non-Inhibitor | Non-Inhibitor | Non-Inhibitor | High | |
|  | ZINC03846898 | Non-Substrate/Inhibitor | Non-Substrate/Non-Inhibitor | Non-Substrate/Non-Inhibitor | Non-Inhibitor | Non-Inhibitor | High | |
|  | ZINC03847083 | Non-Substrate/Non-Inhibitor | Non-Substrate/Non-Inhibitor | Non-Substrate/Non-Inhibitor | Inhibitor | Non-Inhibitor | Low | |
|  | ZINC03847184 | Non-Substrate/Non-Inhibitor | Non-Substrate/Non-Inhibitor | Substrate/Non-Inhibitor | Non-Inhibitor | Non-Inhibitor | Low | |
|  | ZINC03848443 | Non-Substrate/Non-Inhibitor | Non-Substrate/Non-Inhibitor | Substrate/Inhibitor | Inhibitor | Inhibitor | High | |
|  | ZINC03848917 | Non-Substrate/Inhibitor | Non-Substrate/Non-Inhibitor | Substrate/Inhibitor | Inhibitor | Inhibitor | High | |
|  | **ZINC03849421** | Non-Substrate/Non-Inhibitor | Non-Substrate/Non-Inhibitor | Non-Substrate/Non-Inhibitor | Inhibitor | Non-Inhibitor | Low | |
|  | ZINC03851871 | Non-Substrate/Non-Inhibitor | Non-Substrate/Non-Inhibitor | Non-Substrate/Non-Inhibitor | Inhibitor | Non-Inhibitor | Low | |
|  | ZINC03872986 | Non-Substrate/Non-Inhibitor | Non-Substrate/Non-Inhibitor | Substrate/Non-Inhibitor | Non-Inhibitor | Non-Inhibitor | Low | |
|  | ZINC03927200 | Non-Substrate/Non-Inhibitor | Non-Substrate/Non-Inhibitor | Substrate/Non-Inhibitor | Non-Inhibitor | Non-Inhibitor | Low | |
|  | ZINC03960761 | Non-Substrate/Non-Inhibitor | Non-Substrate/Inhibitor | Non-Substrate/Non-Inhibitor | Inhibitor | Non-Inhibitor | High | |
|  | ZINC03979002 | Non-Substrate/Inhibitor | Non-Substrate/Non-Inhibitor | Non-Substrate/Non-Inhibitor | Non-Inhibitor | Non-Inhibitor | Low | |
|  | ZINC04017533 | Non-Substrate/Inhibitor | Non-Substrate/Non-Inhibitor | Substrate/Non-Inhibitor | Inhibitor | Non-Inhibitor | Low | |
|  | ZINC04026127 | Non-Substrate/Non-Inhibitor | Non-Substrate/Non-Inhibitor | Substrate/Non-Inhibitor | Non-Inhibitor | Non-Inhibitor | Low | |
|  | ZINC04026666 | Non-Substrate/Non-Inhibitor | Non-Substrate/Non-Inhibitor | Substrate/Inhibitor | Inhibitor | Non-Inhibitor | High | |
|  | ZINC04027261 | Non-Substrate/Non-Inhibitor | Non-Substrate/Non-Inhibitor | Substrate/Non-Inhibitor | Non-Inhibitor | Non-Inhibitor | Low | |
|  | ZINC04046706 | Non-Substrate/Non-Inhibitor | Non-Substrate/Non-Inhibitor | Substrate/Non-Inhibitor | Non-Inhibitor | Non-Inhibitor | Low | |
|  | ZINC04062050 | Non-Substrate/Non-Inhibitor | Non-Substrate/Non-Inhibitor | Substrate/Non-Inhibitor | Non-Inhibitor | Inhibitor | High | |
|  | ZINC04073740 | Non-Substrate/Non-Inhibitor | Non-Substrate/Non-Inhibitor | Substrate/Non-Inhibitor | Non-Inhibitor | Non-Inhibitor | Low | |
|  | ZINC04084783 | Non-Substrate/Non-Inhibitor | Non-Substrate/Non-Inhibitor | Non-Substrate/Non-Inhibitor | Non-Inhibitor | Non-Inhibitor | Low | |
|  | ZINC04084832 | Non-Substrate/Non-Inhibitor | Non-Substrate/Non-Inhibitor | Substrate/Inhibitor | Inhibitor | Non-Inhibitor | High | |
|  | ZINC04084888 | Non-Substrate/Inhibitor | Non-Substrate/Inhibitor | Substrate/Inhibitor | Inhibitor | Inhibitor | High | |
|  | ZINC04084890 | Non-Substrate/Inhibitor | Non-Substrate/Inhibitor | Substrate/Inhibitor | Inhibitor | Inhibitor | High | |
|  | ZINC04085127 | Non-Substrate/Non-Inhibitor | Non-Substrate/Non-Inhibitor | Substrate/Inhibitor | Inhibitor | Inhibitor | High | |
|  | ZINC04085162 | Non-Substrate/Inhibitor | Non-Substrate/Non-Inhibitor | Substrate/Inhibitor | Inhibitor | Inhibitor | High | |
|  | ZINC04085562 | Non-Substrate/Non-Inhibitor | Non-Substrate/Non-Inhibitor | Substrate/Inhibitor | Inhibitor | Inhibitor | High | |
|  | ZINC04086945 | Non-Substrate/Non-Inhibitor | Non-Substrate/Non-Inhibitor | Substrate/Inhibitor | Inhibitor | Non-Inhibitor | High | |
|  | **ZINC04090179** | Non-Substrate/Non-Inhibitor | Non-Substrate/Non-Inhibitor | Substrate/Non-Inhibitor | Inhibitor | Non-Inhibitor | Low | |
|  | ZINC04090180 | Non-Substrate/Non-Inhibitor | Non-Substrate/Non-Inhibitor | Substrate/Non-Inhibitor | Inhibitor | Non-Inhibitor | Low | |
|  | **ZINC04090428** | Non-Substrate/Non-Inhibitor | Non-Substrate/Non-Inhibitor | Substrate/Non-Inhibitor | Inhibitor | Inhibitor | Low | |
|  | ZINC04235972 | Non-Substrate/Non-Inhibitor | Non-Substrate/Non-Inhibitor | Non-Substrate/Inhibitor | Non-Inhibitor | Non-Inhibitor | Low | |
|  | ZINC04236421 | Non-Substrate/Non-Inhibitor | Non-Substrate/Non-Inhibitor | Substrate/Inhibitor | Non-Inhibitor | Inhibitor | Low | |
|  | ZINC04237100 | Non-Substrate/Non-Inhibitor | Non-Substrate/Non-Inhibitor | Substrate/Non-Inhibitor | Non-Inhibitor | Non-Inhibitor | Low | |
|  | ZINC04258893 | Non-Substrate/Inhibitor | Non-Substrate/Non-Inhibitor | Substrate/Non-Inhibitor | Non-Inhibitor | Inhibitor | High | |
|  | ZINC04258903 | Non-Substrate/Inhibitor | Non-Substrate/Non-Inhibitor | Substrate/Non-Inhibitor | Non-Inhibitor | Inhibitor | High | |
|  | ZINC04259264 | Non-Substrate/Non-Inhibitor | Non-Substrate/Non-Inhibitor | Substrate/Non-Inhibitor | Non-Inhibitor | Non-Inhibitor | High | |
|  | ZINC04655273 | Non-Substrate/Non-Inhibitor | Non-Substrate/Non-Inhibitor | Substrate/Non-Inhibitor | Non-Inhibitor | Non-Inhibitor | Low | |
|  | **ZINC05220992** | Non-Substrate/Non-Inhibitor | Non-Substrate/Non-Inhibitor | Non-Substrate/Non-Inhibitor | Inhibitor | Non-Inhibitor | Low | |
|  | ZINC05397050 | Non-Substrate/Inhibitor | Non-Substrate/Non-Inhibitor | Substrate/Non-Inhibitor | Inhibitor | Non-Inhibitor | High | |
|  | ZINC05415240 | Non-Substrate/Non-Inhibitor | Non-Substrate/Non-Inhibitor | Non-Substrate/Non-Inhibitor | Non-Inhibitor | Non-Inhibitor | Low | |
|  | ZINC06092274 | Non-Substrate/Non-Inhibitor | Non-Substrate/Non-Inhibitor | Non-Substrate/Non-Inhibitor | Non-Inhibitor | Non-Inhibitor | Low | |
|  | ZINC06137732 | Non-Substrate/Non-Inhibitor | Non-Substrate/Non-Inhibitor | Substrate/Non-Inhibitor | Non-Inhibitor | Inhibitor | High | |
|  | ZINC06624582 | Non-Substrate/Non-Inhibitor | Non-Substrate/Non-Inhibitor | Substrate/Non-Inhibitor | Inhibitor | Non-Inhibitor | High | |
|  | ZINC06624588 | Non-Substrate/Non-Inhibitor | Substrate/Non-Inhibitor | Substrate/Non-Inhibitor | Inhibitor | Inhibitor | High | |
|  | ZINC06624612 | Non-Substrate/Non-Inhibitor | Non-Substrate/Non-Inhibitor | Non-Substrate/Non-Inhibitor | Inhibitor | Non-Inhibitor | High | |
|  | ZINC08299978 | Non-Substrate/Non-Inhibitor | Non-Substrate/Non-Inhibitor | Substrate/Non-Inhibitor | Non-Inhibitor | Non-Inhibitor | Low | |
|  | ZINC08382456 | Non-Substrate/Inhibitor | Non-Substrate/Inhibitor | Substrate/Inhibitor | Inhibitor | Inhibitor | High | |
|  | ZINC08445572 | Non-Substrate/Non-Inhibitor | Non-Substrate/Non-Inhibitor | Substrate/Non-Inhibitor | Non-Inhibitor | Non-Inhibitor | Low | |
|  | ZINC08649676 | Non-Substrate/Inhibitor | Non-Substrate/Inhibitor | Non-Substrate/Inhibitor | Inhibitor | Inhibitor | High | |
|  | ZINC08739084 | Non-Substrate/Non-Inhibitor | Non-Substrate/Non-Inhibitor | Substrate/Non-Inhibitor | Non-Inhibitor | Non-Inhibitor | Low | |
|  | ZINC08764694 | Non-Substrate/Non-Inhibitor | Non-Substrate/Non-Inhibitor | Substrate/Non-Inhibitor | Non-Inhibitor | Non-Inhibitor | Low | |
|  | ZINC08765261 | Non-Substrate/Non-Inhibitor | Non-Substrate/Non-Inhibitor | Substrate/Non-Inhibitor | Non-Inhibitor | Inhibitor | Low | |
|  | ZINC08790130 | Non-Substrate/Non-Inhibitor | Non-Substrate/Non-Inhibitor | Substrate/Inhibitor | Non-Inhibitor | Non-Inhibitor | High | |
|  | ZINC08791465 | Non-Substrate/Non-Inhibitor | Non-Substrate/Non-Inhibitor | Substrate/ Non-Inhibitor | Non-Inhibitor | Non-Inhibitor | High | |
|  | ZINC08791487 | Non-Substrate/Non-Inhibitor | Non-Substrate/Non-Inhibitor | Substrate/ Non-Inhibitor | Non-Inhibitor | Non-Inhibitor | Low | |
|  | ZINC08791488 | Non-Substrate/Non-Inhibitor | Non-Substrate/Non-Inhibitor | Substrate/ Non-Inhibitor | Non-Inhibitor | Non-Inhibitor | Low | |
|  | ZINC08791610 | Non-Substrate/Non-Inhibitor | Non-Substrate/Non-Inhibitor | Substrate/ Inhibitor | Non-Inhibitor | Non-Inhibitor | High | |
|  | ZINC08792167 | Non-Substrate/Non-Inhibitor | Non-Substrate/Non-Inhibitor | Substrate/ Non-Inhibitor | Inhibitor | Inhibitor | High | |
|  | ZINC08792170 | Non-Substrate/Non-Inhibitor | Non-Substrate/Non-Inhibitor | Substrate/ Non-Inhibitor | Non-Inhibitor | Non-Inhibitor | High | |
|  | ZINC08792350 | Non-Substrate/Inhibitor | Non-Substrate/Non-Inhibitor | Substrate/ Inhibitor | Non-Inhibitor | Inhibitor | High | |
|  | ZINC08792429 | Non-Substrate/Non-Inhibitor | Non-Substrate/Non-Inhibitor | Substrate/ Non-Inhibitor | Inhibitor | Non-Inhibitor | High | |
|  | ZINC08792433 | Non-Substrate/Inhibitor | Non-Substrate/Inhibitor | Substrate/ Inhibitor | Inhibitor | Inhibitor | High | |
|  | ZINC08877830 | Non-Substrate/Inhibitor | Non-Substrate/Non-Inhibitor | Substrate/ Inhibitor | Inhibitor | Non-Inhibitor | High | |
|  | ZINC08877855 | Non-Substrate/Inhibitor | Non-Substrate/Non-Inhibitor | Substrate/ Inhibitor | Inhibitor | Non-Inhibitor | High | |
|  | ZINC08877857 | Non-Substrate/Inhibitor | Non-Substrate/Non-Inhibitor | Non-Substrate/Inhibitor | Inhibitor | Non-Inhibitor | High | |
|  | ZINC08878911 | Non-Substrate/Non-Inhibitor | Non-Substrate/Non-Inhibitor | Substrate/ Non-Inhibitor | Non-Inhibitor | Non-Inhibitor | Low | |
|  | ZINC08878978 | Non-Substrate/Non-Inhibitor | Non-Substrate/Non-Inhibitor | Substrate/ Non-Inhibitor | Non-Inhibitor | Non-Inhibitor | Low | |
|  | ZINC08918127 | Non-Substrate/Non-Inhibitor | Non-Substrate/Inhibitor | Substrate/ Inhibitor | Non-Inhibitor | Inhibitor | Low | |
|  | ZINC08918259 | Non-Substrate/Non-Inhibitor | Non-Substrate/Inhibitor | Non-Substrate/Non-Inhibitor | Non-Inhibitor | Non-Inhibitor | Low | |
|  | **ZINC08918302** | Non-Substrate/Non-Inhibitor | Non-Substrate/Non-Inhibitor | Non-Substrate/Non-Inhibitor | Non-Inhibitor | Non-Inhibitor | Low | |
|  | ZINC08918345 | Non-Substrate/Non-Inhibitor | Non-Substrate/Non-Inhibitor | Substrate/Non-Inhibitor | Non-Inhibitor | Non-Inhibitor | Low | |
|  | ZINC08991637 | Non-Substrate/Non-Inhibitor | Non-Substrate/Non-Inhibitor | Substrate/ Inhibitor | Non-Inhibitor | Inhibitor | High | |
|  | ZINC08993096 | Non-Substrate/Non-Inhibitor | Non-Substrate/Non-Inhibitor | Non-Substrate/Inhibitor | Inhibitor | Non-Inhibitor | High | |
|  | ZINC09034003 | Non-Substrate/Non-Inhibitor | Non-Substrate/Non-Inhibitor | Substrate/Inhibitor | Non-Inhibitor | Non-Inhibitor | Low | |
|  | ZINC09034064 | Non-Substrate/Inhibitor | Non-Substrate/Non-Inhibitor | Substrate/Inhibitor | Non-Inhibitor | Inhibitor | High | |
|  | ZINC09054134 | Non-Substrate/Non-Inhibitor | Non-Substrate/Inhibitor | Substrate/Inhibitor | Inhibitor | Non-Inhibitor | High | |
|  | ZINC09089656 | Non-Substrate/Non-Inhibitor | Non-Substrate/Inhibitor | Substrate/Non-Inhibitor | Non-Inhibitor | Inhibitor | Low | |
|  | **ZINC09303152** | Non-Substrate/Non-Inhibitor | Non-Substrate/Non-Inhibitor | Substrate/ Non-Inhibitor | Non-Inhibitor | Non-Inhibitor | Low | |
|  | ZINC09341662 | Non-Substrate/Non-Inhibitor | Non-Substrate/Non-Inhibitor | Substrate/Inhibitor | Non-Inhibitor | Inhibitor | High | |
|  | ZINC09420846 | Non-Substrate/Non-Inhibitor | Non-Substrate/Non-Inhibitor | Non-Substrate/Inhibitor | Inhibitor | Non-Inhibitor | High | |
|  | ZINC09423653 | Non-Substrate/Non-Inhibitor | Non-Substrate/Non-Inhibitor | Non-Substrate/Inhibitor | Inhibitor | Non-Inhibitor | High | |
|  | ZINC09423883 | Non-Substrate/Non-Inhibitor | Non-Substrate/Non-Inhibitor | Substrate/Inhibitor | Inhibitor | Non-Inhibitor | High | |
|  | ZINC09423968 | Non-Substrate/Non-Inhibitor | Non-Substrate/Non-Inhibitor | Non-Substrate/Inhibitor | Inhibitor | Non-Inhibitor | High | |
|  | ZINC09576136 | Non-Substrate/Inhibitor | Non-Substrate/Non-Inhibitor | Substrate/Inhibitor | Non-Inhibitor | Inhibitor | High | |
|  | ZINC11865187 | Non-Substrate/Inhibitor | Non-Substrate/Non-Inhibitor | Substrate/Non-Inhibitor | Inhibitor | Inhibitor | High | |
|  | ZINC11865349 | Non-Substrate/Non-Inhibitor | Non-Substrate/Non-Inhibitor | Substrate/ Non-Inhibitor | Non-Inhibitor | Non-Inhibitor | High | |
|  | ZINC11865376 | Non-Substrate/Non-Inhibitor | Non-Substrate/Non-Inhibitor | Non-Substrate/Non-Inhibitor | Inhibitor | Inhibitor | High | |
|  | ZINC11866483 | Non-Substrate/Non-Inhibitor | Non-Substrate/Non-Inhibitor | Substrate/Non-Inhibitor | Inhibitor | Non-Inhibitor | Low | |
|  | ZINC11866484 | Non-Substrate/Non-Inhibitor | Non-Substrate/Non-Inhibitor | Substrate/Non-Inhibitor | Inhibitor | Non-Inhibitor | Low | |
|  | ZINC11866485 | Non-Substrate/Non-Inhibitor | Non-Substrate/Non-Inhibitor | Substrate/Non-Inhibitor | Inhibitor | Non-Inhibitor | Low | |
|  | ZINC11866495 | Non-Substrate/Non-Inhibitor | Non-Substrate/Non-Inhibitor | Substrate/Non-Inhibitor | Inhibitor | Non-Inhibitor | Low | |
|  | ZINC11867066 | Non-Substrate/Non-Inhibitor | Non-Substrate/Non-Inhibitor | Substrate/Inhibitor | Inhibitor | Non-Inhibitor | Low | |
|  | ZINC11867457 | Non-Substrate/Inhibitor | Non-Substrate/Non-Inhibitor | Substrate/Inhibitor | Inhibitor | Non-Inhibitor | High | |
|  | ZINC11867458 | Non-Substrate/Inhibitor | Non-Substrate/Non-Inhibitor | Substrate/Inhibitor | Inhibitor | Non-Inhibitor | High | |
|  | ZINC11867461 | Non-Substrate/Non-Inhibitor | Non-Substrate/Non-Inhibitor | Substrate/Non-Inhibitor | Inhibitor | Non-Inhibitor | Low | |
|  | ZINC11867462 | Non-Substrate/Non-Inhibitor | Non-Substrate/Non-Inhibitor | Substrate/Non-Inhibitor | Inhibitor | Non-Inhibitor | Low | |
|  | ZINC11867465 | Non-Substrate/Inhibitor | Non-Substrate/Non-Inhibitor | Substrate/Inhibitor | Inhibitor | Inhibitor | High | |
|  | ZINC11867466 | Non-Substrate/Inhibitor | Non-Substrate/Non-Inhibitor | Substrate/Inhibitor | Inhibitor | Inhibitor | High | |
|  | ZINC12296404 | Non-Substrate/Non-Inhibitor | Non-Substrate/Non-Inhibitor | Substrate/Non-Inhibitor | Non-Inhibitor | Non-Inhibitor | Low | |
|  | ZINC12296477 | Non-Substrate/Inhibitor | Non-Substrate/Non-Inhibitor | Substrate/Inhibitor | Inhibitor | Inhibitor | High | |
|  | ZINC12296478 | Non-Substrate/Inhibitor | Non-Substrate/Non-Inhibitor | Substrate/Inhibitor | Inhibitor | Inhibitor | High | |
|  | ZINC12296500 | Non-Substrate/Non-Inhibitor | Non-Substrate/Non-Inhibitor | Substrate/Non-Inhibitor | Inhibitor | Non-Inhibitor | Low | |
|  | ZINC12296501 | Non-Substrate/Non-Inhibitor | Non-Substrate/Non-Inhibitor | Substrate/Non-Inhibitor | Inhibitor | Non-Inhibitor | Low | |
|  | ZINC12296551 | Non-Substrate/Inhibitor | Non-Substrate/Non-Inhibitor | Substrate/Non-Inhibitor | Inhibitor | Non-Inhibitor | High | |
|  | ZINC12296564 | Non-Substrate/Inhibitor | Non-Substrate/Non-Inhibitor | Substrate/Inhibitor | Inhibitor | Inhibitor | High | |
|  | ZINC12296565 | Non-Substrate/Inhibitor | Non-Substrate/Non-Inhibitor | Substrate/Inhibitor | Inhibitor | Inhibitor | High | |
|  | **ZINC12296580** | Non-Substrate/Non-Inhibitor | Non-Substrate/Non-Inhibitor | Substrate/Inhibitor | Inhibitor | Inhibitor | Low | |
|  | ZINC12296581 | Non-Substrate/Non-Inhibitor | Non-Substrate/Non-Inhibitor | Substrate/Inhibitor | Inhibitor | Inhibitor | Low | |
|  | ZINC12296700 | Non-Substrate/Non-Inhibitor | Non-Substrate/Non-Inhibitor | Substrate/Non-Inhibitor | Inhibitor | Non-Inhibitor | Low | |
|  | ZINC12296701 | Non-Substrate/Non-Inhibitor | Non-Substrate/Non-Inhibitor | Substrate/Non-Inhibitor | Inhibitor | Non-Inhibitor | Low | |
|  | ZINC12296761 | Non-Substrate/Non-Inhibitor | Non-Substrate/Non-Inhibitor | Substrate/Non-Inhibitor | Inhibitor | Non-Inhibitor | Low | |
|  | ZINC12296762 | Non-Substrate/Non-Inhibitor | Non-Substrate/Non-Inhibitor | Substrate/Non-Inhibitor | Inhibitor | Non-Inhibitor | Low | |
|  | ZINC12296864 | Non-Substrate/Inhibitor | Non-Substrate/Non-Inhibitor | Substrate/Non-Inhibitor | Inhibitor | Non-Inhibitor | High | |
|  | ZINC12296876 | Non-Substrate/Non-Inhibitor | Non-Substrate/Non-Inhibitor | Substrate/Non-Inhibitor | Inhibitor | Non-Inhibitor | Low | |
|  | ZINC12297013 | Non-Substrate/Non-Inhibitor | Non-Substrate/Non-Inhibitor | Substrate/Non-Inhibitor | Inhibitor | Non-Inhibitor | Low | |
|  | ZINC12297014 | Non-Substrate/Non-Inhibitor | Non-Substrate/Non-Inhibitor | Substrate/Non-Inhibitor | Inhibitor | Non-Inhibitor | Low | |
|  | ZINC12297019 | Non-Substrate/Non-Inhibitor | Substrate/Inhibitor | Substrate/Non-Inhibitor | Non-Inhibitor | Non-Inhibitor | High | |
|  | **ZINC12297025** | Non-Substrate/Non-Inhibitor | Non-Substrate/Non-Inhibitor | Non-Substrate/Non-Inhibitor | Non-Inhibitor | Non-Inhibitor | Low | |
|  | ZINC12297042 | Non-Substrate/Non-Inhibitor | Non-Substrate/Non-Inhibitor | Substrate/Inhibitor | Inhibitor | Non-Inhibitor | High | |
|  | ZINC12297091 | Non-Substrate/Non-Inhibitor | Non-Substrate/Non-Inhibitor | Substrate/Non-Inhibitor | Non-Inhibitor | Non-Inhibitor | Low | |
|  | ZINC12297092 | Non-Substrate/Non-Inhibitor | Non-Substrate/Non-Inhibitor | Substrate/Non-Inhibitor | Non-Inhibitor | Non-Inhibitor | Low | |
|  | ZINC12442585 | Non-Substrate/Inhibitor | Non-Substrate/Non-Inhibitor | Substrate/Inhibitor | Non-Inhibitor | Inhibitor | High | |
|  | ZINC12442587 | Non-Substrate/Inhibitor | Non-Substrate/Non-Inhibitor | Substrate/Inhibitor | Non-Inhibitor | Inhibitor | High | |
|  | ZINC12442588 | Non-Substrate/Inhibitor | Non-Substrate/Non-Inhibitor | Substrate/Inhibitor | Non-Inhibitor | Inhibitor | High | |
|  | ZINC12442590 | Non-Substrate/Inhibitor | Non-Substrate/Non-Inhibitor | Substrate/Inhibitor | Non-Inhibitor | Inhibitor | High | |
|  | ZINC12442592 | Non-Substrate/Inhibitor | Non-Substrate/Non-Inhibitor | Substrate/Inhibitor | Non-Inhibitor | Inhibitor | High | |
|  | ZINC12442594 | Non-Substrate/Inhibitor | Non-Substrate/Non-Inhibitor | Substrate/Inhibitor | Non-Inhibitor | Inhibitor | High | |
|  | ZINC12442597 | Non-Substrate/Inhibitor | Non-Substrate/Non-Inhibitor | Substrate/Inhibitor | Non-Inhibitor | Inhibitor | High | |
|  | ZINC12442599 | Non-Substrate/Inhibitor | Non-Substrate/Non-Inhibitor | Substrate/Inhibitor | Non-Inhibitor | Inhibitor | High | |
|  | ZINC12442608 | Non-Substrate/Inhibitor | Non-Substrate/Non-Inhibitor | Substrate/Inhibitor | Non-Inhibitor | Inhibitor | High | |
|  | ZINC12442610 | Non-Substrate/Inhibitor | Non-Substrate/Non-Inhibitor | Substrate/Inhibitor | Non-Inhibitor | Inhibitor | High | |
|  | ZINC12442649 | Non-Substrate/Non-Inhibitor | Non-Substrate/Non-Inhibitor | Substrate/Non-Inhibitor | Inhibitor | Non-Inhibitor | High | |
|  | ZINC12442671 | Non-Substrate/Non-Inhibitor | Non-Substrate/Non-Inhibitor | Substrate/Inhibitor | Inhibitor | Inhibitor | High | |
|  | ZINC12602816 | Non-Substrate/Non-Inhibitor | Non-Substrate/Non-Inhibitor | Substrate/Non-Inhibitor | Non-Inhibitor | Non-Inhibitor | Low | |
|  | ZINC12604806 | Non-Substrate/Non-Inhibitor | Non-Substrate/Non-Inhibitor | Substrate/Inhibitor | Non-Inhibitor | Inhibitor | High | |
|  | ZINC12660190 | Non-Substrate/Non-Inhibitor | Non-Substrate/Non-Inhibitor | Substrate/Non-Inhibitor | Non-Inhibitor | Non-Inhibitor | Low | |
|  | ZINC12863193 | Non-Substrate/Non-Inhibitor | Non-Substrate/Non-Inhibitor | Substrate/Non-Inhibitor | Non-Inhibitor | Inhibitor | Low | |
|  | ZINC12863203 | Non-Substrate/Non-Inhibitor | Non-Substrate/Non-Inhibitor | Substrate/Non-Inhibitor | Non-Inhibitor | Inhibitor | Low | |
|  | ZINC12866401 | Non-Substrate/Non-Inhibitor | Non-Substrate/Non-Inhibitor | Substrate/Non-Inhibitor | Non-Inhibitor | Non-Inhibitor | Low | |
|  | ZINC12870752 | Non-Substrate/Non-Inhibitor | Non-Substrate/Non-Inhibitor | Substrate/Non-Inhibitor | Non-Inhibitor | Non-Inhibitor | Low | |
|  | ZINC12887203 | Non-Substrate/Non-Inhibitor | Non-Substrate/Non-Inhibitor | Substrate/Non-Inhibitor | Non-Inhibitor | Non-Inhibitor | Low | |
|  | ZINC12892129 | Non-Substrate/Inhibitor | Non-Substrate/Non-Inhibitor | Substrate/Non-Inhibitor | Non-Inhibitor | Non-Inhibitor | High | |
|  | ZINC12893265 | Non-Substrate/Non-Inhibitor | Non-Substrate/Non-Inhibitor | Substrate/Non-Inhibitor | Inhibitor | Non-Inhibitor | Low | |
|  | ZINC12893333 | Non-Substrate/Non-Inhibitor | Non-Substrate/Non-Inhibitor | Substrate/Non-Inhibitor | Non-Inhibitor | Non-Inhibitor | Low | |
|  | ZINC12899484 | Non-Substrate/Inhibitor | Non-Substrate/Non-Inhibitor | Substrate/Non-Inhibitor | Inhibitor | Non-Inhibitor | High | |
|  | ZINC12899490 | Non-Substrate/Inhibitor | Non-Substrate/Non-Inhibitor | Substrate/Non-Inhibitor | Inhibitor | Non-Inhibitor | High | |
|  | ZINC13359662 | Non-Substrate/Non-Inhibitor | Non-Substrate/Non-Inhibitor | Substrate/Non-Inhibitor | Non-Inhibitor | Non-Inhibitor | Low | |
|  | ZINC13551971 | Non-Substrate/Non-Inhibitor | Non-Substrate/Non-Inhibitor | Substrate/Non-Inhibitor | Non-Inhibitor | Non-Inhibitor | Low | |
|  | ZINC13553109 | Non-Substrate/Non-Inhibitor | Non-Substrate/Non-Inhibitor | Substrate/Non-Inhibitor | Non-Inhibitor | Non-Inhibitor | Low | |
|  | ZINC13683907 | Non-Substrate/Non-Inhibitor | Non-Substrate/Non-Inhibitor | Substrate/Non-Inhibitor | Inhibitor | Non-Inhibitor | High | |
|  | ZINC13687938 | Non-Substrate/Non-Inhibitor | Non-Substrate/Non-Inhibitor | Substrate/ Inhibitor | Non-Inhibitor | Inhibitor | High | |
|  | ZINC13692664 | Non-Substrate/Non-Inhibitor | Non-Substrate/Non-Inhibitor | Substrate/ Inhibitor | Non-Inhibitor | Inhibitor | High | |
|  | ZINC13720979 | Non-Substrate/Non-Inhibitor | Non-Substrate/Non-Inhibitor | Substrate/ Inhibitor | Non-Inhibitor | Non-Inhibitor | High | |
|  | ZINC13721203 | Non-Substrate/Non-Inhibitor | Non-Substrate/Non-Inhibitor | Substrate/ Inhibitor | Inhibitor | Inhibitor | High | |
|  | ZINC13731223 | Non-Substrate/Non-Inhibitor | Non-Substrate/Non-Inhibitor | Substrate/ Inhibitor | Inhibitor | Inhibitor | High | |
|  | ZINC13732746 | Non-Substrate/Non-Inhibitor | Non-Substrate/Non-Inhibitor | Substrate/ Inhibitor | Non-Inhibitor | Non-Inhibitor | High | |
|  | ZINC13732895 | Non-Substrate/Non-Inhibitor | Non-Substrate/Non-Inhibitor | Substrate/ Inhibitor | Inhibitor | Non-Inhibitor | High | |
|  | ZINC13736331 | Non-Substrate/Non-Inhibitor | Non-Substrate/Non-Inhibitor | Substrate/ Inhibitor | Non-Inhibitor | Inhibitor | High | |
|  | ZINC15303561 | Non-Substrate/Non-Inhibitor | Non-Substrate/Non-Inhibitor | Non-Substrate/Inhibitor | Non-Inhibitor | Non-Inhibitor | High | |
|  | ZINC15303564 | Non-Substrate/Non-Inhibitor | Non-Substrate/Non-Inhibitor | Non-Substrate/Inhibitor | Non-Inhibitor | Non-Inhibitor | High | |
|  | ZINC15957613 | Non-Substrate/Inhibitor | Non-Substrate/Non-Inhibitor | Substrate/ Inhibitor | Non-Inhibitor | Non-Inhibitor | Low | |
|  | ZINC15959322 | Non-Substrate/Non-Inhibitor | Non-Substrate/Non-Inhibitor | Substrate/ Inhibitor | Non-Inhibitor | Non-Inhibitor | High | |
|  | ZINC15959323 | Non-Substrate/Non-Inhibitor | Non-Substrate/Non-Inhibitor | Substrate/ Inhibitor | Non-Inhibitor | Non-Inhibitor | High | |
|  | ZINC15959325 | Non-Substrate/Non-Inhibitor | Non-Substrate/Non-Inhibitor | Substrate/ Inhibitor | Non-Inhibitor | Non-Inhibitor | High | |
|  | ZINC15967689 | Non-Substrate/Non-Inhibitor | Non-Substrate/Non-Inhibitor | Substrate/ Inhibitor | Non-Inhibitor | Non-Inhibitor | High | |
|  | ZINC15967691 | Non-Substrate/Non-Inhibitor | Non-Substrate/Non-Inhibitor | Substrate/ Inhibitor | Non-Inhibitor | Non-Inhibitor | High | |
|  | ZINC15967694 | Non-Substrate/Non-Inhibitor | Non-Substrate/Non-Inhibitor | Substrate/ Inhibitor | Non-Inhibitor | Non-Inhibitor | High | |
|  | ZINC15969172 | Non-Substrate/Non-Inhibitor | Non-Substrate/Non-Inhibitor | Substrate/ Inhibitor | Non-Inhibitor | Non-Inhibitor | High | |
|  | ZINC15969174 | Non-Substrate/Non-Inhibitor | Non-Substrate/Non-Inhibitor | Substrate/ Inhibitor | Non-Inhibitor | Non-Inhibitor | High | |
|  | ZINC15969569 | Non-Substrate/Inhibitor | Non-Substrate/Non-Inhibitor | Substrate/ Inhibitor | Non-Inhibitor | Inhibitor | High | |
|  | ZINC15969571 | Non-Substrate/Inhibitor | Non-Substrate/Non-Inhibitor | Substrate/ Inhibitor | Non-Inhibitor | Inhibitor | High | |
|  | ZINC15969573 | Non-Substrate/Inhibitor | Non-Substrate/Non-Inhibitor | Substrate/ Inhibitor | Non-Inhibitor | Inhibitor | High | |
|  | ZINC16026354 | Non-Substrate/Non-Inhibitor | Non-Substrate/Non-Inhibitor | Substrate/ Inhibitor | Non-Inhibitor | Non-Inhibitor | High | |
|  | **ZINC16292828** | Non-Substrate/Non-Inhibitor | Non-Substrate/Non-Inhibitor | Substrate/Non-Inhibitor | Non-Inhibitor | Non-Inhibitor | Low | |
|  | ZINC16972169 | Non-Substrate/Non-Inhibitor | Non-Substrate/Non-Inhibitor | Non-Substrate/Non-Inhibitor | Non-Inhibitor | Non-Inhibitor | Low | |
|  | ZINC16995797 | Non-Substrate/Non-Inhibitor | Non-Substrate/Non-Inhibitor | Non-Substrate/Non-Inhibitor | Non-Inhibitor | Non-Inhibitor | Low | |
|  | ZINC17014720 | Non-Substrate/Non-Inhibitor | Non-Substrate/Non-Inhibitor | Substrate/Non-Inhibitor | Non-Inhibitor | Non-Inhibitor | Low | |
|  | ZINC17015252 | Non-Substrate/Non-Inhibitor | Non-Substrate/Non-Inhibitor | Substrate/Non-Inhibitor | Non-Inhibitor | Non-Inhibitor | Low | |
|  | ZINC17029354 | Non-Substrate/Non-Inhibitor | Non-Substrate/Non-Inhibitor | Substrate/Non-Inhibitor | Non-Inhibitor | Non-Inhibitor | Low | |
|  | ZINC17194529 | Non-Substrate/Non-Inhibitor | Non-Substrate/Non-Inhibitor | Non-Substrate/Non-Inhibitor | Non-Inhibitor | Non-Inhibitor | Low | |
|  | ZINC17195517 | Non-Substrate/Non-Inhibitor | Non-Substrate/Non-Inhibitor | Substrate/Non-Inhibitor | Non-Inhibitor | Non-Inhibitor | Low | |
|  | ZINC17196472 | Non-Substrate/Non-Inhibitor | Non-Substrate/Non-Inhibitor | Non-Substrate/Non-Inhibitor | Non-Inhibitor | Non-Inhibitor | Low | |
|  | ZINC17196754 | Non-Substrate/Non-Inhibitor | Non-Substrate/Non-Inhibitor | Substrate/Non-Inhibitor | Non-Inhibitor | Non-Inhibitor | Low | |
|  | ZINC18091170 | Non-Substrate/Non-Inhibitor | Non-Substrate/Non-Inhibitor | Substrate/Non-Inhibitor | Non-Inhibitor | Non-Inhibitor | Low | |
|  | ZINC18176990 | Non-Substrate/Non-Inhibitor | Non-Substrate/Non-Inhibitor | Substrate/Non-Inhibitor | Non-Inhibitor | Non-Inhibitor | High | |
|  | ZINC18207570 | Non-Substrate/Non-Inhibitor | Non-Substrate/Non-Inhibitor | Non-Substrate/Non-Inhibitor | Non-Inhibitor | Non-Inhibitor | Low |  |
|  | ZINC18219349 | Non-Substrate/Non-Inhibitor | Non-Substrate/Non-Inhibitor | Substrate/Non-Inhibitor | Non-Inhibitor | Non-Inhibitor | Low |  |
|  | ZINC19891473 | Non-Substrate/Non-Inhibitor | Non-Substrate/Non-Inhibitor | Substrate/ Inhibitor | Non-Inhibitor | Non-Inhibitor | High |  |
|  | ZINC20411267 | Non-Substrate/Non-Inhibitor | Non-Substrate/Non-Inhibitor | Substrate/Non-Inhibitor | Non-Inhibitor | Non-Inhibitor | Low |  |
|  | ZINC20411826 | Non-Substrate/Non-Inhibitor | Non-Substrate/Non-Inhibitor | Non-Substrate/Non-Inhibitor | Non-Inhibitor | Non-Inhibitor | Low |  |
|  | ZINC20412340 | Non-Substrate/Non-Inhibitor | Non-Substrate/Non-Inhibitor | Substrate/Non-Inhibitor | Non-Inhibitor | Non-Inhibitor | Low |  |
|  | ZINC20611122 | Non-Substrate/Non-Inhibitor | Non-Substrate/Non-Inhibitor | Substrate/Non-Inhibitor | Non-Inhibitor | Non-Inhibitor | High |  |
|  | ZINC31169877 | Non-Substrate/Non-Inhibitor | Non-Substrate/Non-Inhibitor | Substrate/Non-Inhibitor | Non-Inhibitor | Non-Inhibitor | Low |  |
|  | ZINC32502304 | Non-Substrate/Non-Inhibitor | Non-Substrate/Non-Inhibitor | Substrate/Non-Inhibitor | Non-Inhibitor | Non-Inhibitor | Low |  |
|  | ZINC32502306 | Non-Substrate/Non-Inhibitor | Non-Substrate/Non-Inhibitor | Substrate/ Inhibitor | Non-Inhibitor | Non-Inhibitor | High |  |
|  | ZINC34300440 | Non-Substrate/Non-Inhibitor | Non-Substrate/Non-Inhibitor | Substrate/ Inhibitor | Inhibitor | Non-Inhibitor | Low |  |
|  | ZINC35365995 | Non-Substrate/Non-Inhibitor | Non-Substrate/Non-Inhibitor | Substrate/Non-Inhibitor | Non-Inhibitor | Non-Inhibitor | High |  |
|  | ZINC35365997 | Non-Substrate/Non-Inhibitor | Non-Substrate/Non-Inhibitor | Substrate/Non-Inhibitor | Non-Inhibitor | Non-Inhibitor | High |  |
|  | ZINC35457807 | Non-Substrate/Non-Inhibitor | Non-Substrate/Non-Inhibitor | Substrate/Non-Inhibitor | Non-Inhibitor | Non-Inhibitor | Low |  |
|  | ZINC36369885 | Non-Substrate/Inhibitor | Non-Substrate/Non-Inhibitor | Substrate/Non-Inhibitor | Inhibitor | Inhibitor | High |  |
|  | ZINC38167070 | Non-Substrate/Inhibitor | Non-Substrate/Non-Inhibitor | Non-Substrate/Non-Inhibitor | Non-Inhibitor | Non-Inhibitor | High |  |
|  | ZINC38167083 | Non-Substrate/Inhibitor | Non-Substrate/Non-Inhibitor | Non-Substrate/Inhibitor | Inhibitor | Non-Inhibitor | High |  |
|  | ZINC54278174 | Non-Substrate/Non-Inhibitor | Non-Substrate/Inhibitor | Non-Substrate/Inhibitor | Inhibitor | Non-Inhibitor | High |  |
|  | ZINC54278181 | Non-Substrate/Non-Inhibitor | Non-Substrate/Inhibitor | Substrate/Inhibitor | Inhibitor | Non-Inhibitor | High |  |
|  | ZINC54278191 | Non-Substrate/Non-Inhibitor | Non-Substrate/Non-Inhibitor | Non-Substrate/Inhibitor | Inhibitor | Non-Inhibitor | High |  |
|  | ZINC54333105 | Non-Substrate/Non-Inhibitor | Non-Substrate/Non-Inhibitor | Substrate/Inhibitor | Non-Inhibitor | Inhibitor | High |  |
|  | ZINC54333106 | Non-Substrate/Non-Inhibitor | Non-Substrate/Non-Inhibitor | Substrate/Inhibitor | Non-Inhibitor | Inhibitor | High |  |
|  | ZINC55396209 | Non-Substrate/Non-Inhibitor | Non-Substrate/Inhibitor | Substrate/Inhibitor | Inhibitor | Inhibitor | High |  |
|  | ZINC58090636 | Non-Substrate/Non-Inhibitor | Non-Substrate/Inhibitor | Substrate/Inhibitor | Inhibitor | Non-Inhibitor | High |  |
|  | ZINC61997665 | Non-Substrate/Non-Inhibitor | Non-Substrate/Non-Inhibitor | Substrate/Inhibitor | Non-Inhibitor | Inhibitor | High |  |
|  | ZINC61997666 | Non-Substrate/Non-Inhibitor | Non-Substrate/Non-Inhibitor | Substrate/Inhibitor | Non-Inhibitor | Inhibitor | High |  |
|  | ZINC61997705 | Non-Substrate/Non-Inhibitor | Non-Substrate/Non-Inhibitor | Non-Substrate/Inhibitor | Inhibitor | Non-Inhibitor | High |  |
|  | ZINC62001268 | Non-Substrate/Non-Inhibitor | Non-Substrate/Non-Inhibitor | Substrate/Non-Inhibitor | Non-Inhibitor | Non-Inhibitor | High |  |
|  | ZINC67913130 | Non-Substrate/Non-Inhibitor | Non-Substrate/Non-Inhibitor | Substrate/Non-Inhibitor | Non-Inhibitor | Non-Inhibitor | Low |  |
|  | ZINC68569286 | Non-Substrate/Inhibitor | Non-Substrate/Non-Inhibitor | Non-Substrate/Inhibitor | Inhibitor | Inhibitor | High |  |
|  | **ZINC68569602** | Non-Substrate/Non-Inhibitor | Non-Substrate/Non-Inhibitor | Non-Substrate/Non-Inhibitor | Non-Inhibitor | Non-Inhibitor | Low |  |
|  | ZINC68572120 | Non-Substrate/Non-Inhibitor | Non-Substrate/Inhibitor | Substrate/Inhibitor | Inhibitor | Inhibitor | High |  |
|  | **ZINC68583170** | Non-Substrate/Non-Inhibitor | Non-Substrate/Non-Inhibitor | Substrate/Non-Inhibitor | Non-Inhibitor | Non-Inhibitor | Low |  |
|  | ZINC68583182 | Non-Substrate/Non-Inhibitor | Non-Substrate/Non-Inhibitor | Substrate/Non-Inhibitor | Non-Inhibitor | Inhibitor | Low |  |
|  | ZINC68589458 | Non-Substrate/Non-Inhibitor | Non-Substrate/Non-Inhibitor | Substrate/Inhibitor | Non-Inhibitor | Non-Inhibitor | Low |  |
|  | ZINC68589462 | Non-Substrate/Non-Inhibitor | Non-Substrate/Non-Inhibitor | Substrate/Inhibitor | Non-Inhibitor | Non-Inhibitor | Low |  |
|  | ZINC68591854 | Non-Substrate/Inhibitor | Non-Substrate/Non-Inhibitor | Substrate/Non-Inhibitor | Non-Inhibitor | Non-Inhibitor | Low |  |
|  | ZINC68591857 | Non-Substrate/Inhibitor | Non-Substrate/Non-Inhibitor | Substrate/Non-Inhibitor | Non-Inhibitor | Non-Inhibitor | Low |  |

**Supplementary Table S4.** *in-silico* toxicity, carcinogenicity and LD_50_ profile obtained from admetSAR server for selected 330 compounds from virtual screening. Selected compounds (14) for re-docking were highlighted in bold.

| **Sr. No.** | **ZINC ID** | **AMES Toxicity** | **Carcinogen** | | **hERG inhibition** | **Acute Oral Toxicity** | **Rat LD_50_** |
| --- | --- | --- | --- | --- | --- | --- | --- |
|  | ZINC00120631 | Non-Toxic | Non-carcinogens | | Non-Inhibitor | II/0.5510 | 2.5625 |
|  | ZINC00238468 | Non-Toxic | Non-carcinogens | | Non-Inhibitor | III/0.6499 | 1.8609 |
|  | ZINC00338371 | Non-Toxic | Non-carcinogens | | Non-Inhibitor | II/0.5243 | 2.5699 |
|  | ZINC00408721 | Non-Toxic | Non-carcinogens | | Non-Inhibitor | III/0.4172 | 2.2581 |
|  | ZINC00490486 | Non-Toxic | Non-carcinogens | | Non-Inhibitor | III/0.4899 | 2.4044 |
|  | ZINC00519086 | Non-Toxic | Non-carcinogens | | Non-Inhibitor | III/0.6499 | 1.8609 |
|  | ZINC01482030 | Non-Toxic | Non-carcinogens | | Non-Inhibitor | III/0.5844 | 2.5300 |
|  | ZINC01686586 | Toxic | Non-carcinogens | | Non-Inhibitor | II/0.7364 | 2.8888 |
|  | ZINC01726969 | Toxic | Non-carcinogens | | Non-Inhibitor | II/0.6421 | 2.6152 |
|  | ZINC01787663 | Non-Toxic | Non-carcinogens | | Non-Inhibitor | II/0.4466 | 2.4312 |
|  | ZINC01787672 | Non-Toxic | Non-carcinogens | | Non-Inhibitor | II/0.4466 | 2.4312 |
|  | ZINC01845382 | Toxic | Non-carcinogens | | Inhibitor | III/0.5688 | 2.6170 |
|  | ZINC01886047 | Toxic | Non-carcinogens | | Inhibitor | III/0.6457 | 2.5892 |
|  | ZINC02001186 | Toxic | Non-carcinogens | | Non-Inhibitor | III/0.3561 | 2.6544 |
|  | ZINC02092089 | Non-Toxic | Non-carcinogens | | Inhibitor | III/0.5520 | 2.6396 |
|  | ZINC02092130 | Non-Toxic | Non-carcinogens | | Inhibitor | III/0.6255 | 2.7164 |
|  | ZINC02092414 | Toxic | Non-carcinogens | | Non-Inhibitor | III/0.6530 | 2.5759 |
|  | ZINC02092422 | Non-Toxic | Non-carcinogens | | Inhibitor | III/0.6645 | 2.6835 |
|  | ZINC02092475 | Non-Toxic | Non-carcinogens | | Inhibitor | III/0.5520 | 2.6396 |
|  | ZINC02092931 | Non-Toxic | Non-carcinogens | | Non-Inhibitor | III/0.6214 | 2.3639 |
|  | ZINC02093637 | Non-Toxic | Non-carcinogens | | Inhibitor | III/0.5688 | 2.6170 |
|  | ZINC02094632 | Non-Toxic | Non-carcinogens | | Inhibitor | III/0.6255 | 2.7164 |
|  | ZINC02095117 | Non-Toxic | Non-carcinogens | | Inhibitor | III/0.5427 | 2.7301 |
|  | ZINC02095140 | Non-Toxic | Non-carcinogens | | Inhibitor | III/0.6334 | 2.6274 |
|  | ZINC02095426 | Non-Toxic | Non-carcinogens | | Inhibitor | III/0.5903 | 2.7267 |
|  | ZINC02096038 | Non-Toxic | Non-carcinogens | | Inhibitor | III/0.5388 | 2.6865 |
|  | ZINC02096725 | Non-Toxic | Non-carcinogens | | Inhibitor | III/0.6485 | 2.5635 |
|  | ZINC02098157 | Non-Toxic | Non-carcinogens | | Inhibitor | III/0.6699 | 2.6238 |
|  | ZINC02098972 | Non-Toxic | Non-carcinogens | | Inhibitor | III/0.5662 | 2.6655 |
|  | ZINC02100762 | Non-Toxic | Non-carcinogens | | Non-Inhibitor | III/0.6945 | 2.5340 |
|  | ZINC02101594 | Non-Toxic | Non-carcinogens | | Inhibitor | III/0.6578 | 2.6805 |
|  | ZINC02101693 | Non-Toxic | Non-carcinogens | | Non-Inhibitor | III/0.6496 | 2.7324 |
|  | ZINC02101695 | Non-Toxic | Non-carcinogens | | Non-Inhibitor | III/0.6496 | 2.7324 |
|  | ZINC02102083 | Non-Toxic | Non-carcinogens | | Non-Inhibitor | III/0.6351 | 2.1841 |
|  | ZINC02102560 | Non-Toxic | Non-carcinogens | | Inhibitor | III/0.6255 | 2.7164 |
|  | ZINC02103115 | Non-Toxic | Non-carcinogens | | Non-Inhibitor | III/0.6960 | 2.3401 |
|  | ZINC02103122 | Non-Toxic | Non-carcinogens | | Inhibitor | III/0.4908 | 2.5947 |
|  | ZINC02103383 | Non-Toxic | Non-carcinogens | | Inhibitor | III/0.6651 | 2.6994 |
|  | ZINC02103425 | Non-Toxic | Non-carcinogens | | Inhibitor | III/0.5427 | 2.7301 |
|  | ZINC02103558 | Non-Toxic | Non-carcinogens | | Inhibitor | III/0.6699 | 2.6238 |
|  | ZINC02103644 | Non-Toxic | Non-carcinogens | | Inhibitor | III/0.6642 | 2.6134 |
|  | ZINC02104482 | Non-Toxic | Non-carcinogens | | Non-Inhibitor | III/0.6018 | 2.4426 |
|  | ZINC02105209 | Non-Toxic | Non-carcinogens | | Non-Inhibitor | III/0.6310 | 2.6304 |
|  | ZINC02106269 | Non-Toxic | Non-carcinogens | | Inhibitor | III/0.5662 | 2.6655 |
|  | ZINC02106282 | Non-Toxic | Non-carcinogens | | Inhibitor | III/0.5427 | 2.7301 |
|  | ZINC02107064 | Non-Toxic | Non-carcinogens | | Inhibitor | III/0.6642 | 2.6134 |
|  | ZINC02107810 | Non-Toxic | Non-carcinogens | | Non-Inhibitor | III/0.6018 | 2.4426 |
|  | ZINC02108288 | Non-Toxic | Non-carcinogens | | Inhibitor | III/0.5520 | 2.6396 |
|  | ZINC02108366 | Non-Toxic | Non-carcinogens | | Inhibitor | III/0.5884 | 2.7271 |
|  | ZINC02108756 | Non-Toxic | Non-carcinogens | | Non-Inhibitor | III/0.6084 | 2.6603 |
|  | ZINC02109073 | Non-Toxic | Non-carcinogens | | Inhibitor | III/0.6578 | 2.6805 |
|  | ZINC02109448 | Non-Toxic | Non-carcinogens | | Non-Inhibitor | III/0.5909 | 2.7458 |
|  | ZINC02109745 | Non-Toxic | Non-carcinogens | | Non-Inhibitor | III/0.6018 | 2.4426 |
|  | ZINC02111048 | Non-Toxic | Non-carcinogens | | Non-Inhibitor | III/0.5710 | 2.6542 |
|  | ZINC02111151 | Non-Toxic | Non-carcinogens | | Inhibitor | III/0.6578 | 2.6805 |
|  | ZINC02111981 | Non-Toxic | Non-carcinogens | | Inhibitor | III/0.5826 | 2.6256 |
|  | ZINC02112230 | Non-Toxic | Non-carcinogens | | Non-Inhibitor | III/0.6422 | 2.5587 |
|  | ZINC02112613 | Non-Toxic | Non-carcinogens | | Inhibitor | III/0.5716 | 2.6388 |
|  | ZINC02113936 | Non-Toxic | Non-carcinogens | | Non-Inhibitor | III/0.5905 | 2.7213 |
|  | ZINC02115702 | Non-Toxic | Non-carcinogens | | Non-Inhibitor | III/0.5630 | 2.6972 |
|  | ZINC02117133 | Toxic | Non-carcinogens | | Non-Inhibitor | III/0.6530 | 2.5759 |
|  | ZINC02117888 | Non-Toxic | Non-carcinogens | | Non-Inhibitor | III/0.5909 | 2.7458 |
|  | ZINC02118353 | Non-Toxic | Non-carcinogens | | Non-Inhibitor | III/0.5427 | 2.6139 |
|  | ZINC02118360 | Toxic | Non-carcinogens | | Non-Inhibitor | III/0.7061 | 2.3822 |
|  | ZINC02118901 | Toxic | Non-carcinogens | | Non-Inhibitor | III/0.7114 | 2.4742 |
|  | ZINC02120774 | Non-Toxic | Non-carcinogens | | Non-Inhibitor | III/0.6506 | 2.3612 |
|  | ZINC02122409 | Toxic | Non-carcinogens | | Non-Inhibitor | III/0.7061 | 2.3822 |
|  | **ZINC02123081** | Non-Toxic | Non-carcinogens | | Non-Inhibitor | II/0.7501 | 2.4221 |
|  | ZINC02123191 | Toxic | Non-carcinogens | | Non-Inhibitor | III/0.6989 | 2.5095 |
|  | ZINC02123282 | Non-Toxic | Non-carcinogens | | Non-Inhibitor | III/0.6552 | 2.2840 |
|  | ZINC02123424 | Non-Toxic | Non-carcinogens | | Non-Inhibitor | III/0.6355 | 2.6881 |
|  | ZINC02123859 | Non-Toxic | Non-carcinogens | | Non-Inhibitor | III/0.6180 | 2.6535 |
|  | ZINC02125038 | Toxic | Non-carcinogens | | Non-Inhibitor | III/0.7169 | 2.3843 |
|  | ZINC02125714 | Non-Toxic | Non-carcinogens | | Non-Inhibitor | III/0.6389 | 2.5430 |
|  | ZINC02148197 | Non-Toxic | Non-carcinogens | | Non-Inhibitor | III/0.6085 | 2.4223 |
|  | ZINC02148481 | Non-Toxic | Non-carcinogens | | Non-Inhibitor | III/0.6650 | 2.1956 |
|  | ZINC02148655 | Non-Toxic | Non-carcinogens | | Inhibitor | III/0.6651 | 2.6994 |
|  | ZINC02148909 | Non-Toxic | Non-carcinogens | | Inhibitor | III/0.5860 | 2.6701 |
|  | ZINC02148919 | Non-Toxic | Non-carcinogens | | Non-Inhibitor | III/0.6351 | 2.1841 |
|  | ZINC02148935 | Toxic | Non-carcinogens | | Non-Inhibitor | III/0.6565 | 2.5621 |
|  | ZINC02149492 | Toxic | Non-carcinogens | | Non-Inhibitor | III/0.6457 | 2.5892 |
|  | ZINC02150498 | Non-Toxic | Non-carcinogens | | Non-Inhibitor | III/0.6219 | 2.5999 |
|  | ZINC02151835 | Non-Toxic | Non-carcinogens | | Inhibitor | III/0.5650 | 2.7979 |
|  | ZINC02151836 | Non-Toxic | Non-carcinogens | | Inhibitor | III/0.5650 | 2.7979 |
|  | ZINC02154469 | Non-Toxic | Non-carcinogens | | Inhibitor | III/0.6578 | 2.6805 |
|  | ZINC02154866 | Non-Toxic | Non-carcinogens | | Inhibitor | III/0.5884 | 2.7271 |
|  | ZINC02155992 | Non-Toxic | Non-carcinogens | | Inhibitor | III/0.6645 | 2.6835 |
|  | ZINC02156445 | Non-Toxic | Non-carcinogens | | Non-Inhibitor | III/0.6511 | 2.6387 |
|  | ZINC02156984 | Non-Toxic | Non-carcinogens | | Inhibitor | III/0.5826 | 2.6256 |
|  | ZINC02156988 | Non-Toxic | Non-carcinogens | | Inhibitor | III/0.5826 | 2.6256 |
|  | ZINC02157224 | Non-Toxic | Non-carcinogens | | Non-Inhibitor | III/0.5892 | 2.6509 |
|  | ZINC02158043 | Non-Toxic | Non-carcinogens | | Inhibitor | III/0.6645 | 2.6835 |
|  | ZINC02158790 | Non-Toxic | Non-carcinogens | | Non-Inhibitor | III/0.4870 | 2.8512 |
|  | ZINC02159274 | Non-Toxic | Non-carcinogens | | Inhibitor | III/0.4908 | 2.5947 |
|  | ZINC02159526 | Toxic | Non-carcinogens | | Non-Inhibitor | III/0.6957 | 2.4006 |
|  | ZINC02159783 | Non-Toxic | Non-carcinogens | | Non-Inhibitor | III/0.6574 | 2.5521 |
|  | ZINC02160471 | Non-Toxic | Non-carcinogens | | Non-Inhibitor | III/0.6355 | 2.6881 |
|  | ZINC02503427 | Toxic | Non-carcinogens | | Inhibitor | III/0.7396 | 2.3568 |
|  | ZINC03842059 | Non-Toxic | Non-carcinogens | | Inhibitor | III/0.6106 | 2.5858 |
|  | ZINC03843035 | Non-Toxic | Non-carcinogens | | Inhibitor | III/0.7514 | 2.1973 |
|  | **ZINC03843365** | Non-Toxic | Non-carcinogens | | Non-Inhibitor | III/0.4688 | 2.3062 |
|  | ZINC03844760 | Non-Toxic | Non-carcinogens | | Non-Inhibitor | III/0.5944 | 2.4049 |
|  | ZINC03845096 | Non-Toxic | Non-carcinogens | | Non-Inhibitor | III/0.6494 | 2.7050 |
|  | **ZINC03845323** | Non-Toxic | Non-carcinogens | | Non-Inhibitor | III/0.6892 | 2.1979 |
|  | ZINC03845515 | Non-Toxic | Non-carcinogens | | Non-Inhibitor | III/0.6608 | 2.3246 |
|  | ZINC03845516 | Non-Toxic | Non-carcinogens | | Non-Inhibitor | III/0.6608 | 2.3246 |
|  | ZINC03846581 | Non-Toxic | Non-carcinogens | | Non-Inhibitor | III/0.7238 | 2.2855 |
|  | ZINC03846582 | Non-Toxic | Non-carcinogens | | Non-Inhibitor | III/0.7238 | 2.2855 |
|  | ZINC03846898 | Toxic | Non-carcinogens | | Inhibitor | III/0.5909 | 2.4557 |
|  | ZINC03847083 | Non-Toxic | Non-carcinogens | | Non-Inhibitor | III/0.7940 | 2.0451 |
|  | ZINC03847184 | Non-Toxic | Non-carcinogens | | Non-Inhibitor | III/0.4449 | 2.8975 |
|  | ZINC03848443 | Toxic | Non-carcinogens | | Non-Inhibitor | III/0.5718 | 2.5728 |
|  | ZINC03848917 | Toxic | Non-carcinogens | | Non-Inhibitor | III/0.7081 | 2.2499 |
|  | **ZINC03849421** | Non-Toxic | Non-carcinogens | | Non-Inhibitor | III/0.6044 | 2.3438 |
|  | ZINC03851871 | Non-Toxic | Non-carcinogens | | Non-Inhibitor | III/0.6346 | 2.2141 |
|  | ZINC03872986 | Non-Toxic | Non-carcinogens | | Non-Inhibitor | III/0.5500 | 2.7068 |
|  | ZINC03927200 | Non-Toxic | Non-carcinogens | | Non-Inhibitor | III/0.7290 | 1.9430 |
|  | ZINC03960761 | Toxic | Non-carcinogens | | Inhibitor | III/0.4740 | 2.8056 |
|  | ZINC03979002 | Non-Toxic | Non-carcinogens | | Non-Inhibitor | II/0.4696 | 2.8255 |
|  | ZINC04017533 | Non-Toxic | Non-carcinogens | | Non-Inhibitor | III/0.8139 | 2.1948 |
|  | ZINC04026127 | Non-Toxic | Non-carcinogens | | Non-Inhibitor | III/0.8062 | 1.8018 |
|  | ZINC04026666 | Non-Toxic | Non-carcinogens | | Non-Inhibitor | III/0.5171 | 2.7351 |
|  | ZINC04027261 | Non-Toxic | Non-carcinogens | | Non-Inhibitor | III/0.6030 | 2.7168 |
|  | ZINC04046706 | Non-Toxic | Non-carcinogens | | Non-Inhibitor | III/0.6719 | 2.2953 |
|  | ZINC04062050 | Non-Toxic | Non-carcinogens | | Inhibitor | III/0.6045 | 2.7222 |
|  | ZINC04073740 | Non-Toxic | Non-carcinogens | | Non-Inhibitor | III/0.6781 | 2.4875 |
|  | ZINC04084783 | Non-Toxic | Non-carcinogens | | Non-Inhibitor | III/0.6785 | 1.7441 |
|  | ZINC04084832 | Non-Toxic | Non-carcinogens | | Inhibitor | III/0.5525 | 2.5749 |
|  | ZINC04084888 | Toxic | Non-carcinogens | | Non-Inhibitor | III/0.5000 | 2.7962 |
|  | ZINC04084890 | Toxic | Non-carcinogens | | Non-Inhibitor | III/0.5000 | 2.7962 |
|  | ZINC04085127 | Non-Toxic | Non-carcinogens | | Non-Inhibitor | III/0.5844 | 2.3325 |
|  | ZINC04085162 | Toxic | Non-carcinogens | | Non-Inhibitor | III/0.4413 | 2.6741 |
|  | ZINC04085562 | Toxic | Non-carcinogens | | Non-Inhibitor | III/0.5271 | 2.4060 |
|  | ZINC04086945 | Non-Toxic | Non-carcinogens | | Non-Inhibitor | III/0.4719 | 2.5356 |
|  | **ZINC04090179** | Non-Toxic | Non-carcinogens | | Non-Inhibitor | III/0.6593 | 2.5593 |
|  | ZINC04090180 | Non-Toxic | Non-carcinogens | | Non-Inhibitor | III/0.6593 | 2.5593 |
|  | **ZINC04090428** | Non-Toxic | Non-carcinogens | | Non-Inhibitor | III/0.6457 | 2.8002 |
|  | ZINC04235972 | Non-Toxic | Non-carcinogens | | Non-Inhibitor | III/0.6509 | 2.4613 |
|  | ZINC04236421 | Non-Toxic | Non-carcinogens | | Inhibitor | III/0.6506 | 2.3650 |
|  | ZINC04237100 | Non-Toxic | Non-carcinogens | | Inhibitor | III/0.6093 | 2.3987 |
|  | ZINC04258893 | Non-Toxic | Non-carcinogens | | Inhibitor | III/0.6517 | 2.4222 |
|  | ZINC04258903 | Non-Toxic | Non-carcinogens | | Inhibitor | III/0.6449 | 2.3779 |
|  | ZINC04259264 | Non-Toxic | Non-carcinogens | | Non-Inhibitor | III/0.6151 | 2.2970 |
|  | ZINC04655273 | Non-Toxic | Non-carcinogens | | Non-Inhibitor | III/0.7960 | 1.9022 |
|  | **ZINC05220992** | Non-Toxic | Non-carcinogens | | Non-Inhibitor | III/0.4718 | 2.1161 |
|  | ZINC05397050 | Non-Toxic | Non-carcinogens | | Inhibitor | III/0.6088 | 2.6073 |
|  | ZINC05415240 | Non-Toxic | Non-carcinogens | | Non-Inhibitor | III/0.6336 | 2.3773 |
|  | ZINC06092274 | Toxic | Non-carcinogens | | Non-Inhibitor | III/0.7521 | 2.2665 |
|  | ZINC06137732 | Non-Toxic | Non-carcinogens | | Inhibitor | III/0.6113 | 2.4251 |
|  | ZINC06624582 | Non-Toxic | Non-carcinogens | | Non-Inhibitor | III/0.4481 | 2.3883 |
|  | ZINC06624588 | Non-Toxic | Non-carcinogens | | Non-Inhibitor | III/0.6579 | 2.3349 |
|  | ZINC06624612 | Non-Toxic | Non-carcinogens | | Non-Inhibitor | III/0.5919 | 2.3518 |
|  | ZINC08299978 | Non-Toxic | Non-carcinogens | | Non-Inhibitor | III/0.6485 | 2.1263 |
|  | ZINC08382456 | Non-Toxic | Non-carcinogens | | Non-Inhibitor | III/0.7392 | 2.2476 |
|  | ZINC08445572 | Non-Toxic | Non-carcinogens | | Non-Inhibitor | III/0.4975 | 2.2993 |
|  | ZINC08649676 | Non-Toxic | Non-carcinogens | | Non-Inhibitor | III/0.5898 | 2.4873 |
|  | ZINC08739084 | Non-Toxic | Non-carcinogens | | Non-Inhibitor | III/0.5898 | 2.3215 |
|  | ZINC08764694 | Non-Toxic | Non-carcinogens | | Non-Inhibitor | III/0.6668 | 2.1836 |
|  | ZINC08765261 | Non-Toxic | Non-carcinogens | | Non-Inhibitor | III/0.7259 | 2.7749 |
|  | ZINC08790130 | Non-Toxic | Non-carcinogens | | Inhibitor | III/0.5780 | 2.3738 |
|  | ZINC08791465 | Non-Toxic | Non-carcinogens | | Inhibitor | III/0.5427 | 2.4798 |
|  | ZINC08791487 | Non-Toxic | Non-carcinogens | | Non-Inhibitor | III/0.5678 | 2.5111 |
|  | ZINC08791488 | Non-Toxic | Non-carcinogens | | Non-Inhibitor | III/0.5678 | 2.5111 |
|  | ZINC08791610 | Non-Toxic | Non-carcinogens | | Inhibitor | III/0.6152 | 2.6364 |
|  | ZINC08792167 | Non-Toxic | Non-carcinogens | | Non-Inhibitor | III/0.6939 | 2.3985 |
|  | ZINC08792170 | Toxic | Non-carcinogens | | Non-Inhibitor | III/0.6992 | 2.1478 |
|  | ZINC08792350 | Non-Toxic | Non-carcinogens | | Non-Inhibitor | III/0.7710 | 2.3609 |
|  | ZINC08792429 | Non-Toxic | Non-carcinogens | | Non-Inhibitor | III/0.7617 | 2.2881 |
|  | ZINC08792433 | Toxic | Non-carcinogens | | Non-Inhibitor | III/0.7195 | 2.3553 |
|  | ZINC08877830 | Toxic | Non-carcinogens | | Non-Inhibitor | III/0.6882 | 2.5678 |
|  | ZINC08877855 | Toxic | Non-carcinogens | | Non-Inhibitor | III/0.6882 | 2.5678 |
|  | ZINC08877857 | Toxic | Non-carcinogens | | Non-Inhibitor | III/0.6894 | 2.3242 |
|  | ZINC08878911 | Non-Toxic | Non-carcinogens | | Non-Inhibitor | III/0.7430 | 2.2989 |
|  | ZINC08878978 | Non-Toxic | Non-carcinogens | | Non-Inhibitor | III/0.6855 | 2.5684 |
|  | ZINC08918127 | Non-Toxic | Non-carcinogens | | Inhibitor | III/0.6108 | 2.5736 |
|  | ZINC08918259 | Non-Toxic | Non-carcinogens | | Inhibitor | III/0.6556 | 2.3649 |
|  | **ZINC08918302** | Non-Toxic | Non-carcinogens | | Non-Inhibitor | III/0.5026 | 2.0506 |
|  | ZINC08918345 | Non-Toxic | Non-carcinogens | | Non-Inhibitor | III/0.5891 | 2.6515 |
|  | ZINC08991637 | Non-Toxic | Non-carcinogens | | Non-Inhibitor | III/0.6465 | 2.6355 |
|  | ZINC08993096 | Non-Toxic | Non-carcinogens | | Non-Inhibitor | III/0.6279 | 2.7143 |
|  | ZINC09034003 | Non-Toxic | Non-carcinogens | | Non-Inhibitor | III/0.6203 | 2.6957 |
|  | ZINC09034064 | Non-Toxic | Non-carcinogens | | Inhibitor | III/0.6844 | 2.5664 |
|  | ZINC09054134 | Non-Toxic | Non-carcinogens | | Non-Inhibitor | III/0.6116 | 2.6831 |
|  | ZINC09089656 | Toxic | Non-carcinogens | | Inhibitor | III/0.5505 | 2.3821 |
|  | **ZINC09303152** | Non-Toxic | Non-carcinogens | | Non-Inhibitor | III/0.5878 | 2.2406 |
|  | ZINC09341662 | Toxic | Non-carcinogens | | Non-Inhibitor | III/0.6427 | 2.5988 |
|  | ZINC09420846 | Non-Toxic | Non-carcinogens | | Non-Inhibitor | III/0.6226 | 2.6819 |
|  | ZINC09423653 | Non-Toxic | Non-carcinogens | | Non-Inhibitor | III/0.6397 | 2.6753 |
|  | ZINC09423883 | Toxic | Non-carcinogens | | Non-Inhibitor | III/0.5925 | 2.7596 |
|  | ZINC09423968 | Non-Toxic | Non-carcinogens | | Non-Inhibitor | III/0.6397 | 2.6753 |
|  | ZINC09576136 | Toxic | Non-carcinogens | | Non-Inhibitor | III/0.7096 | 2.3480 |
|  | ZINC11865187 | Non-Toxic | Non-carcinogens | | Non-Inhibitor | III/0.5912 | 2.5611 |
|  | ZINC11865349 | Non-Toxic | Non-carcinogens | | Inhibitor | III/0.6128 | 2.6682 |
|  | ZINC11865376 | Non-Toxic | Non-carcinogens | | Non-Inhibitor | III/0.6927 | 2.1998 |
|  | ZINC11866483 | Non-Toxic | Non-carcinogens | | Non-Inhibitor | III/0.5030 | 2.6582 |
|  | ZINC11866484 | Non-Toxic | Non-carcinogens | | Non-Inhibitor | III/0.5030 | 2.6582 |
|  | ZINC11866485 | Non-Toxic | Non-carcinogens | | Non-Inhibitor | III/0.3079 | 3.1049 |
|  | ZINC11866495 | Non-Toxic | Non-carcinogens | | Non-Inhibitor | III/0.3079 | 3.1049 |
|  | ZINC11867066 | Non-Toxic | Non-carcinogens | | Non-Inhibitor | III/0.6373 | 2.5081 |
|  | ZINC11867457 | Non-Toxic | Non-carcinogens | | Non-Inhibitor | III/0.5848 | 2.8440 |
|  | ZINC11867458 | Non-Toxic | Non-carcinogens | | Non-Inhibitor | III/0.5848 | 2.8440 |
|  | ZINC11867461 | Non-Toxic | Non-carcinogens | | Non-Inhibitor | III/0.3749 | 3.0359 |
|  | ZINC11867462 | Non-Toxic | Non-carcinogens | | Non-Inhibitor | III/0.3749 | 3.0359 |
|  | ZINC11867465 | Non-Toxic | Non-carcinogens | | Non-Inhibitor | III/0.6838 | 2.3687 |
|  | ZINC11867466 | Non-Toxic | Non-carcinogens | | Non-Inhibitor | III/0.6838 | 2.3687 |
|  | ZINC12296404 | Non-Toxic | Non-carcinogens | | Non-Inhibitor | III/0.5549 | 2.7601 |
|  | ZINC12296477 | Non-Toxic | Non-carcinogens | | Non-Inhibitor | III/0.6799 | 2.2929 |
|  | ZINC12296478 | Non-Toxic | Non-carcinogens | | Non-Inhibitor | III/0.6799 | 2.2929 |
|  | ZINC12296500 | Non-Toxic | Non-carcinogens | | Non-Inhibitor | III/0.3593 | 3.1167 |
|  | ZINC12296501 | Non-Toxic | Non-carcinogens | | Non-Inhibitor | III/0.3593 | 3.1167 |
|  | ZINC12296551 | Non-Toxic | Non-carcinogens | | Non-Inhibitor | III/0.3329 | 3.0035 |
|  | ZINC12296564 | Non-Toxic | Non-carcinogens | | Non-Inhibitor | III/0.6394 | 2.6579 |
|  | ZINC12296565 | Non-Toxic | Non-carcinogens | | Non-Inhibitor | III/0.6394 | 2.6579 |
|  | **ZINC12296580** | Non-Toxic | Non-carcinogens | | Non-Inhibitor | III/0.6788 | 2.6743 |
|  | ZINC12296581 | Non-Toxic | Non-carcinogens | | Non-Inhibitor | III/0.6788 | 2.6743 |
|  | ZINC12296700 | Non-Toxic | Non-carcinogens | | Non-Inhibitor | III/0.4726 | 2.9452 |
|  | ZINC12296701 | Non-Toxic | Non-carcinogens | | Non-Inhibitor | III/0.4726 | 2.9452 |
|  | ZINC12296761 | Non-Toxic | Non-carcinogens | | Non-Inhibitor | III/0.4236 | 2.7798 |
|  | ZINC12296762 | Non-Toxic | Non-carcinogens | | Non-Inhibitor | III/0.4236 | 2.7798 |
|  | ZINC12296864 | Non-Toxic | Non-carcinogens | | Non-Inhibitor | III/0.4204 | 2.9328 |
|  | ZINC12296876 | Non-Toxic | Non-carcinogens | | Non-Inhibitor | III/0.4620 | 2.7972 |
|  | ZINC12297013 | Non-Toxic | Non-carcinogens | | Non-Inhibitor | III/0.4620 | 2.7972 |
|  | ZINC12297014 | Non-Toxic | Non-carcinogens | | Non-Inhibitor | III/0.4620 | 2.7972 |
|  | ZINC12297019 | Non-Toxic | Non-carcinogens | | Non-Inhibitor | III/0.6991 | 2.4372 |
|  | **ZINC12297025** | Non-Toxic | Non-carcinogens | | Non-Inhibitor | III/0.4836 | 2.8042 |
|  | ZINC12297042 | Non-Toxic | Non-carcinogens | | Non-Inhibitor | III/0.5724 | 2.5770 |
|  | ZINC12297091 | Non-Toxic | Non-carcinogens | | Non-Inhibitor | III/0.5452 | 2.8645 |
|  | ZINC12297092 | Non-Toxic | Non-carcinogens | | Non-Inhibitor | III/0.5452 | 2.8645 |
|  | ZINC12442585 | Non-Toxic | Non-carcinogens | | Inhibitor | III/0.6377 | 2.5755 |
|  | ZINC12442587 | Non-Toxic | Non-carcinogens | | Inhibitor | III/0.6377 | 2.5755 |
|  | ZINC12442588 | Non-Toxic | Non-carcinogens | | Inhibitor | III/0.6127 | 2.6298 |
|  | ZINC12442590 | Non-Toxic | Non-carcinogens | | Inhibitor | III/0.6127 | 2.6298 |
|  | ZINC12442592 | Non-Toxic | Non-carcinogens | | Non-Inhibitor | III/0.6113 | 2.5996 |
|  | ZINC12442594 | Non-Toxic | Non-carcinogens | | Non-Inhibitor | III/0.6113 | 2.5996 |
|  | ZINC12442597 | Non-Toxic | Non-carcinogens | | Inhibitor | III/0.6340 | 2.5592 |
|  | ZINC12442599 | Non-Toxic | Non-carcinogens | | Inhibitor | III/0.6340 | 2.5592 |
|  | ZINC12442608 | Non-Toxic | Non-carcinogens | | Inhibitor | III/0.6123 | 2.6495 |
|  | ZINC12442610 | Non-Toxic | Non-carcinogens | | Inhibitor | III/0.6123 | 2.6495 |
|  | ZINC12442649 | Non-Toxic | Non-carcinogens | | Inhibitor | III/0.6482 | 2.5358 |
|  | ZINC12442671 | Non-Toxic | Non-carcinogens | | Non-Inhibitor | III/0.6658 | 2.6431 |
|  | ZINC12602816 | Non-Toxic | Non-carcinogens | | Inhibitor | III/0.6288 | 2.5841 |
|  | ZINC12604806 | Non-Toxic | Non-carcinogens | | Inhibitor | III/0.6216 | 2.3768 |
|  | ZINC12660190 | Non-Toxic | Non-carcinogens | | Inhibitor | III/0.6054 | 2.6406 |
|  | ZINC12863193 | Non-Toxic | Non-carcinogens | | Inhibitor | III/0.6041 | 2.5321 |
|  | ZINC12863203 | Non-Toxic | Non-carcinogens | | Inhibitor | III/0.6041 | 2.5321 |
|  | ZINC12866401 | Non-Toxic | Non-carcinogens | | Inhibitor | III/0.5947 | 2.5527 |
|  | ZINC12870752 | Non-Toxic | Non-carcinogens | | Non-Inhibitor | III/0.5428 | 2.7397 |
|  | ZINC12887203 | Non-Toxic | Non-carcinogens | | Non-Inhibitor | III/0.7012 | 2.4359 |
|  | ZINC12892129 | Non-Toxic | Non-carcinogens | | Non-Inhibitor | III/0.6862 | 2.3080 |
|  | ZINC12893265 | Non-Toxic | Non-carcinogens | | Non-Inhibitor | III/0.6268 | 2.1243 |
|  | ZINC12893333 | Non-Toxic | Non-carcinogens | | Non-Inhibitor | III/0.6093 | 2.1469 |
|  | ZINC12899484 | Non-Toxic | Non-carcinogens | | Non-Inhibitor | III/0.4204 | 2.9328 |
|  | ZINC12899490 | Non-Toxic | Non-carcinogens | | Non-Inhibitor | III/0.4204 | 2.9328 |
|  | ZINC13359662 | Toxic | Non-carcinogens | | Non-Inhibitor | III/0.6784 | 2.3882 |
|  | ZINC13551971 | Non-Toxic | Non-carcinogens | | Inhibitor | III/0.5942 | 2.6065 |
|  | ZINC13553109 | Non-Toxic | Non-carcinogens | | Inhibitor | III/0.5622 | 2.7242 |
|  | ZINC13683907 | Non-Toxic | Non-carcinogens | | Inhibitor | III/0.6482 | 2.5358 |
|  | ZINC13687938 | Non-Toxic | Non-carcinogens | | Non-Inhibitor | III/0.6155 | 2.5185 |
|  | ZINC13692664 | Non-Toxic | Non-carcinogens | | Non-Inhibitor | III/0.6605 | 2.7313 |
|  | ZINC13720979 | Non-Toxic | Non-carcinogens | | Non-Inhibitor | III/0.6375 | 2.5790 |
|  | ZINC13721203 | Non-Toxic | Non-carcinogens | | Non-Inhibitor | III/0.6321 | 2.5795 |
|  | ZINC13731223 | Non-Toxic | Non-carcinogens | | Non-Inhibitor | III/0.6658 | 2.6431 |
|  | ZINC13732746 | Non-Toxic | Non-carcinogens | | Non-Inhibitor | III/0.6375 | 2.5790 |
|  | ZINC13732895 | Toxic | Non-carcinogens | | Non-Inhibitor | III/0.6044 | 2.8391 |
|  | ZINC13736331 | Non-Toxic | Non-carcinogens | | Non-Inhibitor | III/0.6234 | 2.6260 |
|  | ZINC15303561 | Non-Toxic | Non-carcinogens | | Non-Inhibitor | III/0.5697 | 2.4545 |
|  | ZINC15303564 | Non-Toxic | Non-carcinogens | | Non-Inhibitor | III/0.5697 | 2.4545 |
|  | ZINC15957613 | Non-Toxic | Non-carcinogens | | Inhibitor | III/0.6583 | 2.3390 |
|  | ZINC15959322 | Non-Toxic | Non-carcinogens | | Inhibitor | III/0.6171 | 2.4240 |
|  | ZINC15959323 | Non-Toxic | Non-carcinogens | | Inhibitor | III/0.6171 | 2.4240 |
|  | ZINC15959325 | Non-Toxic | Non-carcinogens | | Inhibitor | III/0.6171 | 2.4240 |
|  | ZINC15967689 | Non-Toxic | Non-carcinogens | | Non-Inhibitor | III/0.6012 | 2.4461 |
|  | ZINC15967691 | Non-Toxic | Non-carcinogens | | Non-Inhibitor | III/0.6012 | 2.4461 |
|  | ZINC15967694 | Non-Toxic | Non-carcinogens | | Non-Inhibitor | III/0.6012 | 2.4461 |
|  | ZINC15969172 | Non-Toxic | Non-carcinogens | | Inhibitor | III/0.6171 | 2.4240 |
|  | ZINC15969174 | Non-Toxic | Non-carcinogens | | Inhibitor | III/0.6171 | 2.4240 |
|  | ZINC15969569 | Non-Toxic | Non-carcinogens | | Inhibitor | III/0.5892 | 2.5266 |
|  | ZINC15969571 | Non-Toxic | Non-carcinogens | | Inhibitor | III/0.5892 | 2.5266 |
|  | ZINC15969573 | Non-Toxic | Non-carcinogens | | Inhibitor | III/0.5892 | 2.5266 |
|  | ZINC16026354 | Non-Toxic | Non-carcinogens | | Inhibitor | III/0.6659 | 2.4167 |
|  | **ZINC16292828** | Non-Toxic | Non-carcinogens | | Non-Inhibitor | III/0.5878 | 2.2406 |
|  | ZINC16972169 | Non-Toxic | Non-carcinogens | | Inhibitor | III/0.5973 | 2.1704 |
|  | ZINC16995797 | Non-Toxic | Non-carcinogens | | Inhibitor | III/0.6239 | 2.3243 |
|  | ZINC17014720 | Non-Toxic | Non-carcinogens | | Inhibitor | III/0.6123 | 2.2880 |
|  | ZINC17015252 | Non-Toxic | Non-carcinogens | | Inhibitor | III/0.6315 | 2.4159 |
|  | ZINC17029354 | Non-Toxic | Non-carcinogens | | Inhibitor | III/0.6230 | 2.2477 |
|  | ZINC17194529 | Non-Toxic | Non-carcinogens | | Inhibitor | III/0.6101 | 2.2181 |
|  | ZINC17195517 | Non-Toxic | Non-carcinogens | | Inhibitor | III/0.6123 | 2.2880 |
|  | ZINC17196472 | Non-Toxic | Non-carcinogens | | Inhibitor | III/0.6105 | 2.2614 |
|  | ZINC17196754 | Non-Toxic | Non-carcinogens | | Inhibitor | III/0.6425 | 2.2948 |
|  | ZINC18091170 | Non-Toxic | | Non-carcinogens | Non-Inhibitor | III/0.4975 | 2.2993 |
|  | ZINC18176990 | Non-Toxic | | Non-carcinogens | Non-Inhibitor | III/0.5961 | 2.1391 |
|  | ZINC18207570 | Non-Toxic | | Non-carcinogens | Inhibitor | III/0.6101 | 2.2181 |
|  | ZINC18219349 | Non-Toxic | | Non-carcinogens | Non-Inhibitor | III/0.6381 | 2.2967 |
|  | ZINC19891473 | Non-Toxic | | Non-carcinogens | Inhibitor | III/0.6659 | 2.4167 |
|  | ZINC20411267 | Non-Toxic | | Non-carcinogens | Inhibitor | III/0.6230 | 2.2477 |
|  | ZINC20411826 | Non-Toxic | | Non-carcinogens | Inhibitor | III/0.6239 | 2.3243 |
|  | ZINC20412340 | Non-Toxic | | Non-carcinogens | Inhibitor | III/0.6230 | 2.2477 |
|  | ZINC20611122 | Non-Toxic | | Non-carcinogens | Inhibitor | III/0.6171 | 2.4240 |
|  | ZINC31169877 | Non-Toxic | | Non-carcinogens | Non-Inhibitor | III/0.6174 | 2.6560 |
|  | ZINC32502304 | Non-Toxic | | Non-carcinogens | Inhibitor | III/0.6225 | 2.5540 |
|  | ZINC32502306 | Non-Toxic | | Non-carcinogens | Inhibitor | III/0.5966 | 2.5356 |
|  | ZINC34300440 | Non-Toxic | | Non-carcinogens | Non-Inhibitor | III/0.5585 | 2.8007 |
|  | ZINC35365995 | Non-Toxic | | Non-carcinogens | Non-Inhibitor | III/0.6294 | 2.4616 |
|  | ZINC35365997 | Non-Toxic | | Non-carcinogens | Non-Inhibitor | III/0.6294 | 2.4616 |
|  | ZINC35457807 | Non-Toxic | | Non-carcinogens | Non-Inhibitor | III/0.4546 | 2.6131 |
|  | ZINC36369885 | Non-Toxic | | Non-carcinogens | Non-Inhibitor | III/0.5717 | 2.5197 |
|  | ZINC38167070 | Non-Toxic | | Non-carcinogens | Non-Inhibitor | III/0.5545 | 2.2330 |
|  | ZINC38167083 | Non-Toxic | | Non-carcinogens | Non-Inhibitor | III/0.5097 | 2.5004 |
|  | ZINC54278174 | Non-Toxic | | Non-carcinogens | Non-Inhibitor | III/0.6167 | 2.4853 |
|  | ZINC54278181 | Non-Toxic | | Non-carcinogens | Non-Inhibitor | III/0.6202 | 2.5561 |
|  | ZINC54278191 | Non-Toxic | | Non-carcinogens | Non-Inhibitor | III/0.6411 | 2.5935 |
|  | ZINC54333105 | Non-Toxic | | Non-carcinogens | Inhibitor | III/0.6117 | 2.6259 |
|  | ZINC54333106 | Non-Toxic | | Non-carcinogens | Inhibitor | III/0.6117 | 2.6259 |
|  | ZINC55396209 | Non-Toxic | | Non-carcinogens | Non-Inhibitor | III/0.6878 | 2.5487 |
|  | ZINC58090636 | Toxic | | Non-carcinogens | Non-Inhibitor | III/0.6752 | 2.5584 |
|  | ZINC61997665 | Non-Toxic | | Non-carcinogens | Non-Inhibitor | III/0.6189 | 2.5308 |
|  | ZINC61997666 | Non-Toxic | | Non-carcinogens | Non-Inhibitor | III/0.6189 | 2.5308 |
|  | ZINC61997705 | Non-Toxic | | Non-carcinogens | Non-Inhibitor | III/0.6198 | 2.6234 |
|  | ZINC62001268 | Non-Toxic | | Non-carcinogens | Inhibitor | III/0.6727 | 2.5645 |
|  | ZINC67913130 | Non-Toxic | | Non-carcinogens | Non-Inhibitor | III/0.5265 | 2.1843 |
|  | ZINC68569286 | Non-Toxic | | Non-carcinogens | Non-Inhibitor | II/0.5612 | 3.4719 |
|  | **ZINC68569602** | Non-Toxic | | Non-carcinogens | Non-Inhibitor | III/0.6209 | 2.1810 |
|  | ZINC68572120 | Non-Toxic | | Non-carcinogens | Non-Inhibitor | I/0.7129 | 4.0666 |
|  | **ZINC68583170** | Non-Toxic | | Non-carcinogens | Non-Inhibitor | III/0.4737 | 2.3784 |
|  | ZINC68583182 | Non-Toxic | | Non-carcinogens | Non-Inhibitor | III/0.5833 | 2.3009 |
|  | ZINC68589458 | Non-Toxic | | Non-carcinogens | Non-Inhibitor | III/0.6806 | 2.2444 |
|  | ZINC68589462 | Non-Toxic | | Non-carcinogens | Non-Inhibitor | III/0.6806 | 2.2444 |
|  | ZINC68591854 | Non-Toxic | | Non-carcinogens | Non-Inhibitor | III/0.7268 | 2.4642 |
|  | ZINC68591857 | Non-Toxic | | Non-carcinogens | Non-Inhibitor | III/0.7268 | 2.4642 |

**Supplementary Table S5.** Summary of binding affinity with interacting residues of the top 14 compounds with control compound 4E3 obtained from molecular docking studies by four docking tools: Autodock Tools, Autodock Vina, Smina and idock. The Residues which involved in hydrogen bonding were highlighted in bold as well as selected hits for MDS are also highlighted in bold. The Autodock, Vina, Smina and iDock column represents the binding affinity of all the ligands.

| **Sr. No.** | **Compound**  **ID** | **IC_50_** | **Auto**  **Dock** | **No. of**  **H.**  **bonds** | **Residues** | **AutoDockVina** | **No. of H bond** | **Residues** | **Smina** | **No. of H bond** | **Residues** | **iDock** | **No. of H bond** | **Residues** |
| --- | --- | --- | --- | --- | --- | --- | --- | --- | --- | --- | --- | --- | --- | --- |
|  | 4E3 | 97.87 nM | -9.56 | 2 | Ile165,Gly166,Lys167,Val173,Phe170,**Lys188**,**Asp307**,Glu203,Ala186,Phe238,Val306,Val222,Leu294,Leu241 | -9.9 | 3 | Ile165,Val166,Val173,Phe170,Lys167,**Lys188**,Ala186,Phe238,Met240,Leu241,Val222,Leu294,Val306,**Asp307** | -9.9 | 3 | Ile165,Gly166,Lys167,Phe170,Val173,**Lys188**,Phe238,Ala186,Met240,Leu241,Val222,Leu294,Val306,**Asp307** | -10.11 | 3 | Ile165,Gly166,Lys167,Phe170,Val173,**Lys188**,Ala186,Met240,Leu241,Phe238,Val222,Leu294,Val306,**Asp307** |
|  | **ZINC_2123081** | 9.72  nM | -10.93 | 4 | Ile165,Val173,Ala186,Met240,**Leu241**,Lys188,Val222,Phe238,Val306,Leu294,**Asn244**,**Asp247**,Tyr243 | -12.1 | 2 | Gly166,Val173,Ala186,Phe170,Lys188,Asn292,Phe238,**Asp307**,Val306,Val222,Leu294,Leu241,**Lys167** | -12.5 | 1 | Ile165,Val173,Ala186,Lys188,Phe238,Asp307,Val306,Val222,Leu294,**Leu241**,Met240,Asn244,Tyr243,Asp247 | -12.32 | 1 | Ile165,Val173,Ala186,Lys188,Phe238,Asp307,Val306,Val222,Leu294,**Leu241**,Met240,Asn244,Tyr243,Asp247 |
|  | **ZINC_3843365** | 7.75  nM | -11.07 | 2 | Ile165,Gly166,Val173,Phe170,**Lys188**,Ala186,**Asp307**,Glu203,Phe238,Val306,Val222,Leu294,Leu241 | -12.5 | 1 | Ile165,Gly166,Val173,Phe170,Ala186,**Lys188**,Phe238,Asp307,Val306,Val222,Leu294,Leu241 | -12.9 | 1 | Ile165,Gly166,Val173,Phe170,**Lys188**,Ala186,Phe238,Asp307,Val306,Val222,Leu294,Leu241 | -13.09 | 1 | Ile165,Gly166,Val173,Phe170,Ala186,**Lys188**,Phe238,Asp307,Val306,Val222,Leu294,Leu241 |
|  | ZINC_3845323 | 27.39  nM | -10.32 | 3 | Ile165,Phe238,Val173,**Lys188**,**Asp307**,Val306,Val222,Asn244,Asp247,Leu294,Tyr243,Ser242,Leu241,Met240 | -11.7 | 0 | Ile165,Gly166,Gly168,Val173,Ser169,Phe170,Ala186,Phe238,Asp307,Asn292,Val306,Val222,Leu294,Leu294 | -11.7 | 0 | Ile165,Gly166,Val173,Gly168,Ser169,Phe170,Ala186,Phe238,Asn292,Asp307,Val306,Val222,Leu294,Leu241 | -11.90 | 0 | Ile165,Gly166,Gly168,Val173,Ser169,Phe170,Ala186,Phe238,Asn292,Asp307,Val306,Val222,Leu294,Leu241 |
|  | ZINC_3849421 | 286.75  nM | -8.93 | 0 | Phe170,Lys188,Gly166,Val173,Ile165,Phe238,Ala186,Met240,Leu241,Val222,Leu294,Val306 | -11.7 | 0 | Ile165,Gly166,Lys167,Val173,Phe170,Ala186,Lys188,Phe238,Asp307,Val306,Val222,Leu294,Leu241 | -11.7 | 0 | Ile165,Gly166,Lys167,Phe170,Val173,Lys188,Ala186,Leu241,Phe238,Val222,Val306,Leu294,Asp307 | -11.82 | 0 | Ile165,Lys167,Gly166,Val173,Phe170,Ala186,Lys188,Phe238,Asp307,Val306,Val222,Leu294,Leu241 |
|  | ZINC_4090179 | 53.64  nM | -9.92 | 3 | Ile165,Gly166,Val173,Phe170,Ala186,**Lys188**,Phe238,**Asp307**,**Glu203**,Val306,Val222,Leu294,Leu241 | -12.2 | 1 | Ile165,Gly166,Val173,Phe170,Ala186,Lys188,Phe238,**Asp307**,Asn292,Val306,Val222,Leu294,Leu241 | -12.2 | 1 | Ile165,Gly166,Val173,Phe170,Lys188,Ala186,**Asp307**,Asn292,Phe238,Val306,Val222,Leu294,Leu241 | -12.29 | 2 | Ile165,Gly166,Val173,Ala186,Phe170,**Lys188**,Phe238,**Asp307**,Asn292,Val306,Val222,Leu294,Leu241 |
|  | ZINC_4090428 | 130.67  nM | -9.39 | 2 | Ile165,Val173,Phe170,**Lys188**,Ala186,Phe238,**Asp307**,Glu203,Asn292,Val306,Val222,Leu294,Leu241 | -11.6 | 1 | Ile165,Gly166,**Lys167**,Val173,Phe170,Ala186,Lys188,Phe238,Asp307,Glu203,Val306,Val222,Leu294,Leu241 | -11.6 | 1 | Ile165,Gly166,**Lys167**,Val173,Phe170,Ala186,Lys188,Phe238,Asp307,Glu203,Val306,Val222,Leu294,Leu241 | -11.67 | 1 | Ile165,Gly166,**Lys167**,Val173,Phe170,Ala186,Lys188,Phe238,Asp307,Glu203,Val306,Val222,Leu294,Leu241 |
|  | **ZINC_5220992** | 16.23  nM | -10.63 | 2 | Ile165,Val173,Ala186,Phe238,Met240,**Leu241**,Ser242,Val222,Leu294,Val306,Tyr243,Asp247 | -13.3 | 0 | Ile165,Val173,Ala186,Lys188,Phe238,Asp307,Val306,Leu294,Leu241,Ser242,Tyr243,Asp247 | -13.3 | 0 | Ile165,Val173,Ala186,Leu241,Phe238,Lys188,Val306,Asp307,Leu294,Asp247,Ser242,Tyr243 | -13.50 | 0 | Ile165,Val173,Ala186,Lys188,Phe238,Asp307,Val306,Leu294,Leu241,Ser242,Tyr243,Asp247 |
|  | ZINC_8918302 | 15.47 nM | -10.66 | 0 | Gly166,Val173,Ile165,Lys188,Ala186,Phe238,Met240,Leu241,Val222,Ser242,Leu294,Val306,Asp307 | -13.3 | 0 | Ile165,Tyr243,Val173,Leu294,Lys188,Val306,Asp307,Phe238,Ala186,Leu241,Ser242 | -13.3 | 0 | Ile165,Val173,Ala186,Lys188,Phe238,Asp307,Val306,Leu294,Leu241,Ser242,Tyr243 | -13.53 | 0 | Ile165,Val173,Ala186,Phe238,Lys188,Val306,Asp307,Leu294,Leu241,Ser242,Tyr243 |
|  | ZINC_9303152 | 100.77 nM | -9.55 | 0 | Ile165,Gly166,Lys167,Val173,Gly168,Phe170,Tyr243,Ser242,Leu294,Val306,Phe238,Val222,Ala186,Met240,Leu241 | -12.1 | 0 | Ile165,Gly166,Lys167,Gly168,Val173,Phe170,Glu291,Phe238,Val306,Val222,Leu294,Leu241,Met240,Ser242,Tyr243 | -12.1 | 0 | Ile165,Gly166,Lys167,Gly168,Val173,Phe170,Glu291,Phe238,Val306,Val222,Leu294,Leu241,Met240,Ser242,Tyr243 | -12.14 | 0 | Ile165,Gly166,Lys167,Gly168,Val173,Phe170,Ala186,Glu291,Phe238,Val306,Val222,Leu294,Leu241,Ser242,Tyr243 |
|  | ZINC_12296580 | 17.56 nM | -10.58 | 3 | Ile165,Val173,Gly166,Phe170,**Lys188**,Glu203,Ala186,Phe238,Met240,**Leu241**,Val222,Leu294,Val306,Tyr243,**Asp307**,Asp247 | -11.7 | 3 | Ile165,Gly166,Gly168,Val173,Phe170,**Lys188**,Ala186,Phe238,**Asp307**,Val306,Val222,Leu294,**Leu241**,Met240 | -11.7 | 3 | Ile165,Gly166,Val173,Gly168,Phe170,**Lys188**,Ala186,Phe238,**Asp307**,Val306,Val222,Leu294,**Leu241**,Met240 | -11.92 | 3 | Ile165,Gly166,Gly168,Ser169,Val173,Phe170,**Lys188**,Ala186,Phe238,**Asp307**,Val306,Val222,Leu294,**Leu241**,Met240 |
|  | ZINC_12297025 | 31.88 nM | -10.23 | 3 | Ile165,Gly166,Val173,Phe170,**Lys188**,**Asp307**,Glu203,Phe238,Val306,Val222,Leu294,**Leu241**,Met240,Ala186,Tyr243 | -11.7 | 3 | Ile165,Gly166,Lys167,Val173,Gly168,Phe170,**Lys188**,Ala186,Phe238,**Asp307**,Val306,Val222,Leu294,**Leu241**,Met240 | -11.7 | 3 | Ile165,Gly166,Lys167,Gly168,Val173,Phe170,**Lys188**,Ala186,Phe238,**Asp307**,Val306,Val222,Leu294,**Leu241**,Met240 | -11.88 | 3 | Ile165,Lys167,Gly168,Val173,Phe170,**Lys188**,Phe238,**Asp307**,Val306,Val222,Leu294,**Leu241**,Met240,Ala186 |
|  | ZINC_16292828 | 611.31 nM | -8.48 | 0 | Ile165,Gly166,Val173,Phe170,Lys188,Asn292,Asp307,Phe238,Val306,Val222,Leu294,Leu241,Glu291,Ser242,Asn244,Tyr243 | -9.4 | 0 | Gly166,Lys167,Gly168,Val173,Ser169,Phe170,Lys188,Asn292,Val306,Leu294,Glu291,Asn244,Tyr243 | -9.4 | 0 | Gly166,Lys167,Gly168,Val173,Ser169,Phe170,Lys188,Asn292,Val306,Leu294,Glu291,Asn244,Tyr243 | -9.52 | 1 | Gly166,Lys167,Gly168,Val173,Ser169,Phe170,Lys188,Asn292,Val306,Leu294,Glu291,**Asn244**,Tyr243 |
|  | **ZINC_68569602** | 26.01 nM | -10.35 | 1 | Ile165,Gly166,Lys167,Gly168,Val173,Phe170,Lys188,Ala186,Phe238,Asp307,Val306,Val222,Leu294,**Leu241**,Met240,Ser242,Tyr243 | -12.3 | 2 | Ile165,Val173,Phe238,Lys188,Phe170,Asp307,Val306,Asn292,Glu291,Leu294,**Asn244**,Tyr243 | -12.3 | 2 | Ile165,Val173,Phe170,Lys188,Phe238,Asp307,Asn292,Val306,Glu291,Leu294,**Asn244**,Tyr243 | -12.49 | 2 | Ile165,Val173,Phe170,Lys188,Phe238,Val306,Asp307,Leu294,Glu291,**Asn244**,Tyr243 |
|  | ZINC_68583170 | 197.98 nM | -9.15 | 2 | Ile165,Gly166,Lys167,Val173,Phe170,**Lys188**,Phe238,**Asp307**,Glu203,Val306,Val222,Leu294,Glu291,Asn244,Ser242,Tyr243 | -9.5 | 1 | Gly166,Lys167,Val173,Gly168,Lys188,Phe170,**Ser169**,Val306,Asn292,Lys289,Glu291,Leu294,Asn244,Tyr243 | -9.5 | 1 | Gly166,Lys167,Gly168,Val173,**Ser169**,Phe170,Lys188,Lys289,Asn292,Val306,Leu294,Glu291,Asn244,Tyr243 | -9.75 | 1 | Gly166,Lys167,Gly168,Val173,**Ser169**,Phe170,Lys188,Lys289,Asn292,Val306,Leu294,Glu291,Asn244,Tyr243 |
