# Supplementary material for: Disruption of DYRK1A-induced hyperphosphorylation of amyloid-beta and tau protein in Alzheimer’s disease: An integrative molecular modeling approach
Source: Front Mol Biosci. 2023 Jan 19;9:1078987. doi: 10.3389/fmolb.2022.1078987 (PMC9892649; doi:10.3389/fmolb.2022.1078987)
Supplement: Supplementary file 2 [file DataSheet1.docx]

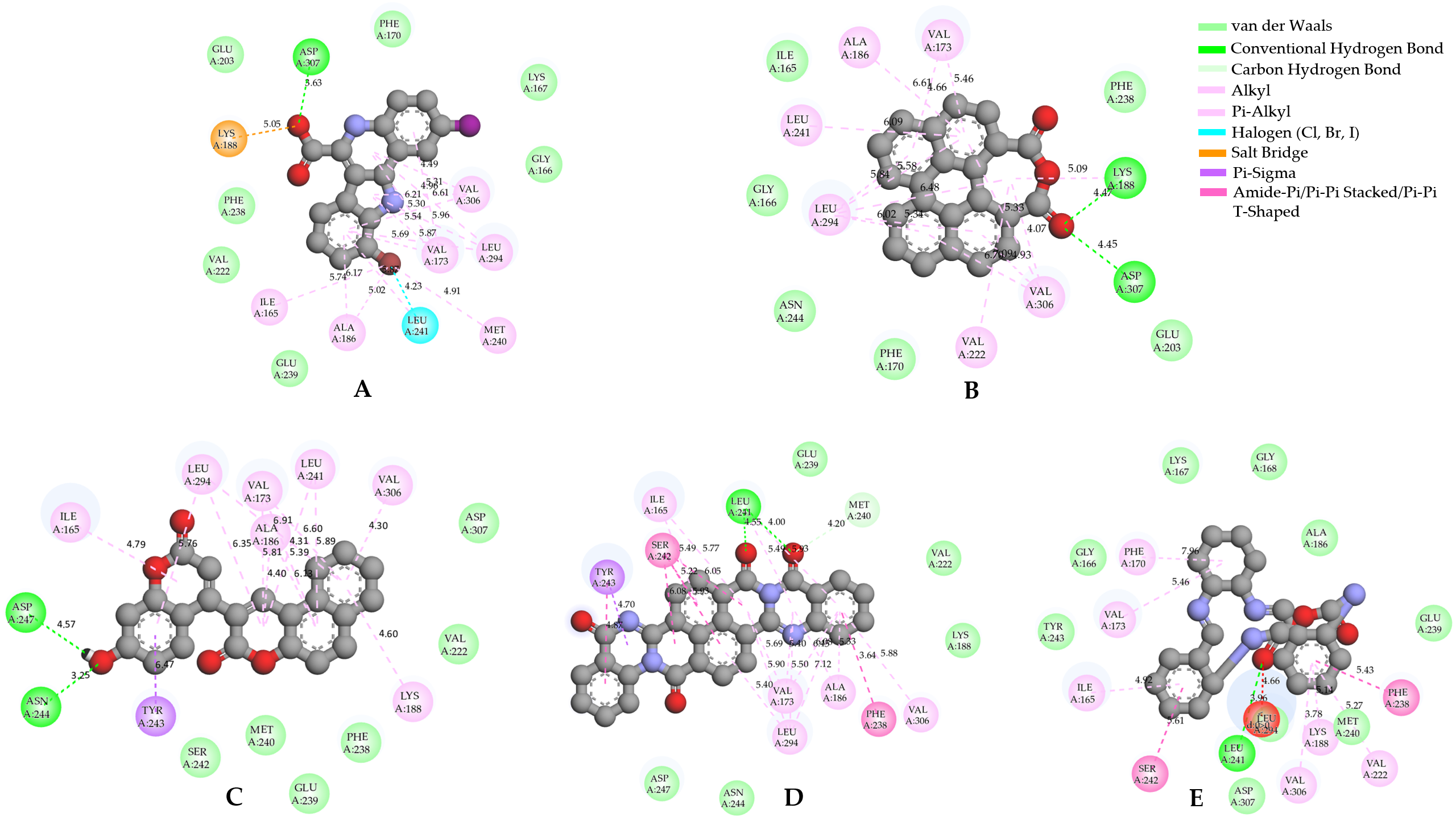


**Supplementary Figure S1**. 2D representation of molecular interaction between DYRK1A and ligand molecules using Discovery Studio program depicting van der Waals, Hydrogen Bond, carbon hydrogen bond, alkyl, Pi-alkyl, Pi-sigma, halogen (Cl, Br, I), salt bridge, and amide-Pi/Pi-Pi stacked/Pi-Pi T-shaped interactions: (A) 4E3 control; (B) ZINC3843365; (C) ZINC2123081; (D) ZINC5220992; and (E) ZINC68569602.


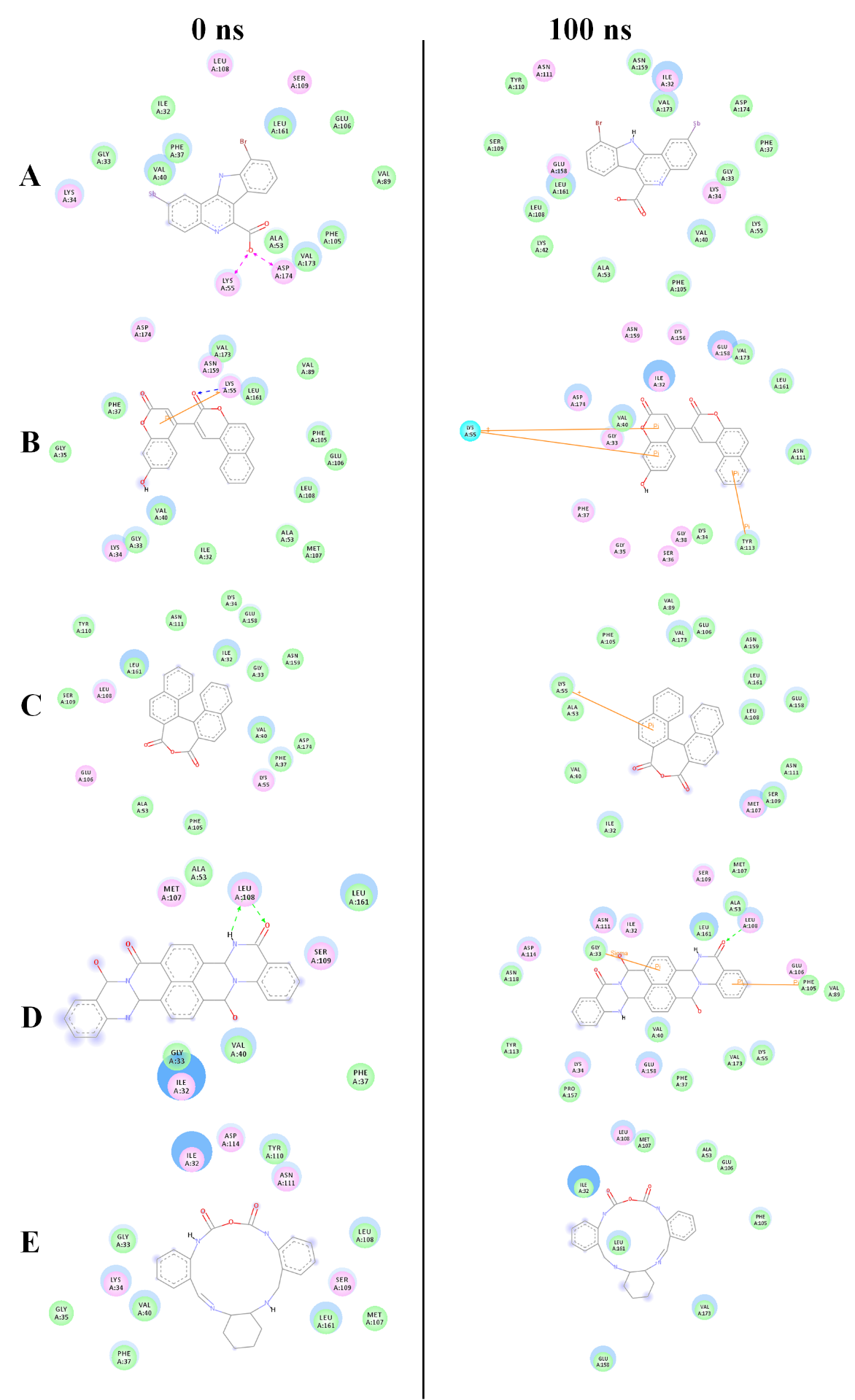


**Supplementary Figure S2**. 2D ligand interaction diagram obtained from 0 ns and 100 ns snapshots. (A) 4E3 control, (B) ZINC2123081, (C) ZINC3843365, (D) ZINC5220992, and (E) ZINC68569602.
